# Piercing Molecular Graphenes: Precision Synthesis and Photophysics of NBN-Edged Porous Molecular Carbons

**DOI:** 10.1021/jacs.5c06175

**Published:** 2025-05-28

**Authors:** Yang Yu, Asier Izu, José M. Marín Beloqui, Shammi Rana, Kunal S. Mali, Steven De Feyter, David Casanova, Juan Casado, Junzhi Liu

**Affiliations:** † Department of Chemistry, HKU-CAS Joint Laboratory on New Materials and Shanghai-Hong Kong Joint Laboratory on Chemical Synthesis, 25809 The University of Hong Kong, Hong Kong 999077, P. R. China; ‡ State Key Laboratory of Synthetic Chemistry, The University of Hong Kong, Hong Kong 999077, P. R. China; § 226245 Donostia International Physics Center (DIPC), Donostia, Euskadi 20018, Spain; ∥ Polimero eta Material Aurreratuak: Fisika, Kimika eta Teknologia, Kimika Fakultatea, UPV/EHU, PK 1072, Donostia, Euskadi 20018, Spain; ⊥ Department of Physical Chemistry, University of Malaga, Campus de Teations s/n, Malaga 229071, Spain; # Division of Molecular Imaging and Photonics, Department of Chemistry, KU Leuven, Celestijnenlaan 200F, Leuven 3001, Belgium; ∇ IKERBASQUE, Basque Foundation for Science, Bilbao, Euskadi 48009, Spain; ○ Materials Innovation Institute for Life Sciences and Energy (MILES), HKU-SIRI, Shenzhen 518048, P. R. China

## Abstract

Bottom-up solution-phase synthesis of atomically precise porous nanographenes is a challenging endeavor. In particular, molecular carbons with multiple pores and heteroatoms remain unknown. Herein, we report three porous molecular carbons (**2PNG**, **3PNG**, and **7PNG**) with precise NBN-doped zigzag edges, in which **7PNG** possesses seven pores. X-ray crystallographic diffraction and scanning tunneling microscopy reveal their unique pore structures and self-assembly behaviors. Interestingly, the HOMO–LUMO overlap of these molecules gradually decreases as the size of the molecule increases, which induces peripheral-to-core excitations and promotes intersystem crossing. Steady-state and transient spectroscopy, along with DFT calculations, reveal the excited-state dynamics and the size-dependent energy-transfer mechanism in these NBN-doped molecular systems. Our study describes a new strategy for producing minimal wave function overlaps at almost planar geometry by segmenting the electronic structures of molecular graphene by insertion of pores, forcing the excitation to occur between the periphery and the core, with great potential for new phosphorescent and delayed fluorescence emitters.

## Graphical abstract

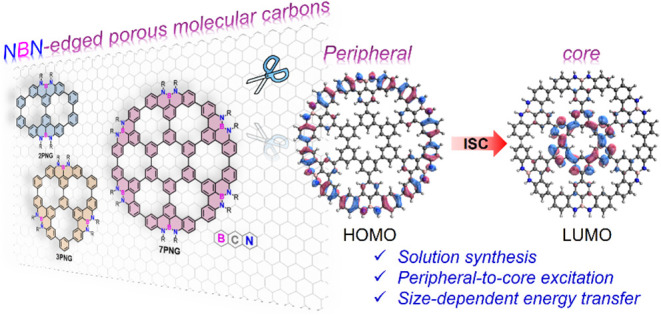

## Introduction

The tuning of the electronic properties of graphene from semimetallic to semiconducting has been extensively investigated. In general, the band structure of graphene can be modified in several ways, by doping,^1^ hydrogenation,^2^ cutting into strips,^3^ making nanomeshes,^4^ etc. Graphene nanomeshes, in particular, have been shown to be viable schemes to open the bandgap of graphene, with the uniqueness that can be tailored by the diameter and the surface density of the pores. This versatility has enabled multiple applications, encompassing electronic and photonic devices, including light-emitting diodes, field-effect transistors, biosensors, memories, photodetectors, lasers, spintronics, energy devices, etc.^4–8^

State-of-the-art *e*-beam lithography has been developed to carve graphene into nanoribbons and nanomeshes with feature sizes down to nanometers.^3,4,9^ However, this “top-down” approach is intrinsically limited by their ultimate resolution and precision at the atomic level. In contrast, “bottom-up” organic synthesis provides an efficient strategy to fabricate atomically precise molecular nanographenes with controlled sizes, shapes, and edge structures, as well as the amount and position of doping heteroatoms.^10–15^

Nanographenes containing geometrically defined pores (porous nanographenes, PNGs) can be viewed as model structures of porous graphene and graphene nanomeshes and have attracted significant interest in recent years owing to their unique topological structures, controlled assembly behaviors, and exotic properties.^10,16–23^ Over the past few years, great efforts have been made to prepare PNGs with variable bandgap^24^ and tunable electronic properties^25,26^ through surface-assisted synthesis and *in situ* characterization by scanning tunneling microscopy (STM),^21,27–33^ a strategy which is insufficient to preparing large quantities of materials and to further transferring them onto other substrates to fabricate electronic devices. In recent years, Isobe’s group reported a class of geodesic phenine frameworks with effectively introduced pores in nanocarbons (Figure 1a,**A**).^34–37^ In 2018, Kuck and Chow’s group synthesized a three-pore nonplanar twisted nanographene based on Scholl macrocyclization (Figure 1a,**B**).^38^ Highly twisted nonplanar porous molecular carbons have shown excellent flexibility and unique self-assembly properties.^18,39–41^ However, only a few ring-fused planar PNGs with armchair-edged structures have been synthesized and characterized to date.^21,22,42–44^ In 2016, Müllen’s group synthesized a planar coronoid nanographene in solution, which revealed an increased energy gap compared with its parent nanographene (Figure 1b,**C**).^42^ In 2020, Tan’s group synthesized a molecular defect-containing bilayer nanographene, which exhibited a 9.6-fold enhanced brightened emission compared with its same-sized defect-free counterpart (Figure 1b,**D**).^43^ In 2024, they further reported a PNG with an N-doped cavity, which possessed selective binding to Ag^+^ ion (Figure 1c,**E**).^44^ In 2025, Würthner’s group reported a selective halide permeation through an imide-substituted porous bilayer nanographene (Figure 1c,**F**).^22^ However, the limited structural intercorrelations between these cases precluded structure–property relationships, a situation well exemplified in the case of the largely unexplored photophysical properties of porous molecular carbons. To progress along these lines, it is crucial to synthesize molecular carbons with atomically precise defects (e.g., exact pores and heteroatoms) that are structurally interconnected.

**Figure 1:**
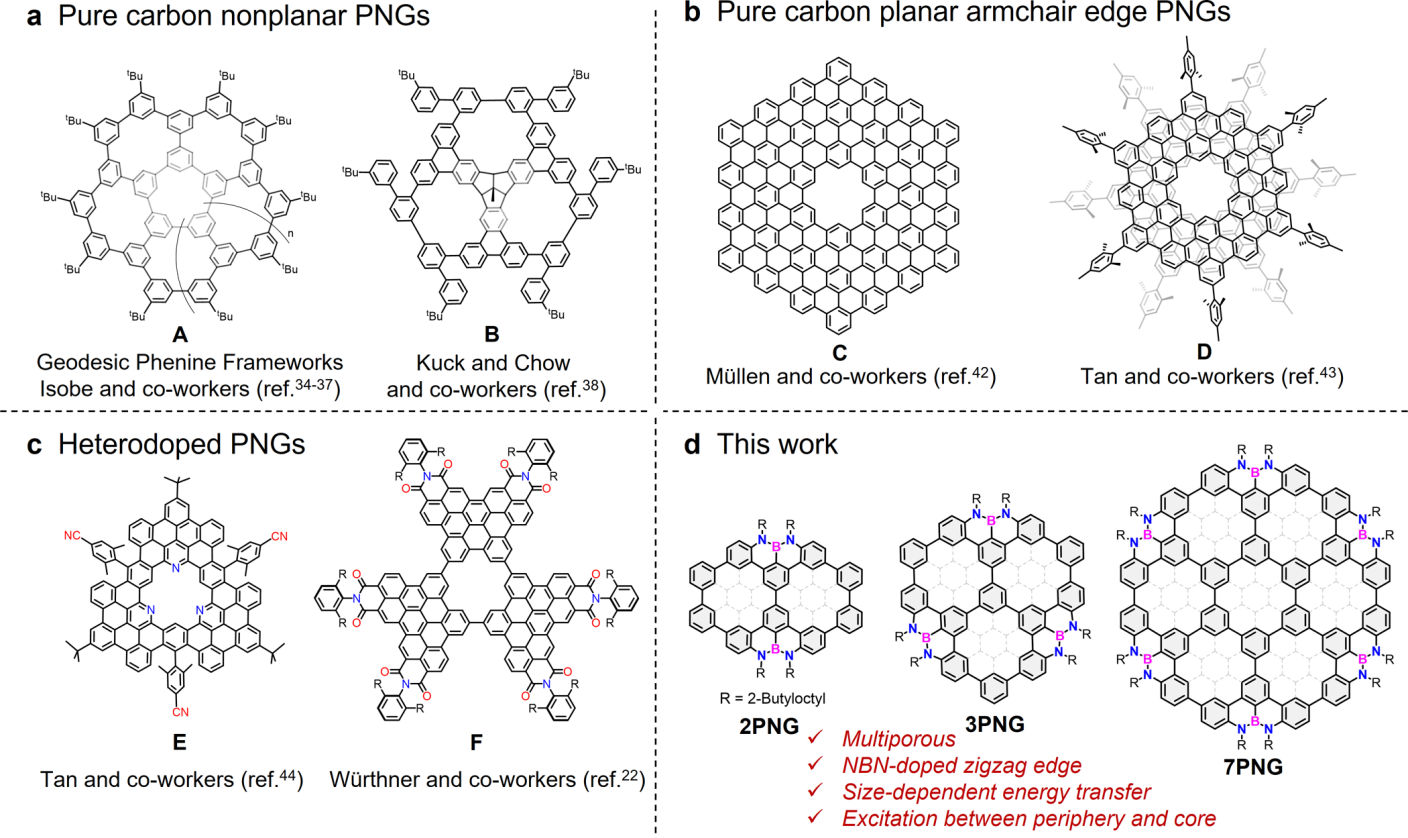
Chemical structures of solution-phase synthesized porous nanocarbons. (a, b) Previously reported representative pure carbon nonplanar PNGs (a) and planar armchair edge PNGs (b). (c) Previously reported heterodoped PNGs. (d) Structures of the NBN-doped zigzag-edged porous molecular carbons in this work.

Herein, we present a bottom-up solution-phase synthesis of three related molecular carbons (**2PNG**, **3PNG**, and **7PNG**, Figure 1d) featuring precise NBN-doped zigzag edge structures and well-defined pores. The resultant structures can be considered as cutout structures from NBN-doped graphene by removing two, three, and seven benzene rings from their corresponding full carbon framework, respectively. The pore structures of these molecules are unambiguously confirmed by single-crystal X-ray diffraction analysis, demonstrating the slightly twisted structures and intrinsic cavities in their carbon frameworks, whereas their supramolecular assembly properties are studied by STM on graphite with the molecules deposited from solution. Density functional theory (DFT) calculations revealed that these molecular carbons display unusual frontier molecular orbital (FMO) distributions with optical gaps over 3.0 eV. The spatial distribution of the highest occupied molecular orbital (HOMO, in the molecular periphery) and lowest unoccupied molecular orbital (LUMO, in the central molecular part) gradually segregates as the size of the molecule increases from **2PNG**, **3PNG** to **7PNG**, documenting minimal wave function overlap in **7PNG** which is significant when occurring at an almost planar geometry. As a result, the HOMO → LUMO optical excitation triggers out periphery → core intramolecular exciton transference between electronically diverging moieties, which originates a cascade of photophysical events of relevance. Steady-state and transient spectroscopy, along with time-dependent DFT (TDDFT) calculations revealed the size dependence of excited-state dynamics and the energy transfer mechanisms. This study introduces new molecular carbon-based chromophores modified through molecular pore insertion and selective heteroatom doping, providing an atomically precise model for segregated peripheral-to-core excitations in polycyclic compounds of increasing dimension. The analysis of the structure–photophysical relations will help to understand and design novel nanocarbon structures with potential applications in nanophotonics.

## Results and Discussion

### Molecular Design and Synthesis

Wave function confinement jointly promoted by insertion of pores and heteroatom substitution has dramatic consequences on the optical properties of the resulting molecules, in particular, the modulation of the optical gap, the emergence of emission properties (i.e., absent in nonporous extended nanographenes owing to the vanishing or very narrow band gap) and the excited state dynamics. This strategy aims to identify the optimal composition of pores and heteroatoms, wherein their synergistic interplay can enhance the properties of these unique structures. Following this blueprint, as shown in Scheme 1, we designed a bottom-up synthetic strategy toward porous molecular carbons, which entailed the initial construction of a star-shaped core with extended polycyclic aromatic “arms”, followed by the closure of the components to form the corresponding macrocycles bearing well-defined pores.^34–37,45,46^ Starting from the core synthons **9**, **13**, and **17**, the precursors of the targets can be synthesized through a divergent approach by stepwise addition of building blocks. The final one-step multicyclization ensures the achievement of the target compounds.

**Scheme 1:**
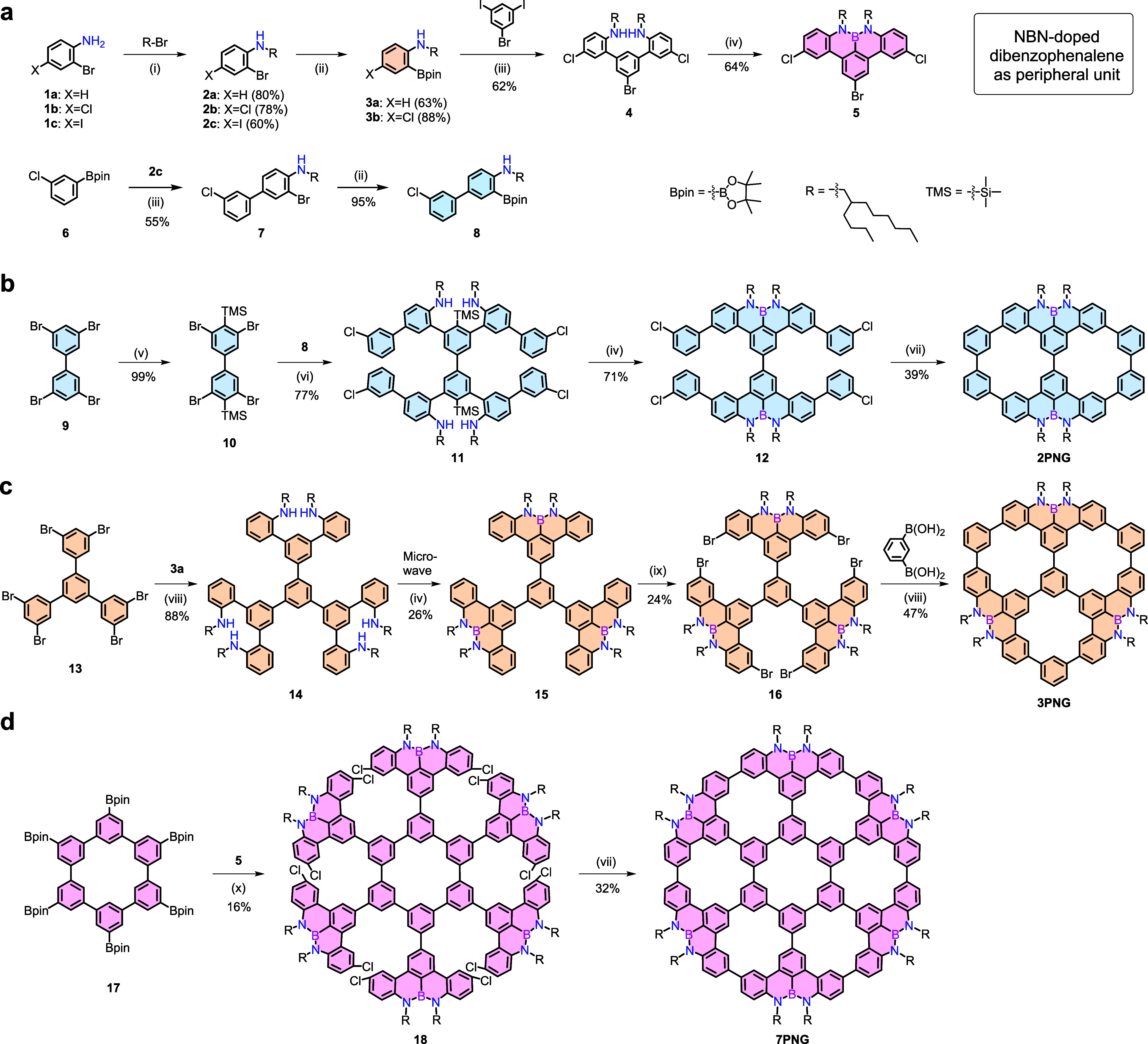
Synthesis of Porous Molecular Carbons^a^ ^b^

a (a) Synthesis of the key peripheral building blocks of compounds **3**, **5**, and **8**. (b–d) Synthetic routes to the double-cyclic **2PNG** (b), triple-cyclic **3PNG** (c), and septuple-cyclic **7PNG** (d).
b Reaction conditions (i) Sodium hydride, tetrahydrofuran (THF), 65 °C, 12 h; (ii) 1,1′-Bis­(diphenylphosphino)­ferrocene dichloropalladium­(II), bis­(pinacolato)­diboron, potassium acetate, dimethyl sulfoxide, 85 °C, 12 h; (iii) Tetrakis­(triphenylphosphine)­palladium(0) (Pd­(PPh_3_)_4_), potassium carbonate (K_2_CO_3_), THF/H_2_O, 55 °C, 48 h; (iv) Boron trichloride, triethylamine, *o*-dichlorobenzene, 180 °C, 8 h; (v) Trimethylsilyl chloride, lithium diisopropylamide, THF, −78 °C, 1 h; (vi) Pd­(PPh_3_)_4_, sodium bicarbonate, dimethoxyethane/H_2_O, 90 °C, 24 h; (vii) 2,2′-Bipyridine, 1,5-cyclooctadiene, bis­(1,5-cyclooctadiene)­nickel(0), toluene/dimethylformamide, 85 °C, 24 h; (viii) Pd­(PPh_3_)_4_, cesium carbonate, toluene/ethanol/H_2_O, 100 °C, 36 h; (ix) *N*-bromosuccinimide, dichloromethane/acetic acid, 25 °C, 24 h; (x) 1,1′-Bis (di-t-butylphosphino)­ferrocene palladium dichloride, K_2_CO_3_, THF/H_2_O, 80 °C, 48 h.

As shown in Scheme 1a, we synthesized three “arms” stepwise (compounds **8**, **3a**, and **5**) to suit the preferred plan for preparing **2PNG**, **3PNG**, and **7PNG**, respectively. To ensure the solubility of the final products, we introduced a branched 2-butyloctyl group in the synthesis of the NBN-edged peripheral unit. The targeted **2PNG** was synthesized in four steps from **9**, as depicted in Scheme 1b. First, **9** was selectively lithiated using lithium diisopropylamide and then quenched with trimethylsilyl (TMS) chloride to give quantitative **10** containing two TMS groups. Then, 4-fold palladium-catalyzed Suzuki couplings of the “arm” **8** with **10** provided the unfused intermediate **11** in a yield of 77%, in which the TMS groups act as a directing group for the next step electrophilic aromatic borylation. Compound **11** was treated with boron trichloride (BCl_3_) and triethylamine (Et_3_N) at 180 °C to furnish the NBN-edged fused precursor **12** in a yield of 71%, which was afterward converted into **2PNG** (39% yield) via Yamamoto coupling of the preorganized *m*-chloropenylene units. For **3PNG**, as shown in Scheme 1c, we first synthesized core synthon **13**, which has poor solubility at low temperatures and cannot introduce TMS groups. A 6-fold palladium-catalyzed Suzuki coupling of the “arm” **3a** with **13** gave the star-shaped intermediate **14** in a yield of 88%, then followed by an electrophilic borylation by the treatment of **14** with BCl_3_ and Et_3_N at 180 °C under the condition of microwave to form **15** (26% yield) with three NBN-edged peripheral units. Subsequently, **15** was successfully brominated with six equivalents of N-bromosuccinimide to furnish the precursor **16** in a yield of 24%, allowing for further π-extension. Finally, we close the peripheral units through a 6-fold Suzuki coupling between **16** and 1,3-benzenediboronic acid to achieve the fully conjugated **3PNG** with a benzene core and a wheel-shaped outer rim in a yield of 47%. For **7PNG**, at the initial synthetic planning level, the scarce yield for preparing the 6-fold electrophilic borylation forced us to defer the synthetic path similar to the one of **3PNG** and prefabricate the NBN-edged peripheral unit (**5**) formation in an earlier step (Scheme 1a). So, we first synthesized the 2-bromo-8,9-bis­(2-butyloctyl)-5,12-dichloro-8*H*,9*H*-8,9-diaza-8a-borabenzo­[*fg*]­tetracene (**5**, 64% yield) as an outer-rim unit in four steps. Then, we synthesized the central borylated macrocycle **17** ^47^ as the core and subjected it to annulative π-extension. As shown in Scheme 1d, six rim units **5** were selectively Suzuki coupled with the core **17** to afford chlorinated precursor **18** in a yield of 16%. The final cyclization to form **7PNG** was performed using Yamamoto coupling, with 6-fold intramolecular homocoupling reactions affording the target product (32% yield). As a result, a huge multicyclic porous nanographene **7PNG** was completed. The chemical structures of these porous molecular carbons were unambiguously confirmed with NMR spectroscopy and high-resolution mass spectrometry (Figures S31–S82).

### X-ray Crystallographic Structures Analysis

Single crystal structures unambiguously reveal the nature of the exact pores and defined N–B–N zigzag-edged periphery of **2PNG** and **3PNG** (Figures 2 and S1–S7 and Table S1). **2PNG** is crystallized in a triclinic *P-1* space group without solvent molecules and its structure consists of two linked NBN-doped dibenzophenalenes (NBN-DBPs) with the *meta*-linked phenylenes, resulting in a relatively planar, only slightly twisted, conjugated system. As a result, the structure of **2PNG** approximates a plane containing two inner pores, which creates [18]­annulene inner cavities with diameters of 5.90 Å and 5.92 Å, respectively (Figure 2a). There are two molecules in a unit cell as a dimer with an interlayer distance of 6.95 Å (Figure 2c), which ruled out the possibility of π-π interaction, likely due to the steric hindrance imposed by branched alkyl chains that lie perpendicular to the **2PNG** core and fill the free space between the dimers in the crystal. **3PNG** crystallized in a monoclinic *P21* space group with weak π-π interaction between the two molecules of a dimer in the unit cell, with an interlayer distance of 3.99 Å (Figure 2d), though it has the same alkyl chains as **2PNG**. However, due to the steric hindrance imposed by alkyl chains and the interaction between the terminal benzene ring of one pore and the carbon skeleton of the other, it displayed an approximately flat-bowl structure containing three inner pores with the diameters of 5.92, 5.96, and 5.92 Å, respectively (Figure 2b). The resultant double-concave structure of **3PNG** hinders further π-π stacking beyond dimerization. Meanwhile, the “face-to-face” stacking layers of **3PNG** show more significant slippage compared to the stacking of **2PNG** (Figures S2–S7). Relatively, based on the star-shaped triangular core and extended stability, **3PNG** shows a continuous π-π stacking effect and forms an alternatively stacked lamellar structure (Figures S5–S7).

**Figure 2:**
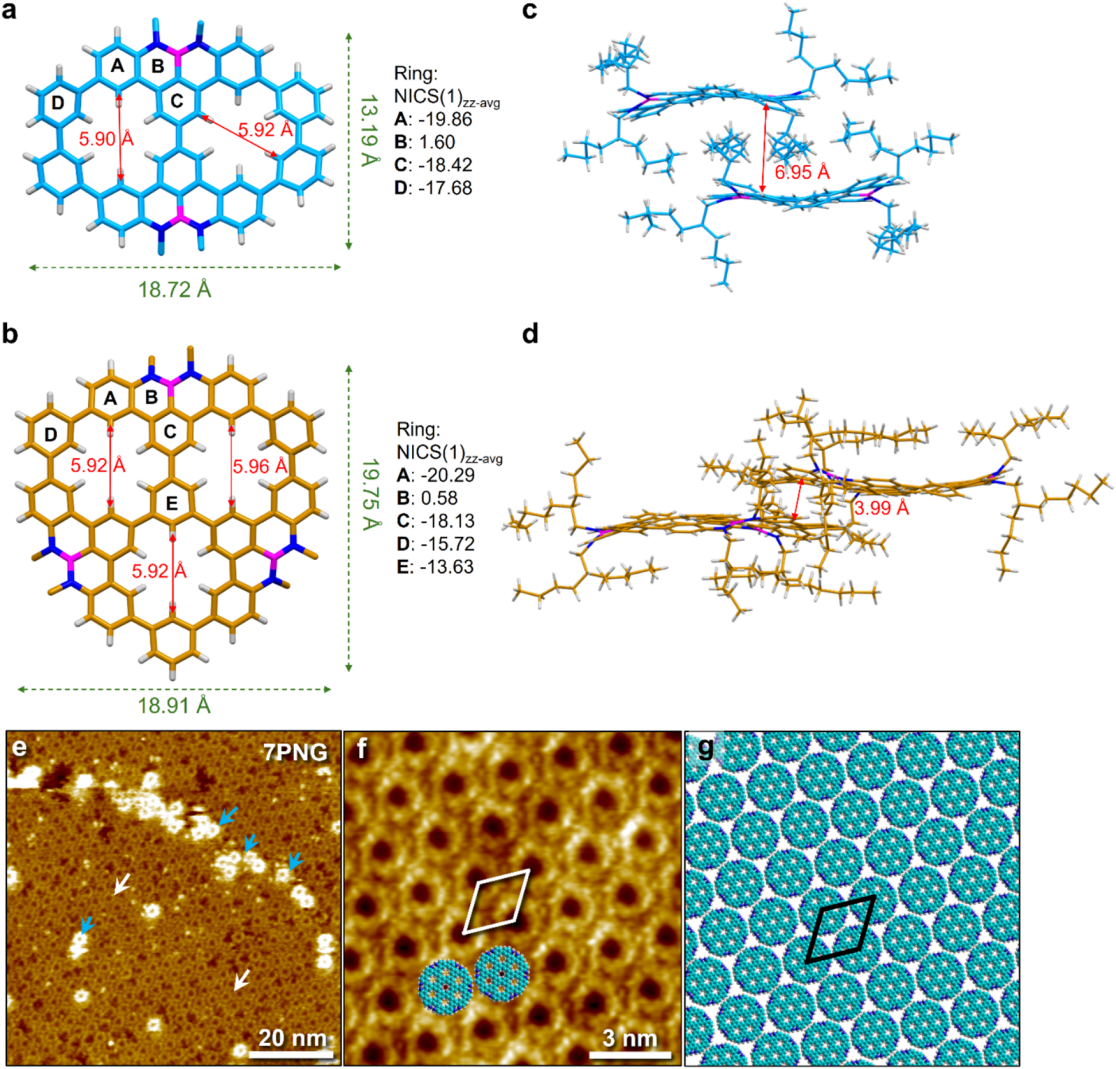
X-ray crystallographic molecular structures and STM study. (a, b), Single crystal structures of **2PNG** (a) and **3PNG** (b), which show the molecular size (green number), their diameters of inner pores (red number), and calculated NICS(1)_zz‑avg_ values (black number) of PNGs (at the GIAO-B3LYP/6–311+G­(2d,p) level of theory). Branched alkyl chains are replaced by methyl groups for clarity. (c, d), Packing patterns in a unit cell of **2PNG** (c) and **3PNG** (d), and the vertical distance between the molecular dimers. (e), Large-scale STM image of **7PNG** self-assembled at the 1-phenyloctane/graphite interface. Scale bar = 20 nm. White arrows indicate molecules adsorbed in the bottom layer, whereas blue arrows highlight molecules in the second layer. (f), Smaller-scale STM image of the self-assembled molecular networks of **7PNG**. Scale bar = 3 nm. [**7PNG**] = 160 μM. For imaging parameters, see the experimental section. (g), Tentative molecular models depicting the observed symmetry and arrangement of molecules within the self-assembled networks. These molecular models are based on experimentally obtained lattice parameters, which in turn were obtained from calibrated STM data. The peripheral alkyl chains have been omitted for the sake of clarity and, in almost all cases, are only partially adsorbed on the graphite surface. For **2PNG**, **3PNG**, and additional STM images and data of **7PNG**, see Supporting Information Section 4.

The bond lengths of **2PNG** and **3PNG** are shown in Figures S8–S9. The peripheral B–N bond lengths at the zigzag-edged range from 1.417 to 1.467 Å (average approximately 1.447 Å), which are consistent with the analogous bonds in typical NBN-embedded polycyclic aromatic hydrocarbons (PAHs)^48^ and shorter than the analogous bonds in typical BN-embedded PAHs (1.45–1.47 Å), featuring localized BN double bonds character.^49^ The lengths of the benzo-extended hexagons (rings A and C) in NBN-DBPs units and the *meta*-linked independent benzene rings (rings D and E) ranged from 1.303–1.459 Å, indicating typical benzene rings, consistent with the negative values of the calculated nucleus-independent chemical shifts (NICS(1)_zz‑avg_) (Figure 2a,b), and supported by the obvious clockwise diatropic ring currents in the anisotropy of current-induced density (ACID) plots (Figure S10), showing aromaticity of these carbon skeletons. In contrast, the NICS values of the BN-doped hexagons sharply decreased to near zero (ring B), indicating a nonaromatic ring. Intermolecular interactions of **2PNG** and **3PNG** were visualized by noncovalent interaction (NCI) analysis (Figures S11–S14). **2PNG** shows extensive CH···π and van der Waals interactions, as seen in the NCI plot of green isosurfaces (i.e., without intermolecular π–π interactions). On the other hand, **3PNG** shows intermolecular offset π–π interactions, which may contribute to the electronic communication in the layer and potentially be beneficial for one-dimensional charge transport.

### Self-Assembly of PNGs at the Solution-Solid Interface

The on-surface self-assembly behavior of the porous molecular carbons was studied under ambient conditions using STM at the 1-phenyloctane/graphite interface. Figure 2e shows a relatively large-scale STM image of a self-assembled molecular network (SAMN) formed by **7PNG**, which exhibits peculiar assembly behavior. After forming a complete physisorbed monolayer at the 1-phenyloctane/graphite interface (white arrows), additional molecules of **7PNG** adsorb atop the first layer (blue arrows) in contact with the substrate. The pronounced tendency to form a bilayer in the case of **7PNG** aligns with its larger π-surface compared to **2PNG** and **3PNG** systems (Figures S15–S19).

For a detailed STM study of **2PNG** and **3PNG**, see Figures S15–S17. Curiously, the **7PNG** molecules adsorbed on both the bottom and top layers appear as bright rings with a dark cavity in the center, as clearly evident in the high-resolution STM image of the monolayer in Figure 2f. We ascribe the bright ring features to the strong electronic conjugation of the **7PNG** molecules. The areas around the rings are ascribed to the regions where the peripheral alkyl chains are adsorbed. The branched alkyl chains are not resolved, possibly due to their fast dynamics on the time scale of STM imaging. Figure 2g shows a molecular model that was built using the unit cell parameters obtained from calibrated STM data, which reproduces the symmetry and arrangement of features observed in the STM image. Under the experimental conditions used here, a partial second layer with varying densities of **7PNG** molecules was observed (Figures 2e and S16c). Based on STM data, we conclude that the **7PNG** molecules in this second layer are adsorbed in an approximately cofacial manner with respect to the molecules in the bottom layer. This could be due to the planarization of PNG molecules upon adsorption on the graphite surface due to favorable π–π interactions. Such planarization of (poly)­aromatic molecules upon surface adsorption has been reported previously.^50^ We suggest that the peculiar STM contrast observed for **7PNG**, wherein the molecular backbone merely appears as a bright ring with no contrast features in the center, is in line with the strong electronic conjugation of the outer rim, as predicted by electronic structure calculations (see below).

### Electronic Structure from Quantum Chemical Calculations

#### Frontier Molecular Orbitals and Periphery-to-Core Segregation

The optimized geometry and FMOs are shown in Figures 3 and S20–S21. For the three molecules, a significant contribution from the N atoms is noticeable for the HOMOs with negligible involvement of the B atoms. Further, these HOMOs are well distributed over the electron-rich NBN-DBP moieties, including the peripheral benzene rings connecting them. Indeed, in **7PNG**, the HOMOs delocalize over the entire peripheral skeleton, or outer rim, with strong conjugation. The distribution of the LUMOs is similar on **2PNG** and **3PNG**, being mostly on the center or core of the molecules, with the NBN atomic triad negligibly contributing. The spatial disparity between occupied and virtual orbitals expands noticeably with the increasing size of the molecule, resulting in vanishing overlap between HOMO (periphery) and LUMO (porous core) in **7PNG**. The HOMO/LUMO energy levels of **2PNG**, **3PNG**, and **7PNG**, calculated at the B3LYP/6–311G­(2d,p) level (Figure S20), are −5.24/–1.56, −5.20/–1.60, and −4.94/–1.72 eV, respectively, corresponding to orbital energy gaps of 3.68, 3.60, and 3.22 eV. These values are consistent with the trends of optical energy gaps (*E*_g_^opt^) obtained from the absorption spectra: 3.06 eV for **2PNG**, 3.04 eV for **3PNG**, and 2.90 eV for **7PNG**. Compared to the nonporous nanographene analogues (computed at the M06–2*X*/6–31G­(d) level, Figure S21), the pores in the **2PNG**, **3PNG**, and **7PNG** reduce the extent of π-conjugation (resulting in more significant HOMO–LUMO gaps), enforce the distinct periphery-center localization of FMOs (inducing CT transitions), and prevent strong couplings through the carbon sp^2^ π-system. These results suggest that introducing pores and heteroatom doping effectively modifies the band structure of graphene, potentially enabling desirable optical properties in porous molecular carbons.

**Figure 3:**
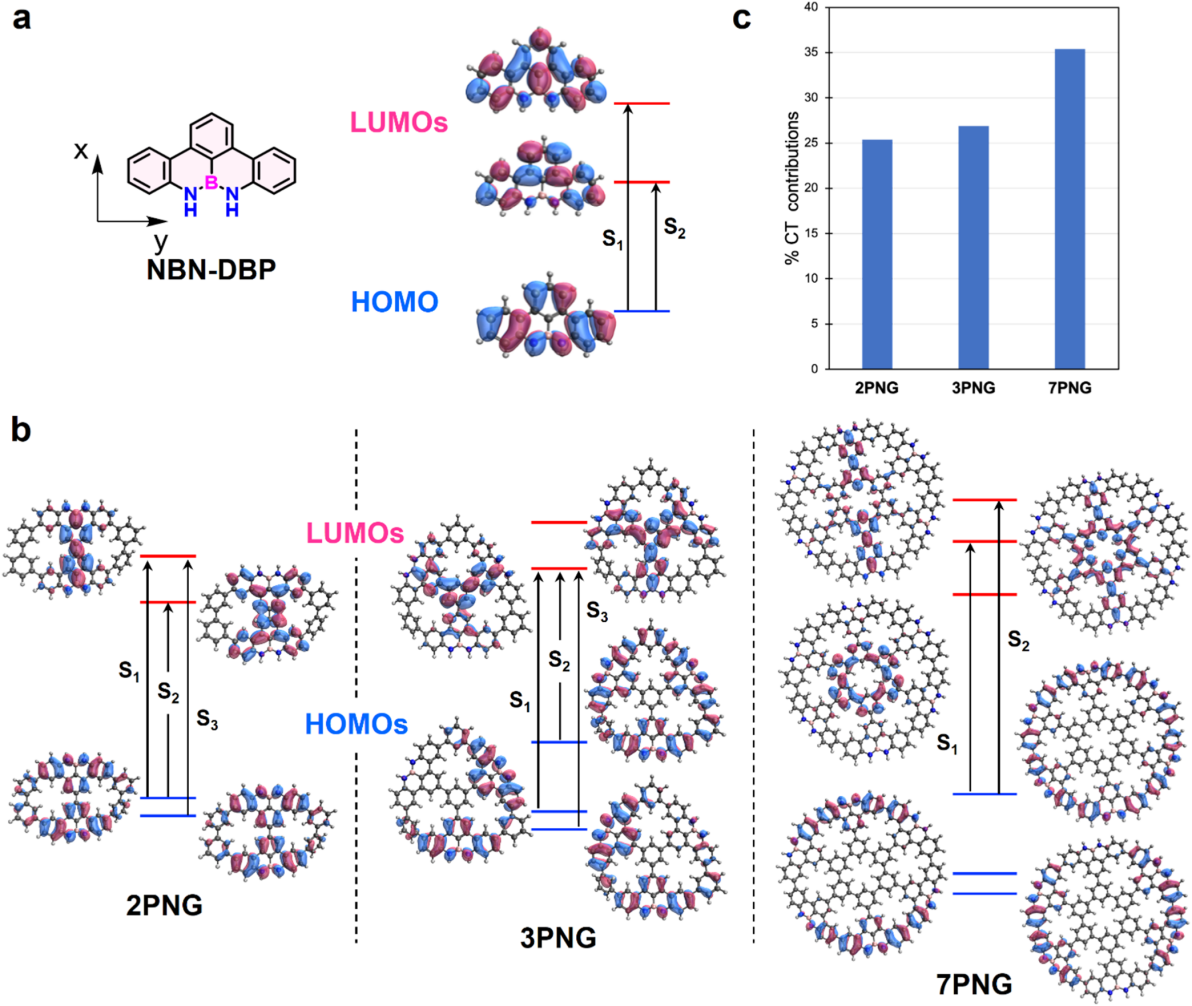
Electronic structure from quantum chemical calculations. (a, b), Electronic structure of NBN-DBP and three molecules. FMOs and low-lying singlet states of NBN-DBP (a), FMOs and low-lying singlet states of **2PNG**, **3PNG**, and **7PNG** (b). Vertical arrows indicate the main contributions (largest linear response amplitudes) to the low-lying singlet–singlet excitations. (c) Charge transfer in S_1_ of three molecules. CT contributions (in %) to the S_1_ excitation in **2PNG**, **3PNG**, and **7PNG**. Molecular fragments are defined in Figure S22.

#### Theoretical Optical Transitions and the Appearance of CT-like Excitations

The low-lying electronic transitions mainly result from the interaction of local excitons between the NBN-DBP units along the periphery (Figure 3a,b). This is particularly noticeable in **7PNG**, wherein the number of nearly degenerated low-energy excited singlets notably increases due to the presence of six NBN-DBP coupled moieties (Table S3). Moreover, these electronic transitions acquire an important periphery-to-core CT character, as the LUMOs are largely delocalized toward the three central phenyl rings, whereas the HOMO is at the periphery. Deconvolution of the lowest-lying electronic transitions of these molecules in terms of local and interfragment contributions indicates that the weight of CT terms increases with the size of the molecule (Figure 3c and Table S3), indicating extensive mixing of local and CT excitations in all molecules, particularly in **7PNG**.

### Photophysics: Through Porous Periphery-to-Core Energy Transfer and Intersystem Crossing

#### Absorption and Emission Spectra

Figure 4a–d shows the absorption and emission spectra of **2PNG**, **3PNG**, and **7PNG** at 80 K (absorption and emission spectra at 298 K in Figure S23). The lowest energy bands of the absorption spectra of **2PNG** and **3PNG** are very similar in shapes and peak positions (i.e., 370 and 391 nm in **2PNG**, and 374 and 393 nm in **3NPG**, respectively), whereas, for **7PNG**, the shape of the absorption spectrum changes with three lower energy components at 381, 401, and 413 nm, which represent a redshift of approximately 20 nm. The assignment of these bands is not linked to a single transition but results from the contribution of several close in energy S_0_ → S_1_, S_2_, S_3_, S_4_ excitations (Table S3). The stronger bands at around 320–340 nm all belong to high-energy excitation transitions. This redshift is due to an increment of π-delocalization around the NBN triphenyl moieties, as highlighted above in the HOMO of **7PNG**, while this is limited to the consecutive NBN moieties of **2PNG** and **3PNG**.

**Figure 4:**
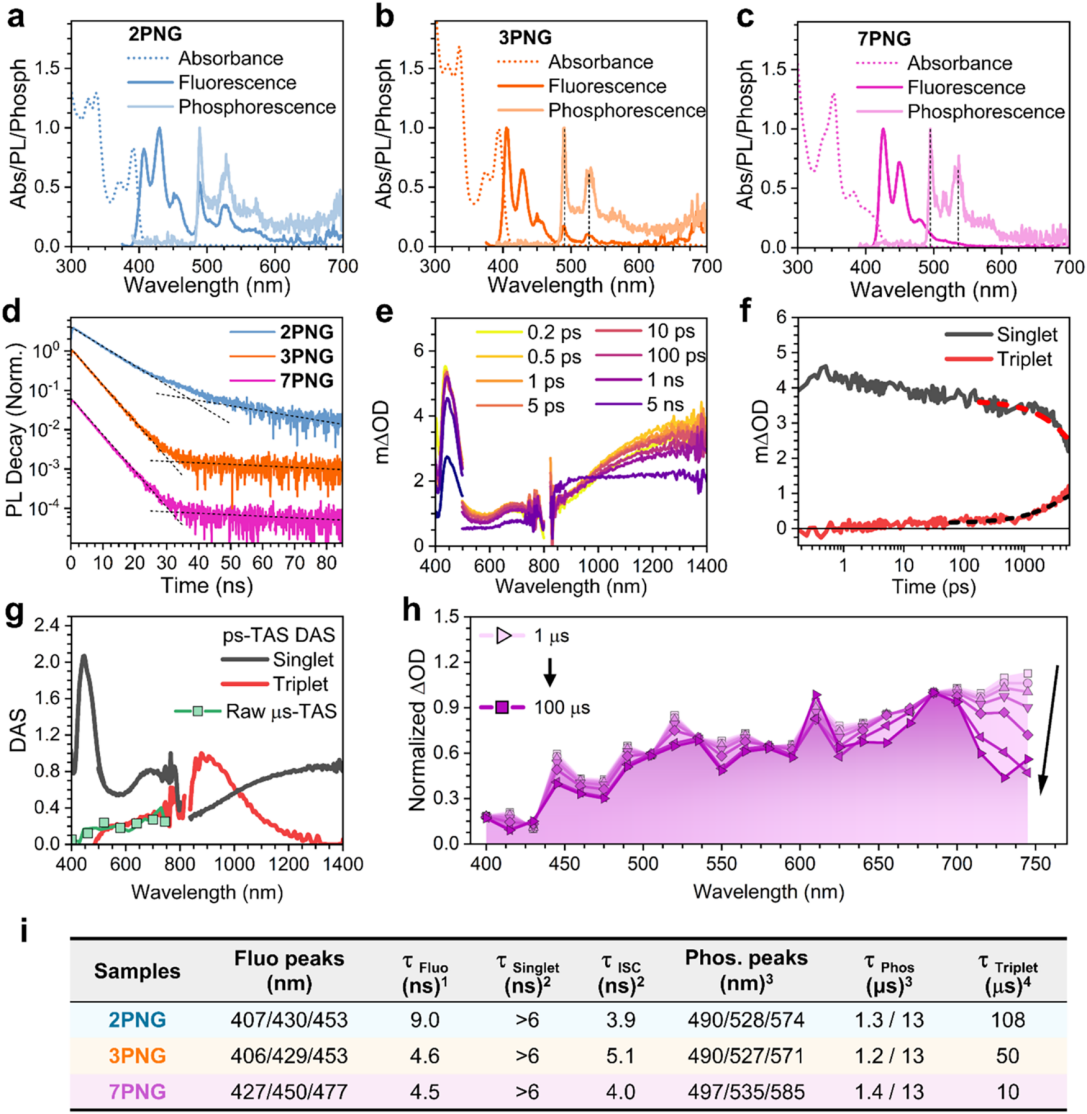
Photophysics. (a–d), Steady-state absorption and emissions and lifetimes. Electronic absorption spectra (dotted lines), photoluminescence spectra (solid darker lines), and phosphorescence spectra (solid lighter lines) were recorded in 2-methyl tetrahydrofuran at 80 K of: **2PNG** (a), **3PNG** (b), and **7PNG** (c) in ∼ 10^–5^ M solutions. (d), Normalized and displaced time evolution of the photoluminescence band at 410–430 nm of the three compounds at 80 K in 2-methyl tetrahydrofuran. (e), fs-TA spectra of **7PNG** at several delays, from 0.2 ps to 5 ns. The data was obtained upon excitation at 355 nm at 0.25 mW. (f), Decays obtained for the two species (singlet and triplet, black and red, respectively) obtained upon global analysis on the fs-TAS of **7PNG**. Exponential fittings have been added as dashed lines. (g), Decay associated spectra (DAS) of the two species obtained by global analysis treatment of fs-TA data. The green spectrum is the TA spectrum obtained by μs-TAS upon 1 μs. (h), Normalized μs-TAS data of **7PNG**. The arrow indicates the difference in lifetimes for the two spectral regions. (i), Photophysical data in solutions of the three compounds. (^1^From steady state single photon counting experiment at 298 K.^2^ From femtosecond to nanosecond transient absorption spectroscopy at 298 K.^3^ From gate delayed measurements at 80 K.^4^ From microsecond transient absorption spectroscopy at 298 K. Fluo: fluorescence; Phos: phosphorescence; ISC: intersystem crossing.).

Emission spectra at 298 K displays a strong band with two well-resolved vibronic peaks (Figures S23 and 4i) that become three at 80 K for **2PNG** (i.e., 407, 430, and 453 nm) and **3PNG**. In **7PNG**, the emission bands are similar to those of the smaller counterparts, again red-shifted, such as in the absorption profiles. In **7PNG**, the absorption and emission spectra do not show mirror-like shapes, indicating some mismatch between absorbing and emitting excited states. Interestingly, in the 80 K emission spectrum of **2PNG**, there are three weak bands at 490, 528, and 574 nm, which are also present in the 80 K spectrum of **3PNG** but are absent in **7PNG**.

#### Emission Kinetics and Phosphorescence

Decay kinetics were obtained for the three molecules in the two different regions: at 410 nm for **2PNG** and **3PNG**, and for **7PNG** at 430 nm (i.e., there is a clear emission peak at 298 K) and 530 nm (i.e., the new emission bands appear at 80 K, Figure S23). Lifetimes probed at 410–430 nm are all in the range of nanoseconds and hence ascribed to fluorescence emission (Figure 4d). On the other hand, lifetimes at 530 nm are on the microsecond time scale for the three molecules at 80 K. This long lifetime aims us to measure their emission spectra on the microsecond time scale using a pulsed lamp with gated delayed detection for the three compounds in solutions at 80 K (Figure S24). These delayed emission spectra are very similar in vibronic structure to the fluorescence profiles with redshifts by 0.4–0.5 eV which are typical differences between lowest energy singlet and triplet excited states energies what together with μs lifetimes prompted us to assig the latter to triplet luminescence (i.e., phosphorescence). Furthermore, fluorescence and phosphorescence do not take place independently. Hence, a careful analysis of the kinetics of the emission at 80 K at 410 nm of **2PNG** (Figure S24) without phosphorescence signal indicates a biexponential decay with a lifetime component of 9 ns (i.e., prompt fluorescence) and a second contribution with a lifetime of ∼ 0.03 μs. This reveals that, at the energy band of the fluorescence emission, there are fast and delayed emitted photons.

#### Transient Absorption Spectroscopy

Femtosecond transient absorption spectroscopy (fs-TAS) characterization has been carried out to address the excited state dynamics of these molecules (**7PNG** in Figure 4e, **2PNG** and **3PNG** in Figure S25). A similar positive multiband pattern of excited state absorbance bands (ESA) at ca. 425, 625, and 1200 nm for the three molecules is observed. This ESA appears right upon excitation and shows negligible time evolution in the picosecond time scale. The disappearance of this signature is concomitant to the rise of a new band that remains constant over the nanosecond time detection limit of the technique. Global analysis (GA) was performed to elucidate the position and nature of each of these bands (**7PNG** in Figure 4f,g, **2PNG** and **3PNG** in Figure S26). The GA data shows the existence of two decay associated spectra (DAS) of two different species: (i) one with the shape of the ESA seen at the first picoseconds in Figure 4f and with a lifetime that extends into the nanoseconds ascribed to a singlet excited state species; and (ii) another species with a single ESA band with maxima approximately at 700, 690, and 890 nm for **2PNG**, **3PNG**, and **7PNG**, respectively.

To get insight into the nature of this second species, we rely on microsecond TAS (μs-TAS) characterization, indicating ESA bands with lifetimes of 100, 50, and 10 μs for **2PNG**, **3PNG**, and **7PNG**, respectively (Figure S27). The μs-TAS spectra unambiguously matches the DAS obtained through global analysis (Figure 4h for **7PNG**, and Figure S28 for **2PNG** and **3PNG**). Furthermore, these μs ESA bands appear/disappear reversibly with the absence/presence of oxygen, indicating their triplet species nature (Figure S29). Hence, the evolution dynamics of GA analysis (Figures 4g and 26) reveals a singlet species decaying within 3–4 ns, coinciding with the emergence of a triplet species signal, also within nanoseconds, via ISC. Noticeably, this ISC rate is very similar to the actual fluorescence rate (also in the range of the 4–6 ns lifetime, Figure S24). While a direct singlet-to-triplet conversion via ISC is evident, the dynamics of the resulting triplets are not uniform, as exemplified by the uneven time evolution observed in **7PNG** (Figure 4h). The μs-TAS spectra of **7PNG** shows that over 675 nm there is a faster triplet component (also sensitive to the presence of O_2_, Figure S29) that extends into the NIR and disappears in a few μs, leading to a final spectral component that decays following a similar evolution and lifetime as those of **2PNG** and **3PNG**. The existence of a fast triplet decay with ESA bands at smaller energies reveals the excitation dynamics within the high-energy triplet manifold of states (T_n_). In consequence, the μs-TAS study of **7PNG** reveals the dynamics of multiple triplet excited states with different rates of disappearance.

### Discussion of Photophysics and Electronic Structure

Thermally activated delayed fluorescence (i.e., TADF) is a well-known photophysical event taking place due to near equalization of excited singlet and triplet energy levels, allowing prompt and delayed fluorescence mediated by the existence of efficient direct and reversed ISC. There are three main groups of TADF emitters, each employing molecular design approaches to narrow or eliminate singlet–triplet energy gaps (Δ*E*_ST_). These approaches focus on reducing the HOMO–LUMO exchange interaction by minimizing the overlap between the two frontier orbitals and include: (i) arranging donor–acceptor moieties to produce low-lying singlet and triplet CT states,^51^ (ii) positioning the HOMO and LUMO in orthogonal moieties,^52^ and (iii) utilizing polycyclic frameworks with disjointed FMOs, achieved through the opposite resonance effect of electron-rich and electron-deficient atoms, known as multiresonant TADF compounds.^53^

ISC in NBN-doped π-conjugated molecules have been reported in the literature, in which spin–orbit coupling (SOC) is typically activated by the presence of CT states promoted by the NBN moieties. Photoluminescence emission measurements were conducted on **2PNG**, **3PNG**, and **7PNG** species using various solvents (Figure S30). These measurements revealed a red-shift and spectral broadening in solvents of increasing polarity, providing confirmation of the involvement of CT states. Microsecond time-resolved transient absorption in Figure 4h reveals the T_1_ → T_n_ transitions, characterized by broad bands, further indicating their CT characters. These results agree with our computational characterization of the low-lying excitations holding important CT contributions. Moreover, the simulation of excited state relaxation results in the localization of the excitation on one of the NBN-DBP units associated with a loss of the molecular symmetry (Figure 5a) or excited state symmetry breaking, which explains the similarities of the fluorescence and phosphorescence spectra of the three molecules.

**Figure 5:**
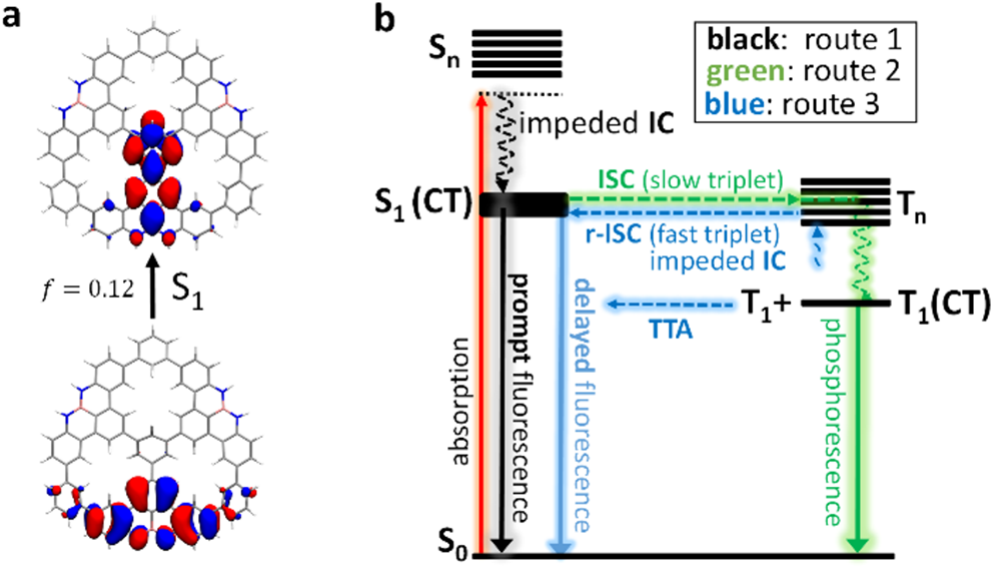
Electronic structure and photophysics. (a), Localized S_1_ state. Natural transition orbital pair (hole and electron) to the lowest excited singlet of **3PNG** at the T_1_ optimized geometry (*f* = oscillator strength). (b), Simplified Jablonski diagram highlighting the main processes after absorption and relaxation on S_1_. Route 1: fast/prompt fluorescence decay; route 2: ISC/phosphorescence decay; and route 3: r-ISC/TTA/delayed fluorescence decay.

The energy gap of S_1_ to the lowest triplet states is rather large for the three molecules (computed between 0.65 and 0.98 eV, Tables S3–S6), which seems to prevent direct S_1_ → T_1_ ISC. On the other hand, there are multiple higher-lying triplets (T_n_ in Figure 5b) nearly degenerated to the lowest singlets with nonzero SOC constants. As the size of the molecule increases, i.e., from **2PNG** to **3PNG** to **7PNG**, the density of accessible T_n_ states at the S_1_ energy increases notably (Tables S4–S6), resulting in multiple available pathways for triplet generation. We argue that these two elements, sizable SOCs and a large density of states at the singlet excitation energy region, are responsible for the efficient transition into the triplet manifold via ISC in **2PNG**, **3PNG**, and **7PNG**. On the other hand, the existence of distinct dynamics within the triplet excited state manifold, as depicted in Figure 4h for **7PNG**, suggests a significant energy difference between the high-energy lying triplets (T_n_) and T_1_. This gap may slow down internal conversion (IC) relaxation to T_1_, and thus increase the lifetime of the T_n_, such as the two different triplet species lifetimes in Figure 4h suggest. This long-lived T_n_, in comparison with other higher lying triplets, enables reverse ISC (rISC), i.e., T_n_ → S_1_. This pathway, facilitated by favorable energy alignment and nonzero SOC between T_n_ and S_1_, provides a viable channel for singlet repopulation and delayed fluorescence. Therefore, we surmise that rISC back to the singlet manifold takes place from a high-lying triplet state, which implies that rISC must compete with IC to T_1_. In fact, this agrees with the rather different lifetimes measured in solution for the delayed emission (tens of ns range) and phosphorescence (μs range), indicating that these two processes emerge from different spin-triplet species. Nonetheless, the existence of two different triplet kinetics might also suggest the presence of triplet–triplet annihilation (TTA in Figure 5) as an alternative triplet deactivation route to rISC. Based on our experimental analysis and computational characterization, the complex/rich photophysical behavior of the molecule can be illustrated using the Jablonski diagram presented in Figure 5b.

## Conclusions

In summary, we reported the first examples of solution-phase synthesized heteroatom-doped multiporous molecular carbons, where the NBN atoms are located at the zigzag edge of the peripheral framework. X-ray crystallographic analysis and STM study clearly revealed the unique porous structural characteristics of the molecular carbons and their self-assembly behaviors. Experiments and theoretical calculations have shown that these molecular carbons have a dominant localized aromatic character and distinctive energy levels, which may be considered as the well-defined molecular cutout of graphene nanomeshes. More importantly, we created a new strategy of producing minimal wave function overlaps at almost planar geometry by segmenting the electronic structure of nanographenes by insertion of pores, forcing the excitation to occur between the periphery and the core. Steady-state and transient spectroscopy, along with DFT calculations, depicted the existence of an intramolecular energy-transfer process from the peripheral to the core in these NBN-doped molecular systems, in which the peripheral-to-core excitations could promote ISC. This study presents a new approach or structure-spectroscopic relationship where segregation of the peripheral-to-core excitations fueled exciton singlet–triplet transference useful for the design of phosphorescent and potentially TADF emitters. In general, this research allows us to understand and design novel porous nanocarbons and heteroatom-doped graphene nanomeshes, which are expected to exhibit flourishing optoelectronic properties and potential applications in nanophotonic and nanoelectronics.

## Supplementary Material



## Notes

Experimental methods, synthesis; X-ray crystallographic structures analysis; self-assembly of PNGs at the solution-solid interface; electronic structure calculations; photophysical properties; NMR spectra; high-resolution mass spectrometry; and additional supporting figures/tables (PDF)
