# Supplementary material for: Piercing Molecular Graphenes: Precision Synthesis and Photophysics of NBN-Edged Porous Molecular Carbons
Source: J Am Chem Soc. 2025 May 28;147(23):20098–108. doi: 10.1021/jacs.5c06175 (PMC12165452; doi:10.1021/jacs.5c06175)
Supplement: Supplementary file 1 [file ja5c06175_si_001.pdf]

Supporting information for

Piercing Molecular Graphenes: Precision Synthesis and Photophysics of NBN-edged Porous Molecular Carbons

Yang Yu[1,2], Asier Izu[3,4], José M. Marín Beloqui[5], Shammi Rana[6], Kunal S. Mali[6], Steven De Feyter[6], David Casanova[3,7,*], Juan Casado[5,*], Junzhi Liu[1,2,8*]

[1]Department of Chemistry, HKU-CAS Joint Laboratory on New Materials and Shanghai-Hong Kong Joint Laboratory on Chemical Synthesis, The University of Hong Kong, Hong Kong 999077, PR China.
[2]State Key Laboratory of Synthetic Chemistry, The University of Hong Kong, Hong Kong 999077, PR China.
[3]Donostia International Physics Center (DIPC), 20018, Donostia, Euskadi, Spain
[4]Polimero eta Material Aurreratuak: Fisika, Kimika eta Teknologia, Kimika Fakultatea, UPV/EHU, PK 1072, 20018 Donostia, Euskadi, Spain
[5]Department of Physical Chemistry, University of Malaga, Campus de Teations s/n, 229071, Malaga, Spain
[6]Division of Molecular Imaging and Photonics, Department of Chemistry, KU Leuven, Celestijnenlaan 200F, 3001 Leuven, Belgium
[7]IKERBASQUE, Basque Foundation for Science, 48009 Bilbao, Euskadi, Spain.
[8]Materials Innovation Institute for Life Sciences and Energy (MILES), HKU-SIRI, Shenzhen 518048, PR China.
Email: david.casanova@dipc.org; casado@uma.es; juliu@hku.hk

Table of Contents

1. Experimental methods ···· 3

2. Synthesis ···· 6

    2.1. Synthesis of **2PNG** ···· 6

    2.2. Synthesis of **3PNG** ···· 12

    2.3. Synthesis of **7PNG** ···· 16

3. X-ray crystallographic structures analysis ···· 21

4. Self-assembly of PNGs at the solution-solid interface ···· 32

5. Electronic structure calculations ···· 36

6. Photophysical properties ···· 41

7. NMR spectra ···· 48

8. High-resolution mass spectrometry ···· 65

9. References ···· 74

1. Experimental methods

Materials:

All the solvents and reagents were purchased from commercial suppliers and used without further purification unless noted otherwise. Compounds **9**[1], **13**[2], and **17**[3] were prepared using literature methods. The details of the synthesis are described in the synthesis section.

Characterization:

The spectra of ^{1}H NMR (400, 500 or 600 MHz) and ^{13}C NMR (101 or 126 MHz) were acquired on the Avance DRX Bruker 400, 500 or 600 MHz FTNMR Spectrometer. Chemical shifts (δ) are reported in ppm. High-resolution mass spectra were performed on a Bruker Q-Tof Maxis II mass spectrometer, DFS high-resolution magnetic sector mass spectrometer, and Bruker Autoflex Speed MALDI-TOF-MS.

Spectroscopic characterization:

The absorbance and emission characteristics were assessed in 2-methyl tetrahydrofuran (2-MeTHF) solutions across the 300-80 K temperature range utilizing an Oxford Instruments cryostat OPTISTAT. Emission analysis was conducted using an Edinburgh Analytical Instrument spectrofluorometer (FLS920P) equipped with a 400 mW pulsed xenon flash-lamp (Xe900). PL lifetimes were characterized by using ultrafast detector F-G05 with the use of pulsed lasers as excitation sources. Microsecond transient absorption spectroscopy was performed on freshly prepared solutions of approximately 10^{-5} M in 2-MeTHF at room temperature using a laser flash photolysis system from Luzchem, employing a pulsed Nd:YAG laser with a 355 nm as excitation wavelength. The probe light source was a Lo255 Oriel xenon lamp. The experimental setup also comprised a 77200 Oriel monochromator, an Oriel photomultiplier (PMT) system, and a TDS-640A Tektronix oscilloscope. Individual energy pulses were approximately 15 mJ. Femtosecond transient absorption spectroscopy was carried out using a Helios system from Ultrafast Systems, incorporating an amplified femtosecond Spectra-Physics Solstice-100F laser with a pulse width of 128 fs and a repetition rate of 1 KHz, coupled with a Spectra-Physics TOPAS Prime F optical parametric amplifier spanning 195-22000 nm. Samples were purged with nitrogen for conventional measurements. Oxygen was purged through the solutions to perform oxygen sensitivity experiments in μs-TAS.

Scanning tunneling microscopy:

The solvent used for STM experiments, namely, 1-phenyloctane (99%, Sigma-Aldrich) was used without further purification. The PNGs were dissolved in appropriate amounts in 2 mL of 1-phenyloctane. The solids were dissolved using gentle sonication in an ultrasonic bath which resulted in clear solutions for all PNGs studied. The STM experiments were carried out by adding a drop (~ 7-8 μL) of the PNG solution to the freshly cleaved surface of highly oriented pyrolytic graphite (HOPG, grade ZYB, Advanced Ceramics Inc., Cleveland, USA). The STM experiments were performed at room temperature (21-23 °C) using a PicoSPM (Keysight Technologies) machine operating in constant-current mode with the tip immersed in the supernatant liquid. STM tips were prepared by mechanically cutting a Pt/Ir wire (80%/20%, diameter 0.25 mm, Advent Research Materials). Good resolution STM images for the PNGs were obtained within the voltage window of −1.1 V to −1.5 V and tunneling current range of 30 pA to 100 pA. For analysis purposes, the recording of a monolayer image was followed by imaging the graphite substrate under the same experimental conditions, except for increasing the current and lowering the bias. The images were corrected for drift *via* Scanning Probe Image Processor (SPIP) software (version 6.5.1, Image Metrology ApS), using the recorded graphite images for calibration purposes, allowing a more accurate unit cell determination. The unit cell parameters were determined by examining at least 3 images and only the average values are reported. The images are Gaussian filtered. The imaging parameters are indicated in the figure caption: tunneling current (I_{set}), and sample bias (V_{bias}). The molecular models presented next to the STM images were prepared using HyperChem™ (version 8.0.1) was employed. Peripheral alkyl chains were not considered for the sake of simplicity.

Theoretical Calculations:

To predict the geometrical and electronic properties of the molecules, the Gaussian 16 program package was applied using DFT at the level of B3LYP with a 6-311G(2d,p) basis set. The molecular orbitals were visualized in GaussView 6.0. All the geometrical optimization was performed in the gas phase and based on the single crystal structures. Nucleus-independent chemical shifts (NICS) values were calculated using the standard gauge-invariant atomic orbital (GIAO) method at the B3LYP function.[4-6] The 6-311+G(2d,p) basis set was used for the C and H atoms. All NICS values were averaged by two positions (above and below the plane) of each molecule. Excited state calculations have been done with linear response time-dependent DFT (TDDFT) under the Tamm-Dancoff approximation (TDA), with the M06-2X[7] exchange-correlation functional and the 631G(d) basis set. Spin-orbit couplings between excited singlet and

triplet states have been computed at the same level considering one-electron contributions of the Breit–Pauli Hamiltonian.[8] These calculations have been performed with the Q-Chem program.[9] Analysis and quantification of the CT character of singlet-singlet excitations has been done with by means of the transition density matrix with the Theodore package.[10] Molecular fragments employed in this analysis are defined in **Figure S22**.

2. Synthesis

2.1. Synthesis of 2PNG

*Synthesis of compound **2c***

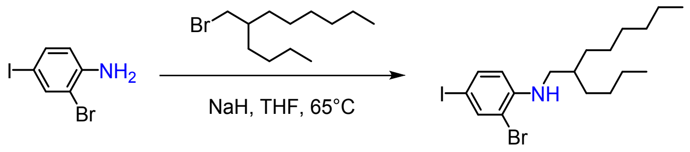

1c **2c**

To a solution of 2-bromo-4-iodoaniline (1.00 g, 3.36 mmol) in THF (33 mL) under nitrogen was added NaH (60% in oil, 242 mg, 6.04 mmol), and the mixed solution was stirred at room temperature for 30 minutes. The solution was refluxed for 2 h. After cooling to room temperature, 5-(bromomethyl)undecane (1.00 g, 4.03 mmol) was added. The solution was refluxed for 12 h. The resulting mixture was cooled to room temperature, poured into water, and extracted with ethyl acetate several times. The organic phase was dried over $MgSO_4$, and the solvent was evaporated in *vacuo*. The product was purified by chromatography on silica gel to give compound **2c** as a colorless oil (0.95 g, 60%). ^{1}H NMR (400 MHz, $CDCl_3$): δ ppm 7.66 (d, J = 2.0 Hz, 1H), 7.40 (dd, J = 8.6, 2.0 Hz, 1H), 6.37 (d, J = 8.6 Hz, 1H), 4.36 (s, 1H), 3.02 (dd, J = 6.5, 2.8 Hz, 2H), 1.65 (p, J = 6.0 Hz, 1H), 1.33 – 1.25 (m, 16H), 0.90 (dd, J = 7.4, 1.8 Hz, 6H). ^{13}C NMR (101 MHz, $CDCl_3$): δ ppm 145.1, 139.7, 137.1, 113.0, 110.5, 75.8, 47.4, 39.9, 39.6, 37.4, 32.7, 32.4, 32.3, 32.0, 29.8, 29.1, 28.9, 26.8, 23.2, 23.0, 22.8, 14.3. HRMS analysis (ESI, *m/z*) $[M+H]^+$ calcd for $C_{18}H_{29}BrINH^+$ 466.0601; Found 466.0601.

Synthesis of compound 7

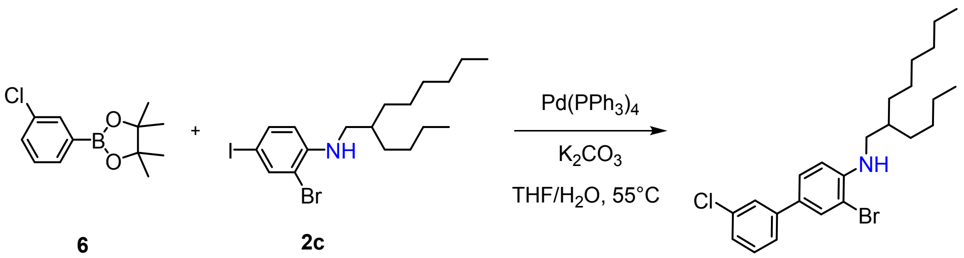

6 **2c** **7**

In a 100 mL long-necked Schlenk flask, 2-bromo-N-(2-butyloctyl)-4-iodoaniline (**2c**) (1.00 g, 2.14 mmol), compound **6** (511.57 mg, 2.14 mmol), K_2CO_3 (1.48 g, 10.72 mmol)

and $Pd(PPh_3)_4$ (124 mg, 0.11 mmol) were charged under the protection of nitrogen. After adding 21 mL degassed THF and H_2O (3:1), the mixture was heated to 55 °C and stirred for 48 hours. After cooling to room temperature, the reactant was poured into brine and extracted by EA three times. The organic phase was dried over magnesium sulfate, and the solvent was evaporated in *vacuo*. The crude product was purified by flash chromatography on silica gel (hexane) to give the product as a pale-yellow oil (531 mg, 55 %). ^{1}H NMR (400 MHz, $CDCl_3$): δ ppm 7.67 (d, J = 2.1 Hz, 1H), 7.49 (d, J = 2.1 Hz, 1H), 7.43 – 7.36 (m, 2H), 7.31 (t, J = 7.8 Hz, 1H), 7.26 – 7.20 (m, 1H), 6.68 (d, J = 8.5 Hz, 1H), 4.45 (t, J = 5.3 Hz, 1H), 3.11 (t, J = 5.6 Hz, 2H), 1.70 (p, J = 6.0 Hz, 1H), 1.41 – 1.27 (m, 16H), 0.91 (dt, J = 10.8, 6.6 Hz, 6H). ^{13}C NMR (101 MHz, $CDCl_3$): δ ppm 145.1, 142.0, 134.8, 130.8, 130.1, 128.9, 127.2, 126.5, 126.4, 124.4, 111.3, 110.2, 47.5, 37.5, 32.3, 32.0, 29.8, 29.1, 26.8, 23.2, 22.8, 14.3. HRMS analysis (ESI, *m/z*) $[M+H]^+$ calcd for $C_{24}H_{33}BrClNH^+$ 452.1537; Found 452.1555.

*Synthesis of compound **8***

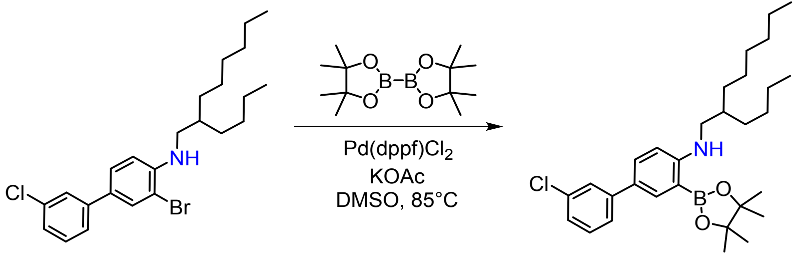

7 **8**

In a 100 mL Schlenk flask, compound **7** (1.20 g, 2.66 mmol), bis(pinacolato)diboron (1.08 g, 4.26 mmol), [1,1′-bis(diphenylphosphino)ferrocene]dichloropalladium(II) (108 mg, 133 μmol), and potassium acetate (0.78 g, 7.98 mmol) were added. The flask was evacuated and charged with nitrogen atmosphere over three times, after which DMSO (13 mL) was injected into the flask through a syringe. The mixture was heated up to 85 °C and stirred for 12 hours. After the mixture was cooled to room temperature, then poured into water (100 mL). Then, the mixture was added to dichloromethane (50 mL), the layers were separated, and the aqueous layer was thoroughly extracted with dichloromethane. The combined organic layers were washed with water and brine and dried over magnesium sulfate. After filtration and evaporation of the solvent, the remaining crude products were purified by silica gel column chromatography using hexane/DCM (10:1) as the eluent to afford white oil (1.26 g, 95 %). ^{1}H NMR (400 MHz, $CDCl_3$): δ ppm 7.87 (d, J = 2.4 Hz, 1H), 7.58 – 7.52 (m, 2H), 7.45 (dt, J = 7.8,

1.4 Hz, 1H), 7.29 (t, $J = 7.8$ Hz, 1H), 7.19 (dt, $J = 8.1$, 1.4 Hz, 1H), 6.62 (d, $J = 8.6$ Hz, 1H), 6.09 (t, $J = 4.9$ Hz, 1H), 3.09 (t, $J = 4.9$ Hz, 2H), 1.69 (q, $J = 6.0$ Hz, 1H), 1.43 – 1.29 (m, 29H), 0.92 (dt, $J = 11.2$, 6.6 Hz, 6H). ^{13}C NMR (101 MHz, $CDCl_3$): δ ppm 154.8, 143.3, 135.6, 134.4, 131.7, 129.7, 126.1, 126.1, 125.6, 124.3, 109.6, 83.6, 46.3, 37.6, 32.4, 32.1, 31.9, 29.9, 29.1, 26.9, 25.0, 23.2, 22.7, 14.2, 14.2. HRMS analysis (ESI, *m/z*) $[M+H]^+$ calcd for $C_{30}H_{45}BClNO_2H^+$ 498.3310; Found 498.3314.

*Synthesis of compound **10***

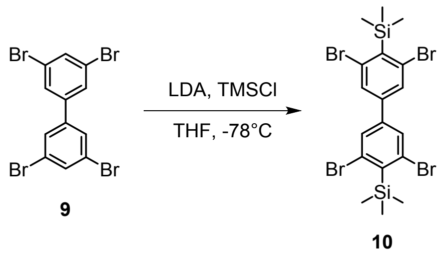

To a solution of 3,3',5,5'-tetrabromo-1,1'-biphenyl (compound **9**, 1.00 g, 2.13 mmol) and trimethylsilyl chloride (508.75 g, 4.68 mmol) in dry THF (40 mL) was added dropwise lithium diisopropylamide (2.0 M in THF, 2.34 mL, 4.68 mmol) at -78 °C under nitrogen. The resulting solution was stirred at -78 °C for one hour and then allowed to come to room temperature. The reactant was hydrolyzed with the dilute sulfuric acid solution. The yellow organic phase was separated, and the water phase was extracted with EA three times. Evaporation of the combined organic solutions left the crude product as a pale-yellow solid residue. It was filtered, washed with cold methanol, and dried to give **10** as a white crystalline material (1.30 g, 99%). ^{1}H NMR (400 MHz, $CDCl_3$): δ ppm 7.67 (s, 4H), 0.58 (s, 18H). ^{13}C NMR (101 MHz, $CDCl_3$): δ ppm δ 140.9, 140.6, 131.5, 131.2, 3.9. MS analysis (APCI, *m/z*) $[M+2H]^+$ calcd for $C_{18}H_{22}Br_4Si_2H_2$ 612.8223; Found 612.7903.

*Synthesis of compound **11***

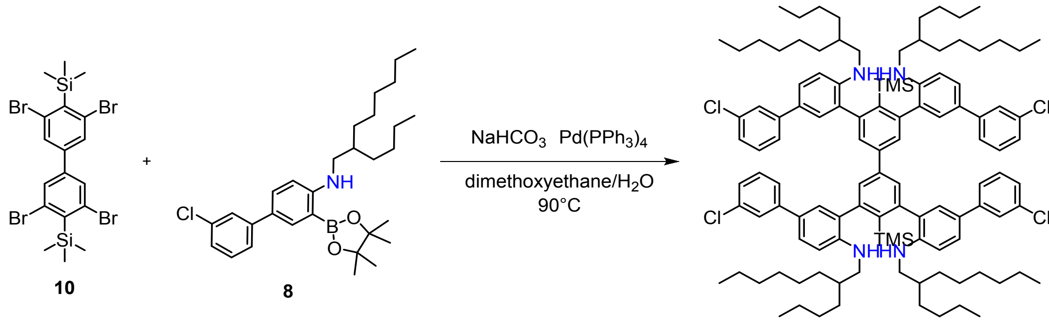

In a 100 mL long-necked Schlenk flask, compound **10** (350 mg, 0.57 mmol) and compound **8** (1.70 g, 3.42 mmol) were charged under the protection of nitrogen. After adding 20 mL dimethoxyethane and 4.60 mL $NaHCO_3$ aqueous solution (2.00 mol/L), the mixture was degassed for 30 min. $Pd(PPh_3)_4$ (132 mg, 0.11 mmol) was added, then the mixture was heated to 90 °C and stirred for 24 hours. The reactant was poured into the brine and extracted by DCM three times. The organic phase was dried over magnesium sulfate, and the solvent was evaporated in *vacuo*. The crude product was purified by flash chromatography on silica gel (hexane/DCM = 8/1) to give the crude product as a white powder (774 mg **mixture**, 77%). Compound **11** was found to be a mixture of conformational isomers, which were inseparable and used without further separation and subjected to the next step directly. HRMS (Maldi-tof-ms) *m/z* $[M]^+$ Calcd for $C_{114}H_{154}Cl_4N_4Si_2^+$ 1778.0478; Found 1778.0675.

*Synthesis of compound **12***

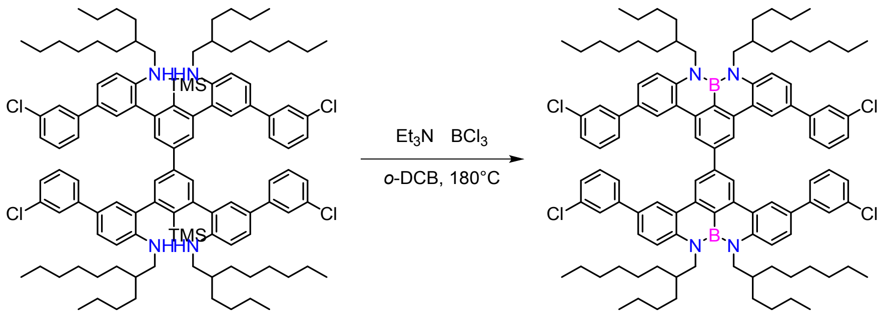

Inside the glove box, compound **12** (50.00 mg, 28.11 μmol), triethylamine (39.83 mg, 394 μmol), boron trichloride (1 M in hexane, 169 μL, 169 μmol), and 1 mL *o*-dichlorobenzene were added to a 10 mL Schlenk tube. The reaction mixture was heated

to 180 °C for 8 hours. After cooling to room temperature, the solvent was evaporated through reduced pressure distillation at 60 °C. The residue was then purified by chromatography on silica gel (hexane / DCM = 10/1) to give compound **12** as a white powder (32.70 mg, 71%). ^{1}H NMR (500 MHz, CD_2Cl_2): δ ppm 8.67 (s, 4H), 8.65 (s, 4H), 7.72 – 7.68 (m, 8H), 7.63 (d, J = 7.8 Hz, 4H), 7.58 (d, J = 8.8 Hz, 4H), 7.34 (t, J = 7.8 Hz, 4H), 7.26 (d, J = 8.8 Hz, 4H), 4.26 (s, 4H), 3.82 (s, 4H), 2.03 (s, 5H), 1.39 (d, J = 33.8 Hz, 32H), 1.04 – 0.84 (m, 32H), 0.65 – 0.25 (m, 24H). ^{13}C NMR (151 MHz, CD_2Cl_2): δ ppm 145.9, 143.7, 143.2, 138.8, 135.0, 131.7, 130.6, 127.4, 127.3, 127.0, 125.8, 125.6, 124.0, 120.2, 118.9, 52.0, 35.1, 32.2, 32.0, 30.3, 29.9, 27.6, 27.1, 24.6, 23.7, 23.1, 14.4. HRMS (Maldi-tof-ms) *m/z* $[M]^+$ Calcd for $C_{108}H_{132}B_2Cl_4N_4^+$ 1648.9409; Found 1648.9345.

*Synthesis of **2PNG***

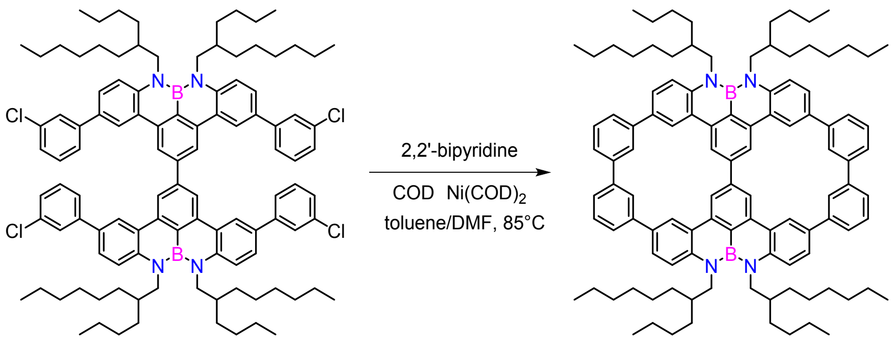

In a 500 mL Schlenk tube, a mixture of 2,2'-bipyridine (0.61 g, 5.64 mmol), 1,5-cyclooctadiene (0.69 mL, 5.64 mmol), and bis(1,5-cyclooctadiene)nickel(0) (1.55 g, 5.64 mmol) in a mixture of toluene (40 mL) and DMF (20 mL) was stirred at 85 °C for 1 h. The mixture was dropwise added a solution of compound **12** (155 mg, 93.96 μmol) in dry degassed toluene (60 mL) over 1 hour, and the reaction mixture was stirred at 85 °C for an additional one day. The mixture was then cooled to room temperature and further stirred for an additional 4 h after adding a saturated aqueous solution of NH_4Cl. The aqueous layer was extracted with toluene (20 mL × 3). The combined organic layer was washed with brine, dried over anhydrous Na_2SO_4, and immediately passed a shot Al_2O_3 flash column, then concentrated in *vacuo*. The crude material was purified by silica gel column chromatography (hexane/DCM = 8/1) to afford the crude target product as a white powder (54.60 mg, 39%). ^{1}H NMR (600 MHz, Toluene-d_8): δ ppm 9.14 (s, 4H), 9.05 (s, 4H), 8.29 (s, 4H), 7.92 (dd, J = 8.5, 2.2 Hz, 4H), 7.81 (d, J = 7.5 Hz, 4H), 7.67 – 7.61 (m, 8H), 7.47 (t, J = 7.5 Hz, 4H), 4.42 (s, 4H), 4.11 (s, 4H), 2.31

(s, 4H), 1.51 – 1.36 (m, 24H), 1.28 – 0.76 (m, 52H), 0.57 (d, J = 63.5 Hz, 12H). ^{13}C NMR (101 MHz, Toluene-d_8): δ ppm 143.3, 142.9, 140.9, 140.4, 139.0, 138.1, 137.6, 132.0, 129.9, 126.8, 126.5, 126.1, 125.1, 119.0, 118.1, 52.5, 35.9, 32.8, 30.8, 30.7, 28.1, 27.5, 25.3, 24.0, 23.5, 14.7. HRMS (Maldi-tof-ms) *m/z* [M]$^+$ Calcd for $C_{108}H_{132}B_2N_4^+$ 1508.0683; Found 1508.0751.

2.2. Synthesis of 3PNG

*Synthesis of compound **3a***

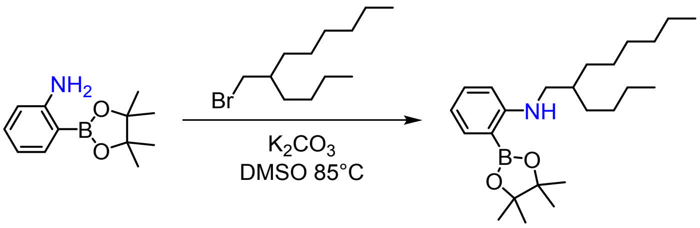

3a

To the stirred mixture of 2-(4,4,5,5-tetramethyl-1,3,2-dioxaborolan-2-yl)aniline (5.00 g, 22.82 mmol) and K_2CO_3 (3.15 g, 22.82 mmol) in 110 mL DMSO, 5-(bromomethyl)undecane (8.53 g, 34.23 mmol) was added and stirring at 85 ℃ until the reaction completion is monitored by TLC plate. The reaction mixture was cooled to room temperature, poured into water, and extracted with EA. The combined extract is washed with 10% brine solution, dried over Na_2SO_4, and concentrated to afford the crude product, which was purified by passing it through a column of silica gel and eluting with hexane/DCM=5/1 to give compound **3a** as pale-yellow oil (5.60 g, 63%). ^{1}H NMR (500 MHz, CD_2Cl_2): δ ppm 7.52 (dd, J = 7.4, 1.8 Hz, 1H), 7.25 (ddd, J = 8.6, 7.2, 1.8 Hz, 1H), 6.54 (d, J = 7.2 Hz, 1H), 6.53 – 6.50 (m, 1H), 5.89 (s, 1H), 3.03 (d, J = 5.8 Hz, 2H), 1.66 (q, J = 6.0 Hz, 1H), 1.45 – 1.36 (m, 6H), 1.34 (s, 12H), 1.33 – 1.27 (m, 10H), 0.90 (dd, J = 12.8, 6.8 Hz, 6H). ^{13}C NMR (126 MHz, CD_2Cl_2): δ ppm 155.7, 137.4, 133.6, 115.3, 109.7, 84.0, 46.9, 38.1, 32.9, 32.6, 32.5, 30.4, 29.7, 27.4, 25.3, 23.8, 23.3, 14.5, 14.5. HRMS analysis (ESI, *m/z*) $[M+H]^+$ calcd for $C_{24}H_{42}BNO_2H^+$ 388.3886; Found 388.3884.

*Synthesis of compound **14***

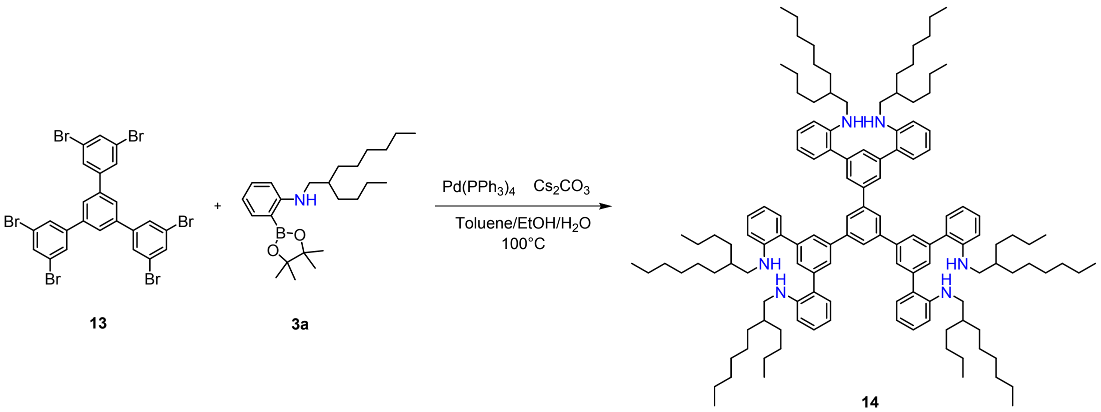

13 **3a** **14**

In a glove box of compound **13** (0.48 g, 618.12 μmol), compound **3a** (2.87 g, 7.42 mmol), Cs_2CO_3 (6.04 g, 18.54 mmol), $Pd(PPh_3)_4$ (214 mg, 185 μmol) were added to a flask. Subsequently, 17 mL of toluene, 4 mL ethanol, and 4 mL H_2O were added in one portion. The reaction mixture was heated at 100°C for 36 h. After cooling to room temperature, the reaction mixture was poured into brine and extracted with ethyl acetate three times. The organic phase was dried over magnesium sulfate, and the solvent was evaporated in *vacuo*. The crude product was purified by column chromatography (hexane/DCM= 4/1) to give compound **14** as an orange oil (1.01 g, 88%). ^{1}H NMR (500 MHz, $CDCl_3$): δ ppm 7.91 (s, 3H), 7.72 (d, J = 1.5 Hz, 6H), 7.53 (s, 3H), 7.23 (td, J = 7.8, 1.7 Hz, 6H), 7.16 (dd, J = 7.5, 1.6 Hz, 6H), 6.74 (t, J = 7.4 Hz, 6H), 6.69 (d, J = 8.2 Hz, 6H), 4.00 (s, 6H), 2.99 (td, J = 10.7, 10.0, 6.0 Hz, 12H), 1.53 (s, 6H), 1.32 (ddt, J = 18.2, 6.8, 3.2 Hz, 18H), 1.18 – 1.07 (m, 78H), 0.81 (t, J = 7.0 Hz, 18H), 0.73 (t, J = 7.2 Hz, 18H). ^{13}C NMR (151 MHz, $CDCl_3$): δ ppm 145.4, 142.2, 142.0, 140.9, 130.1, 129.2, 128.9, 127.1, 126.8, 125.2, 116.5, 110.3, 37.3, 32.2, 31.8, 31.8, 29.6, 28.7, 26.6, 22.9, 22.6, 14.1, 14.0. HRMS (Maldi-tof-ms) *m/z* $[M]^+$ Calcd for $C_{132}H_{192}N_6^+$ 1862.5236; Found 1862.5305.

Synthesis of compound ***15***

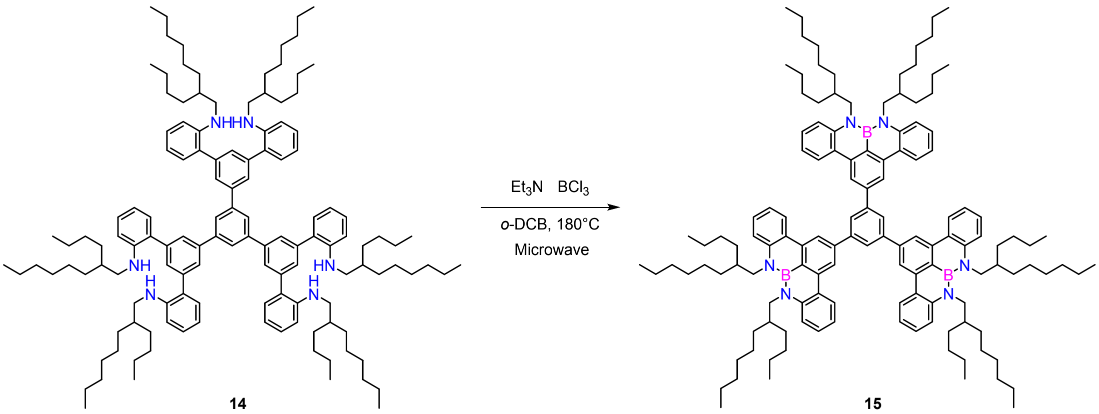

14 **15**

In a glove box of compound **14** (0.12 g, 64 µmol), triethylamine (136.88 mg, 1.35 mmol) and boron trichloride (1 M in hexane, 580 µL, 580 µmol) were added to a microwave vial. Subsequently, 2 mL *o*-dichlorobenzene was added in one portion, and the vial was sealed with a silicon cap and transferred into a microwave reactor. The reaction mixture was heated to 180 °C for 8 hours using the power mode at 300 W. After cooling to room temperature, the solvent was evaporated through reduced pressure distillation at 60 °C. The residue was then purified by chromatography on silica gel (hexane / DCM = 10/1) to give compound **15** as a white powder (30 mg, 26%). ^{1}H NMR (500 MHz, CD_2Cl_2): δ ppm 8.63 (s, 6H), 8.44 (d, J = 8.0 Hz, 6H), 8.38 (s, 3H), 7.46 (d, J = 8.4 Hz, 6H), 7.41 (t, J = 7.6 Hz, 6H), 7.12 (t, J = 7.4 Hz, 6H), 4.19 (s, 6H), 3.75 (s, 6H), 1.97 (s, 6H), 1.10 (d, J = 214.9 Hz, 96H), 0.70 – 0.26 (m, 36H). ^{13}C NMR (151 MHz, CD_2Cl_2): δ ppm 144.5, 143.9, 143.3, 139.1, 128.4, 127.2, 125.5, 125.4, 120.3, 119.0, 118.2, 51.9, 34.9, 32.2, 30.3, 29.9, 27.6, 27.0, 24.7, 23.6, 23.2, 23.1, 14.4. HRMS (Maldi-tof-ms) *m/z* $[M]^+$ Calcd for $C_{132}H_{183}B_3N_6^+$ 1886.4840; Found 1886.4880.

Synthesis of compound ***16***

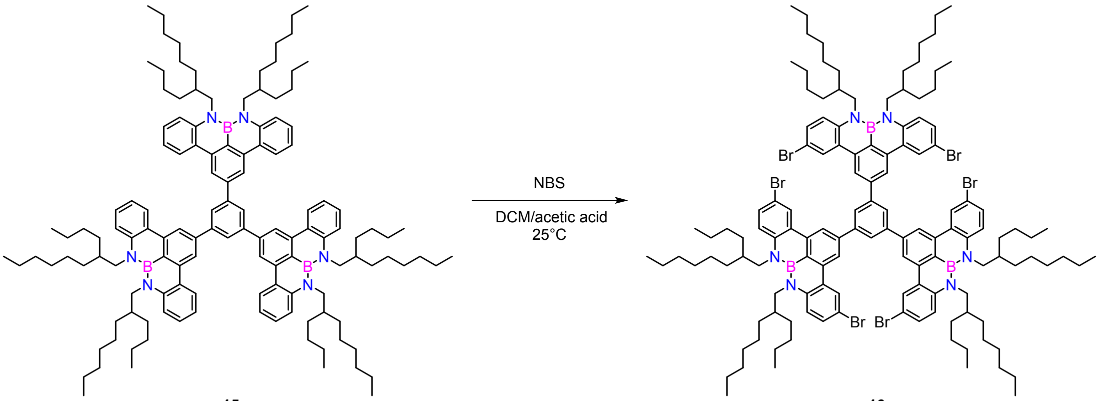

15 **16**

A well-stirred solution of compound **15** (41 mg) in 10 mL DCM and 3 mL acetic acid in a 50 mL flask under a nitrogen atmosphere was added *N*-bromosuccinimide (24 mg in 5 mL DCM) slowly in the absence of light. The resulting mixture was stirred at room temperature for 24 h, and then the reaction was quenched with ice. The aqueous phase was washed with water, extracted with DCM three times, and then dried over magnesium sulfate. The solvent was removed under reduced pressure, and the residue was purified by column chromatography on silica gel (hexane) to give compound **16** a pale-yellow solid (12 mg, 24%). ^{1}H NMR (500 MHz, CD_2Cl_2): δ ppm 8.57 (s, 6H), 8.55 (d, J = 2.3 Hz, 6H), 8.34 (s, 3H), 7.50 (dd, J = 8.9, 2.3 Hz, 6H), 7.34 (d, J = 9.0 Hz, 6H), 4.14 (s, 6H), 3.71 (s, 6H), 1.91 (s, 6H), 1.30 (s, 32H), 0.94 (t, J = 8.5 Hz, 18H), 0.84 (d, J = 6.5 Hz, 32H), 0.65 (s, 32H), 0.37 (s, 18H). ^{13}C NMR (151 MHz, CD_2Cl_2): δ ppm 144.3, 144.2, 142.2, 138.0, 131.1, 128.0, 127.6, 127.4, 120.1, 119.9, 113.3, 52.1, 35.0, 32.5, 30.3, 29.9, 27.6, 23.3, 23.2, 14.5. HRMS (Maldi-tof-ms) *m/z* $[M]^+$ Calcd for $C_{132}H_{177}B_3Br_6N_6^+$ 2359.9415; Found 2359.9426.

Synthesis of ***3PNG***

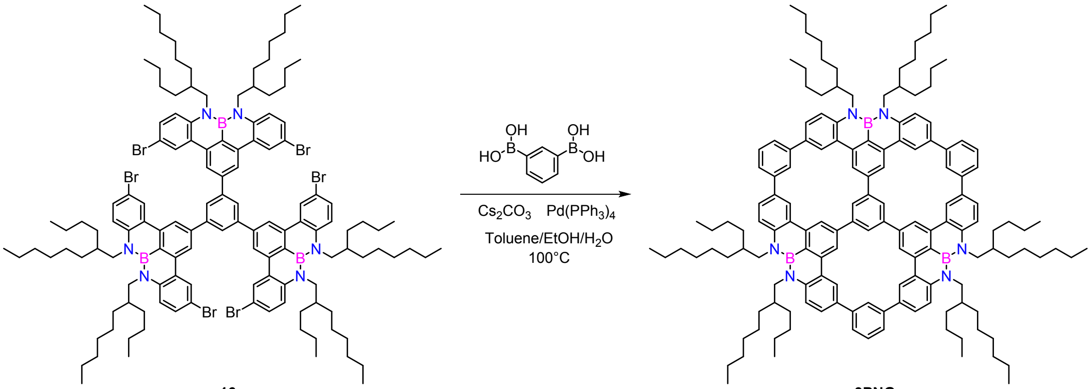

16 **3PNG**

Inside the glove box, compound **16** (50.00 mg, 21.19 μmol), 1,3-benzenediboronic acid (11.55 mg, 69.9 μmol), $Pd(PPh_3)_4$ (7.35 mg, 6.36 μmol, 0.3 equiv), Cs_2CO_3 (414.00 mg, 1.27 mmol, 60 eq), 12 mL toluene, 1.5 mL ethanol and 1.5 mL H_2O were added to 50 mL pressure tube equipped with a stir bar. Then, the mixture was heated to 100 °C and stirred for 36 hours. After the reaction, the mixture was poured into brine and extracted by EA three times. The organic phase was dried over magnesium sulfate, and the solvent was evaporated in *vacuo*; the crude material was purified by chromatography (hexane/DCM=8/1) to give a yellow powder (21 mg, 47% yield). ^1H NMR (500 MHz, CD_2Cl_2): δ ppm 9.51 (s, 9H), 9.34 (s, 6H), 8.84 (s, 3H), 7.99 (dd, J = 8.6, 2.2 Hz, 6H), 7.86 (d, J = 7.6 Hz, 6H), 7.67 – 7.61 (m, 9H), 4.31 (s, 6H), 3.90 (s, 6H), 2.07 (s, 6H), 1.51 – 1.28 (m, 48H), 1.13 – 0.92 (m, 30H), 0.81 – 0.23 (m, 54H). ^{13}C NMR (126 MHz, CD_2Cl_2): δ ppm 142.9, 140.5, 138.7, 131.5, 129.8, 129.5, 128.1, 125.5, 124.4, 123.4, 123.4, 122.9, 121.9, 118.7, 35.7, 31.8, 29.4, 29.3, 27.2, 22.7, 22.5, 13.9, 13.8. HRMS (Maldi-tof-ms) *m/z* $[M]^+$ Calcd for $C_{150}H_{189}B_3N_6^+$ 2108.5313; Found 2108.5377.

2.3. Synthesis of 7PNG

*Synthesis of compound **2b***

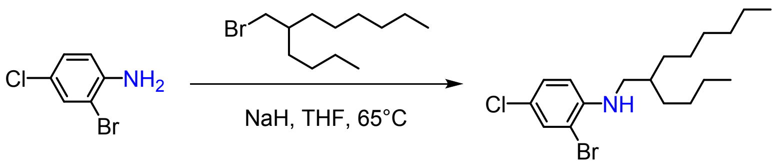

1b **2b**

To a solution of 2-bromo-4-chloroaniline (5.00 g, 24.22 mmol) in THF (240 mL) under nitrogen was added NaH (60% in oil, 1.94 g, 48.43 mmol), and the mixed solution was stirred at room temperature for 30 minutes, then refluxed for 2 h. After cooling to room temperature, 5-(bromomethyl)undecane (7.85 g, 31.48 mmol) was added. The solution was refluxed for 12 hours. The resulting mixture was cooled to room temperature, poured into water, and extracted with ethyl acetate several times. The organic phase was dried over MgSO4, and the solvent was evaporated in *vacuo*. The product was purified by chromatography on silica gel to give compound **2b** as pale-yellow oil (7.10 g, 78%). ^{1}H NMR (500 MHz, CD_2Cl_2): δ ppm 7.40 (d, J = 2.4 Hz, 1H), 7.14 (dd, J = 8.8, 2.5 Hz, 1H), 6.55 (d, J = 8.7 Hz, 1H), 4.34 (t, J = 5.4 Hz, 1H), 3.04 (t, J = 5.9 Hz, 2H), 1.66 (p, J = 6.0 Hz, 1H), 1.38-1.28 (m, 17H), 0.90 (dt, J = 10.2, 6.8 Hz, 6H). ^{13}C NMR (101 MHz, CD_2Cl_2): δ ppm 144.8, 132.1, 128.9, 121.1, 112.1, 109.8, 48.0, 37.9, 32.7, 32.5, 32.4, 30.3, 29.5, 27.3, 23.7, 23.3, 14.5, 14.5. HRMS analysis (ESI, *m*/*z*) $[M+H]^+$ calcd for C18H29BrClNH$^+$ 376.1223; Found 376.1222.

*Synthesis of compound **3b***

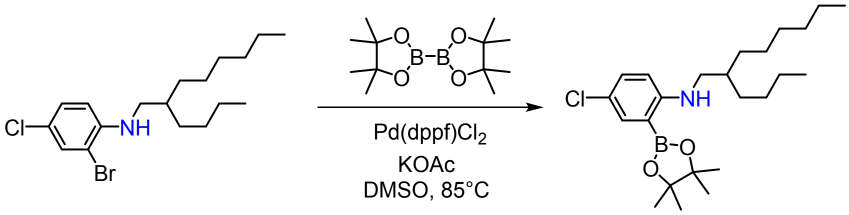

2b **3b**

In a 100 mL long-necked Schlenk flask, compound **2b** (3.37 g, 8.99 mmol), bis(pinacolato)diboron (4.57 g, 17.98 mmol), [1,1′-bis(diphenylphosphino)ferrocene]dichloropalladium(II) (367.15 mg, 449 µmol), and potassium acetate (2.65 g, 26.98 mmol) were added. The flask was evacuated and

charged with nitrogen atmosphere over three times, after which 30 mL DMSO was injected into the flask through a syringe. The mixture was heated up to 85 °C and stirred for 12 hours. After the mixture was cooled to room temperature, then poured into water (100 mL). Then, the mixture was added EA (50 ml), and the layers were separated, and the aqueous layer was thoroughly extracted with EA. The combined organic layers were washed with water and brine and dried over magnesium sulfate. After filtration and evaporation of the solvent, the remaining crude products were purified by silica gel column chromatography using hexane/DCM (8/1) as the eluent to afford compound **3b** as yellow oil (3.33 g, 88%). ^{1}H NMR (500 MHz, CD_2Cl_2): δ ppm 7.48 (d, J = 2.7 Hz, 1H), 7.19 (dd, J = 8.8, 2.7 Hz, 1H), 6.47 (d, J = 8.8 Hz, 1H), 5.91 (d, J = 5.2 Hz, 1H), 3.01 (t, J = 5.4 Hz, 2H), 1.67 (p, J = 6.0 Hz, 1H), 1.40 – 1.30 (m, 28H), 0.91 (dt, J = 12.6, 6.5 Hz, 6H). ^{13}C NMR (126 MHz, CD_2Cl_2): δ ppm 154.2, 136.5, 133.1, 119.8, 111.1, 84.4, 47.1, 38.1, 32.9, 32.6, 32.5, 30.4, 29.7, 27.4, 25.3, 23.8, 23.3, 14.5, 14.5. HRMS analysis (ESI, *m/z*) $[M+H]^+$ calcd for $C_{24}H_{41}BClNO_2H^+$ 422.2996; Found 422.3004.

*Synthesis of compound **4***

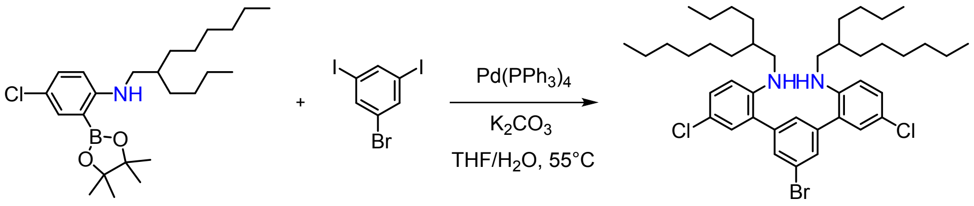

3b **4**

In a 500 mL Schlenk flask, 1-bromo-3,5-diiodobenzene (3.00 g, 7.34 mmol), compound **3b** (9.91 g, 23.48 mmol), K_2CO_3 (6.69 g, 48.43 mmol) and $Pd(PPh_3)_4$ (848 mg, 0.73 mmol) were charged under the protection of nitrogen. After adding 150 mL degassed THF and 50 mL H_2O, the mixture was heated to 55 °C and stirred for 48 hours. After cooling down to room temperature, the reactant was poured into brine and extracted by EA three times. The organic phase was dried over magnesium sulfate and the solvent was evaporated in *vacuo*. The crude product was purified by flash chromatography on silica gel (hexane) to give compound **4** as orange oil (3.40 g, 62 %). ^{1}H NMR (500 MHz, $CDCl_3$): δ ppm 7.58 (s, 2H), 7.37 (s, 1H), 7.18 (dd, J = 8.8, 2.5 Hz, 2H), 7.05 (d, J = 2.5 Hz, 2H), 6.59 (d, J = 8.8 Hz, 2H), 3.83 (s, 2H), 2.96 (d, J = 6.2 Hz, 4H), 1.58 – 1.54 (m, 2H), 1.24 (dq, J = 11.6, 7.5 Hz, 32H), 0.89 – 0.83 (m, 12H). ^{13}C NMR (101 MHz, $CDCl_3$): δ ppm 144.0, 141.3, 131.3, 129.6, 129.2, 128.9, 126.6, 123.6, 121.4,

111.7, 47.6, 37.5, 32.4, 32.1, 32.0, 29.8, 29.0, 26.8, 23.2, 22.8, 14.3, 14.2. HRMS analysis (ESI, *m/z*) $[M+H]^+$ calcd for $C_{42}H_{61}BrCl_2N_2H^+$ 745.3450; Found 745.3457.

*Synthesis of compound **5***

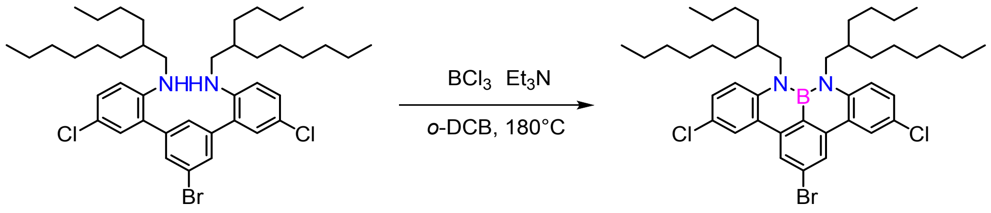

4 **5**

Inside the glove box, compound **4** (389 mg, 522.31 µmol), triethylamine (370 mg, 3.66 mmol), boron trichloride (1 M in hexane, 1.57 mL, 1.57 mmol), and 13 mL *o*-dichlorobenzene were added to a 100 mL Schlenk tube. The reaction mixture was heated to 180 °C for 8 hours. After cooling to room temperature, the solvent was evaporated through reduced pressure distillation at 60 °C. The residue was then purified by chromatography on silica gel (hexane) to give compound **5** as yellow oil (251 mg, 64 %). ^{1}H NMR (500 MHz, CD_2Cl_2): δ ppm 8.26 (s, 2H), 8.16 (d, J = 2.1 Hz, 2H), 7.41 – 7.34 (m, 4H), 4.10 (s, 2H), 3.65 (s, 2H), 1.89 – 1.80 (m, 2H), 1.40 – 1.08 (m, 16H), 0.99 – 0.71 (m, 16H), 0.64 – 0.17 (m, 12H). ^{13}C NMR (101 MHz, CD_2Cl_2): δ ppm 141.8, 139.3, 128.8, 126.5, 125.8, 125.6, 124.9, 123.2, 119.9, 52.1, 34.9, 31.9, 30.4, 30.2, 27.5, 26.9, 24.6, 23.1, 14.3. HRMS analysis (ESI, *m/z*) $[M+H]^+$ calcd for $C_{42}H_{58}BBrCl_2N_2H^+$ 753.3314; Found 753.3297.

*Synthesis of compound **18***

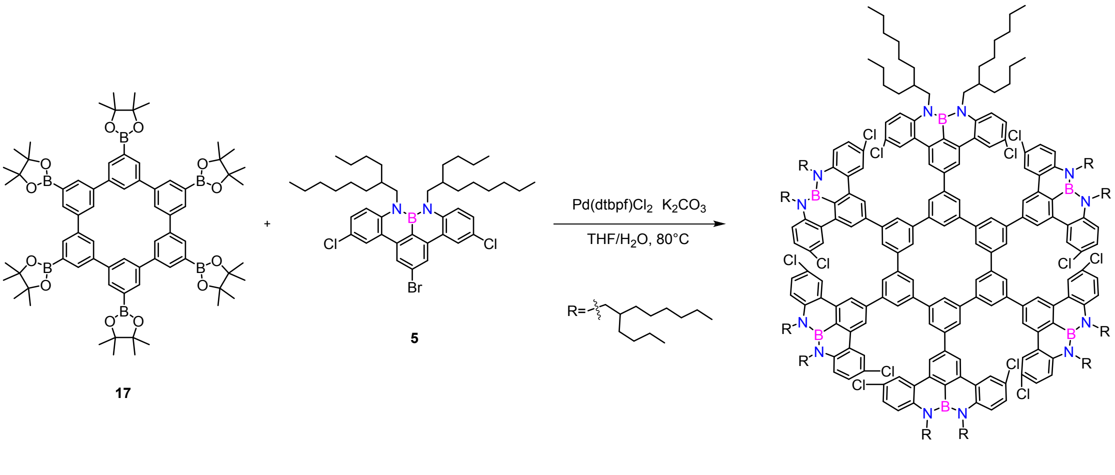

17 **5** **18**

In a 100 mL Schlenk tube, compound **17** (70 mg, 57.7 μmol), compound **5** (521 mg, 692.9 μmol) , potassium carbonate (240 mg) and [1,1'-bis(di-*tert*-butylphosophino)ferrocene]dichloropalladium (Ⅱ) (37.6 mg, 57.7 μmol) were charged under the protection of nitrogen. After adding degassed THF (7 mL) and water (3.5 mL), the mixture was vigorously stirred at 80 °C for 2 days before the addition of water (10 mL). After the extraction with DCM (10 mL×4), the combined organic layer was dried over Na_2SO_4 and concentrated in *vacuo*. The crude material was purified by silica gel column chromatography (hexane/toluene = 4/1) and preparative gel permeation chromatography (GPC) to give a mixture of pale-yellow powder. And then using preparative thin-layer chromatography (PTLC) to purify compound **18** as a pale-yellow solid (42 mg, 16%). ^{1}H NMR (600 MHz, CD_2Cl_2): δ ppm 8.93 (s, 6H), 8.57 (s, 12H), 8.47 (s, 12H), 8.40 (s, 12H), 7.37-7.35 (dd, 12H), 7.33-7.31 (dd, 12H), 4.11 (s, 12H), 3.68 (s, 12H), 1.88 (s, 12H), 1.42–1.27 (m, 96H), 0.85–0.71 (m, 96H). 0.69–0.54 (m, 72H). ^{13}C NMR (151 MHz, CD_2Cl_2) δ 145.0, 144.8, 142.2, 141.7, 138.0, 128.2, 127.1, 126.9, 126.5, 125.8, 125.1, 120.2, 119.6, 52.1, 34.9, 32.5, 32.2, 32.2, 31.7, 30.3, 29.9, 24.5, 23.6, 23.1, 14.4. HRMS (Maldi-tof-ms) *m/z* $[M]^+$ Calcd for $C_{288}H_{366}B_6Cl_{12}N_{12}^+$ 4486.5883; Found 4486.7359.

Synthesis of ***7PNG***

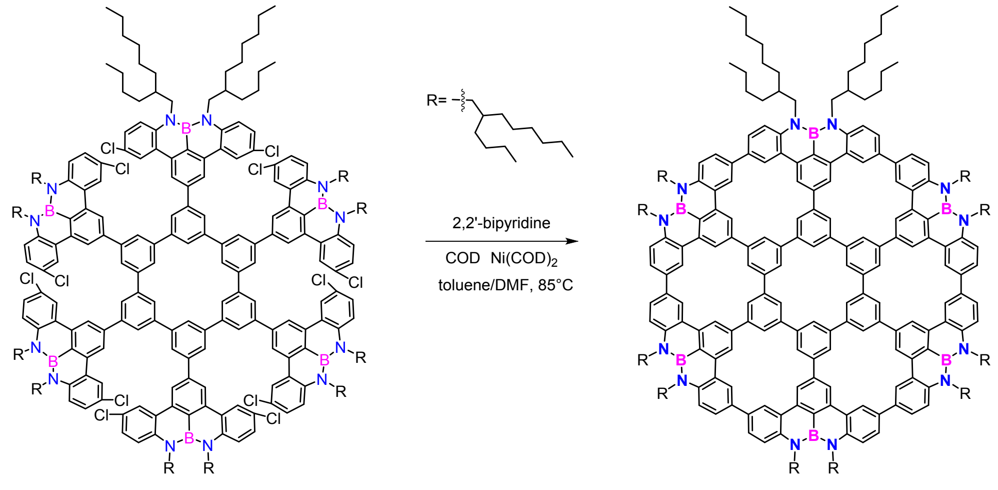

18 **7PNG**

In a 50 mL Schlenk tube, a mixture of 2,2'-bipyridine (2weremg, 1.81 mmol), 1,5-cyclooctadiene (222 μL, 1.81 mmol), and bis(1,5-cyclooctadiene)nickel(0) (496.60 mg, 1.81 mmol) in a mixture of toluene (12 mL) and DMF (4 mL) was stirred at 85 °C for 1 h. The mixture was dropwise added a solution of compound **18** (45.00 mg, 10 μmol, in 6 mL dry and degassed toluene) over 20 mins, and the reaction mixture was stirred at 85 °C for an additional one day. After cooling to room temperature, the reaction mixture was directly filtrated through kieselguhr, and the solvent was evaporated in *vacuo*. The product was purified by column chromatography (hexane/ CH_2Cl_2 = 8/1 to 3/1) to give a crude product as a pale-yellow solid. Then, it is separated by preparative GPC and PTLC to give **7PNG** as a yellow solid (13 mg, 32%). ^{1}H NMR (600 MHz, CD_2Cl_2): δ ppm 9.61 (s, 12H), 9.58 (s, 12H), 9.57 (s, 12H), 9.49 (s, 6H), 8.12 (dd, 12H), 7.65 (dd, 12H), 4.34 (s, 12H), 3.91 (s, 12H), 2.11 (s, 12H), 1.41–1.34 (m, 82H), 1.12 – 0.92 (m, 82H), 0.81 – 0.37 (m, 100H). ^{13}C NMR (151 MHz, CD_2Cl_2) δ 142.9, 140.5, 139.6, 139.2, 130.4, 129.0, 126.1, 124.9, 122.7, 121.6, 121.2, 119.2, 116.7, 52.2, 35.3, 32.3, 30.6, 30.3, 29.9, 27.6, 27.2, 24.9, 23.7, 23.3, 14.4. HRMS (Maldi-tof-ms) *m/z* $[M]^+$ Calcd for $C_{288}H_{366}B_6N_{12}^+$ 4060.9700; Found 4060.9730.

3. X-ray crystallographic structures analysis

The single crystals of **2PNG** and **3PNG** were grown by dilution crystallization. Specifically, tetrahydrofuran and isopropanol were chosen as the good and poor solvents, respectively. The single crystals were obtained after one month through slow diffusion of isopropanol into tetrahydrofuran. The X-ray crystallographic coordinates for structures reported in this article have been deposited at the Cambridge Crystallographic Data Centre (CCDC) under deposition numbers CCDC 2360641 (for **2PNG**) and 2360639 (for **3PNG**). These data can be obtained free of charge from CCDC via https://www.ccdc.cam.ac.uk/structures/.

Table S1. The X-ray diffraction data of **2PNG** and **3PNG**.

	**2PNG**	**3PNG**
CCDC No.	2360641	2360639
Moiety formula	$C_{108}H_{132}B_2N_4$	$C_{150}H_{189}B_3N_6$
Formula weight	1507.79	2108.49
Temperature, K	172(2)	193(2)
Crystal size, mm^3	0.1×0.08×0.08	0.130×0.110×0.100
Crystal system	triclinic	monoclinic
space group	P-1	P 21
a, Å	13.7576(5)	13.763(3)
b, Å	18.4992(7)	25.401(5)
c, Å	18.5081(7)	19.364(5)
α, deg	107.715(2)	90
β, deg	95.201(2)	94.450(13)
γ, deg	96.633(2)	90
V, $Å^3$	4417.4(3)	6749(3)
Z	2	2
Dcalcd., $g\cdot cm^{-3}$	1.134	1.038

F(000)	1636	2292
Radiation	CuK\a(λ =1.54178)	GaK\a(λ = 1.34139)
μ, mm^{-1}	0.479	0.279
2Θ range for data collection, °	5.08 to 116.74	5.002 to 87.004
Index ranges	-15<h<13, -19<k<21, -21<l<20	-15<h<15, -29<k<29, -22<l<21
No. of collected reflections	13045	32439
no. of unique ref.(R_{int})	3586(0.1642)	7085/0.1243
Data/restraints/parameters	3586/1191/1162	7085/2076/1764
R_1, wR_2 [obs I>2σ (I)]	0.1642/0.3247	0.1243/0.2687
Largest diff. peak/hole, e·$Å^{-3}$	0.378/ -0.292	0.345/ -0.349
Goodness-of-fit on F^2	1.015	1.016

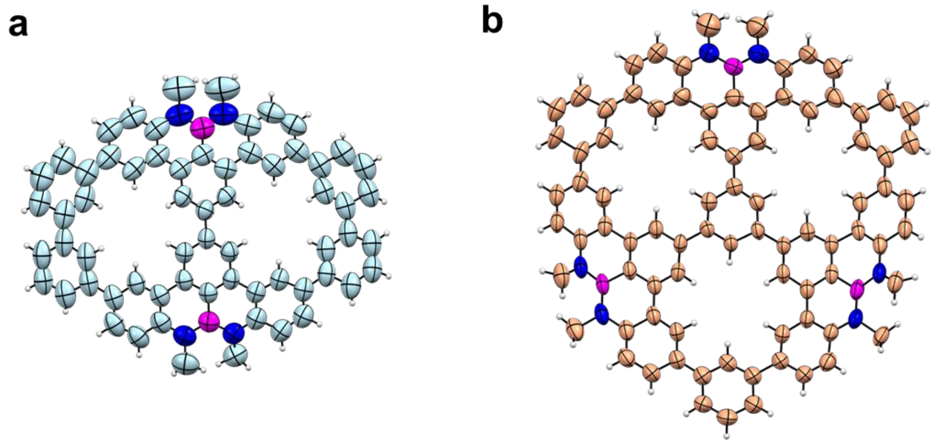

Figure S1. X-ray crystallographic molecular structures. ORTEP diagrams of **2PNG** (**a**) and **3PNG** (**b**). Thermal ellipsoids are shown at the 50 % probability level. Branched alkyl chains are omitted for clarity.

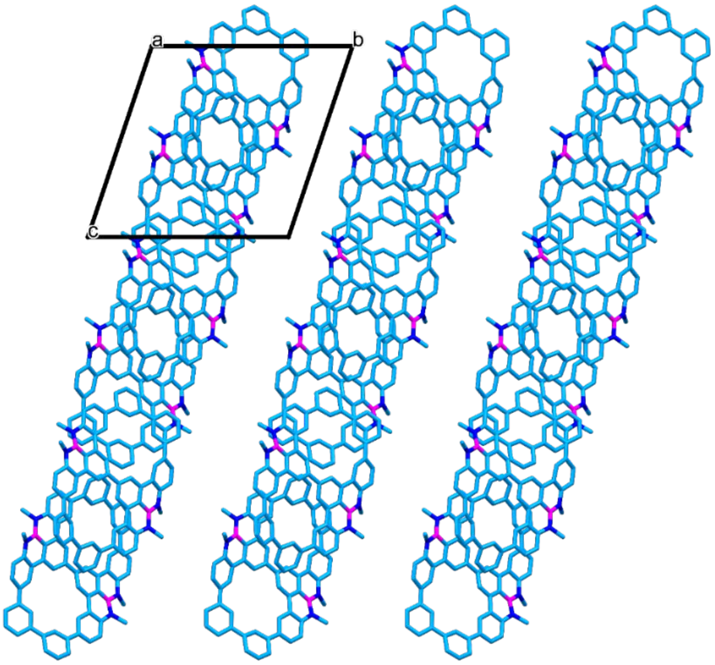

Figure S2. The packing pattern of **2PNG** in a 3×3×3 crystal cell stacked along the *a*-axis of the crystal lattice. Substituent groups and hydrogen atoms were omitted for clarity.

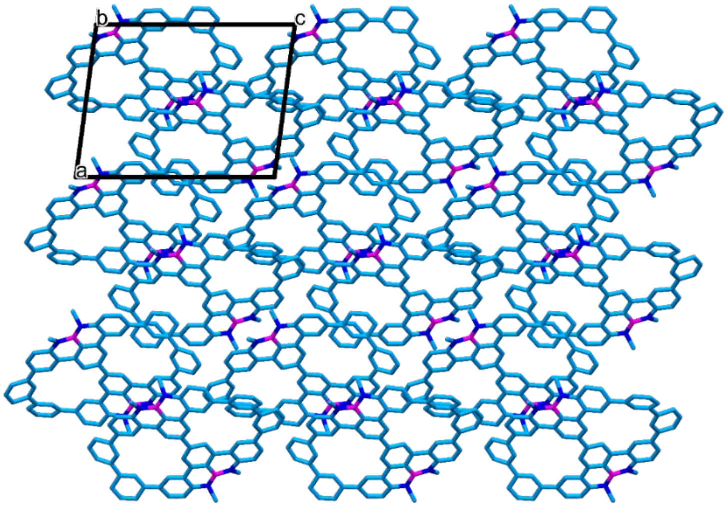

Figure S3. The packing pattern of **2PNG** in a 3×3×3 crystal cell stacked along the *b*-axis of the crystal lattice. Substituent groups and hydrogen atoms were omitted for clarity.

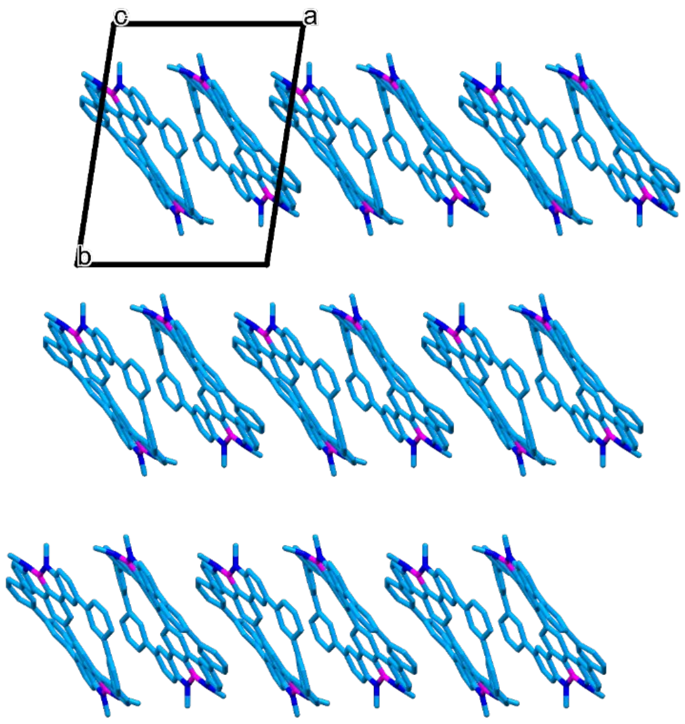

Figure S4. The packing pattern of 2PNG in a 3×3×3 crystal cell stacked along the *c*-axis of the crystal lattice. Substituent groups and hydrogen atoms were omitted for clarity.

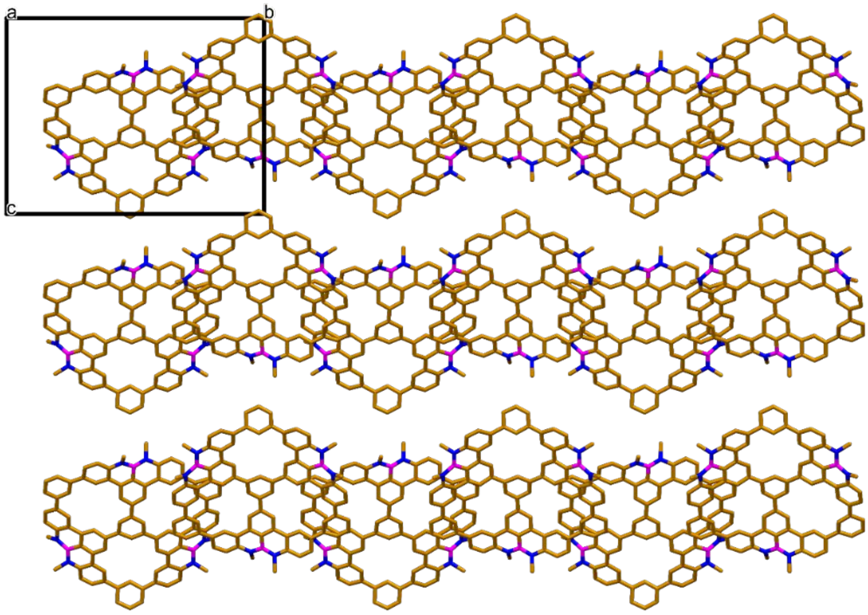

Figure S5. The packing pattern of 3PNG in a 3×3×3 crystal cell stacked along the *a*-axis of the crystal lattice. Substituent groups and hydrogen atoms were omitted for clarity.

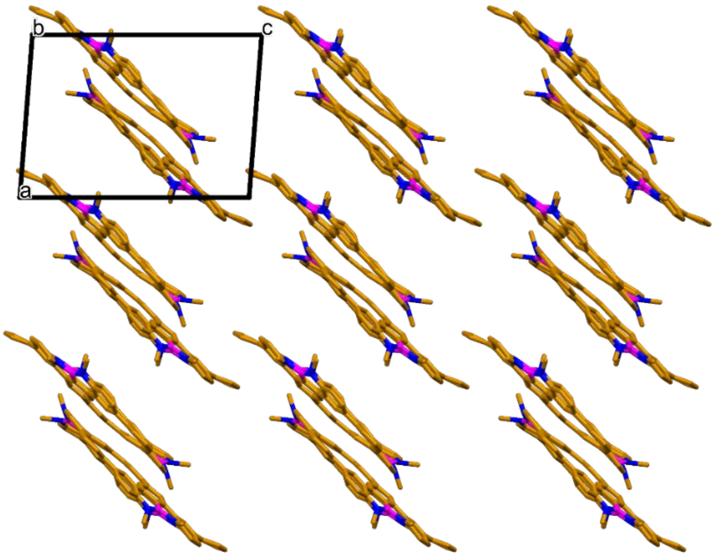

Figure S6. The packing pattern of 3PNG in a 3×3×3 crystal cell stacked along the *b*-axis of the crystal lattice. Substituent groups and hydrogen atoms were omitted for clarity.

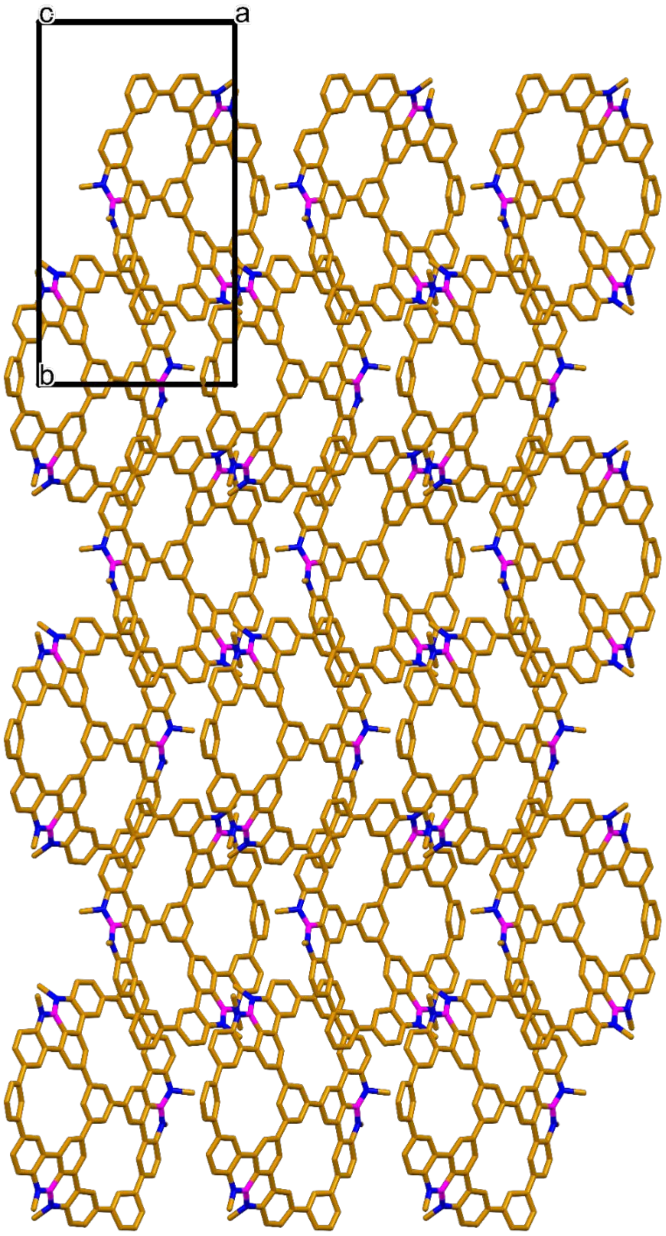

Figure S7. The packing pattern of 3PNG in a 3×3×3 crystal cell stacked along the *c*-axis of the crystal lattice. Substituent groups and hydrogen atoms were omitted for clarity.

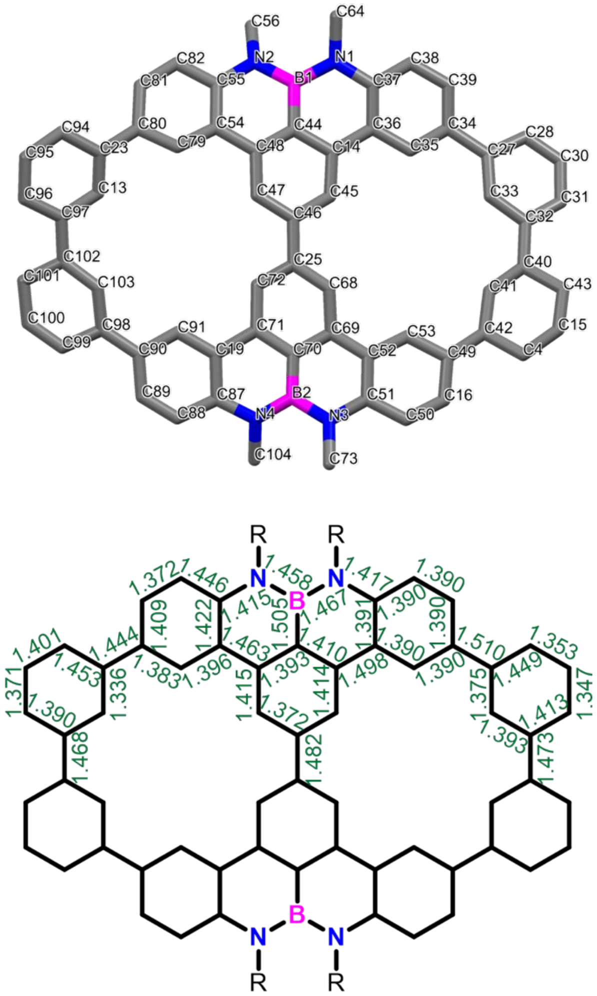

Figure S8. The atom label (up) and bond length (down) of **2PNG** in Å determined by X-ray crystallography. Bond lengths are highlighted in green. As the compounds were symmetric, the measurement was shown the up segments for clarity. Substituent groups were replaced by R.

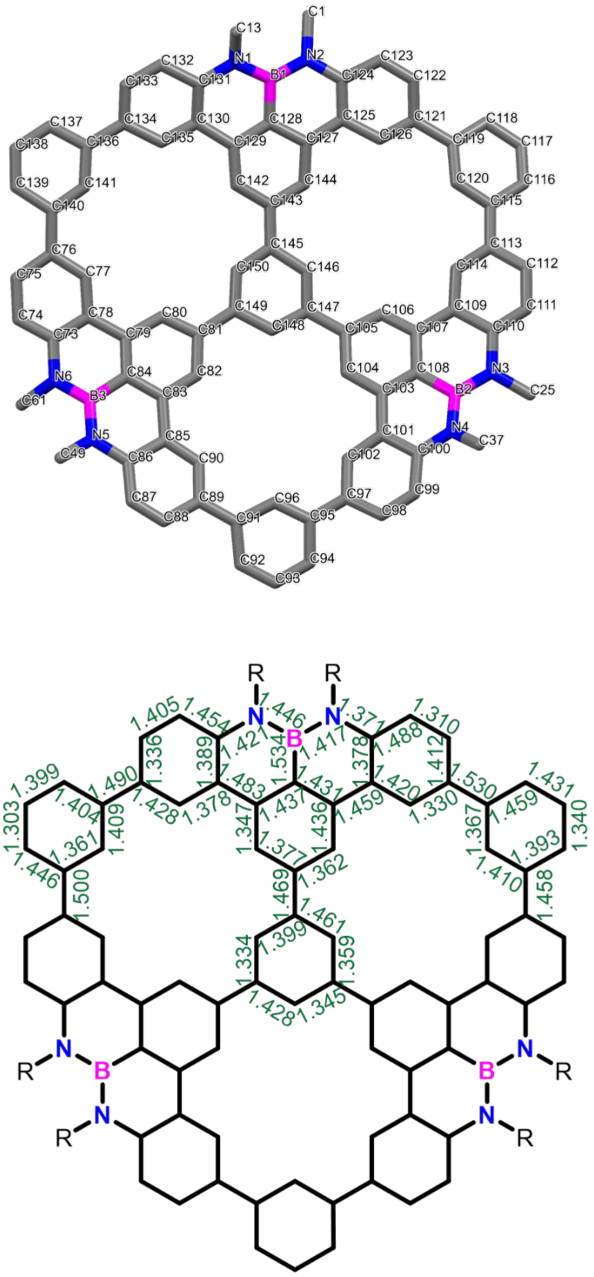

Figure S9. The atom label (up) and bond length (down) of **3PNG** in Å determined by X-ray crystallography. Bond lengths are highlighted in green. As the compounds were symmetric, the measurement was shown the up segments for clarity. Substituent groups were replaced by R.

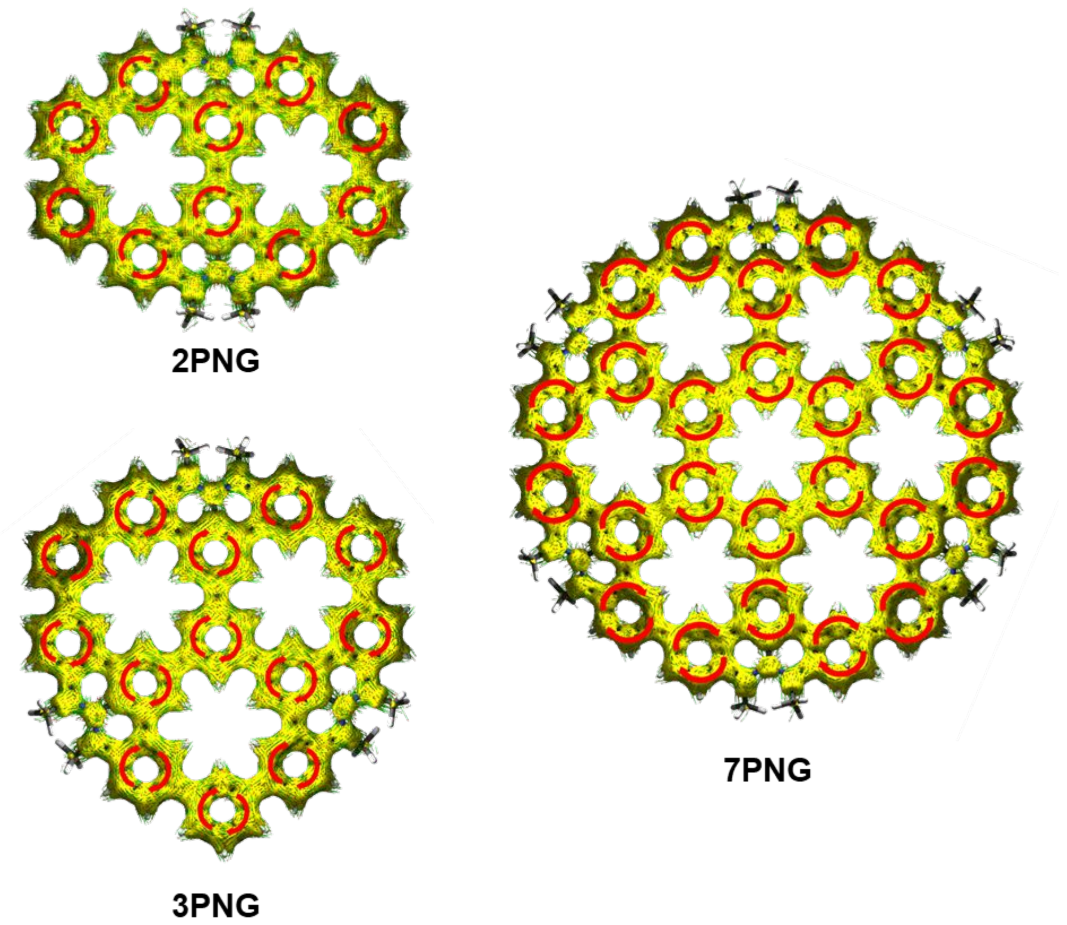

Figure S10. Calculated ACID plots for **2PNG**, **3PNG**, and **7PNG** calculated at the CSGT-B3LYP/6-311g(2d,p) level of theory. The diamagnetic (clockwise) and paramagnetic (counterclockwise) ring currents under the magnetic field parallel to the *z*-axis are highlighted by red and blue arrows, respectively.

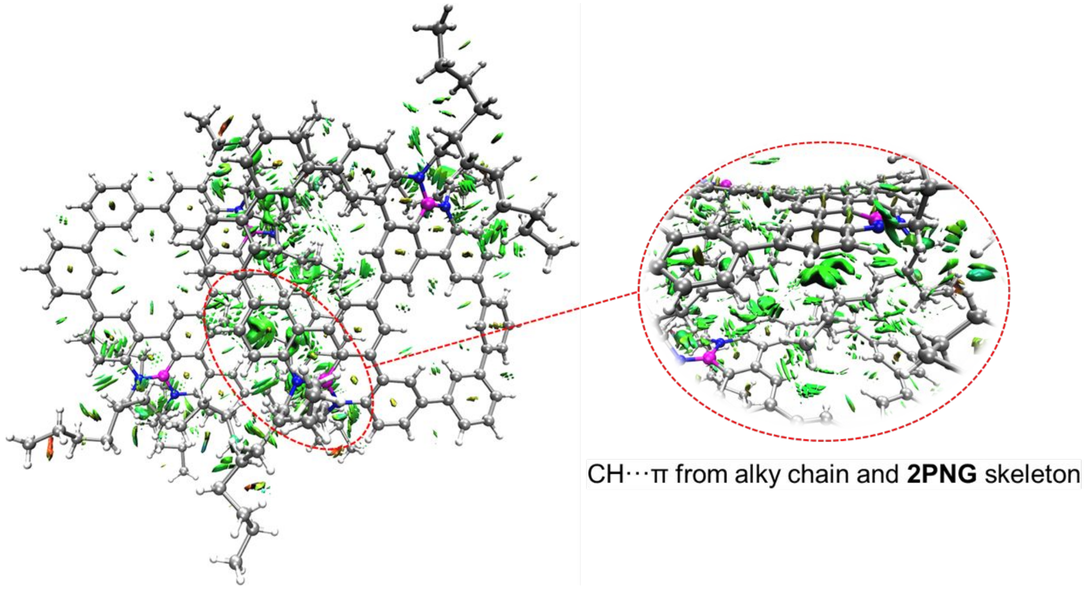

Figure S11. NCI plots of **2PNG**. Green isosurfaces showed weak van der Waals interactions. The blue and red isosurfaces showed regions with attractive and repulsive interactions, respectively. Attractive NCIs were depicted by the green isosurface, and a color code based on sign (λ_2) ρ was used: -0.05 au (blue) <0.0 au (green) <0.05 au (red).

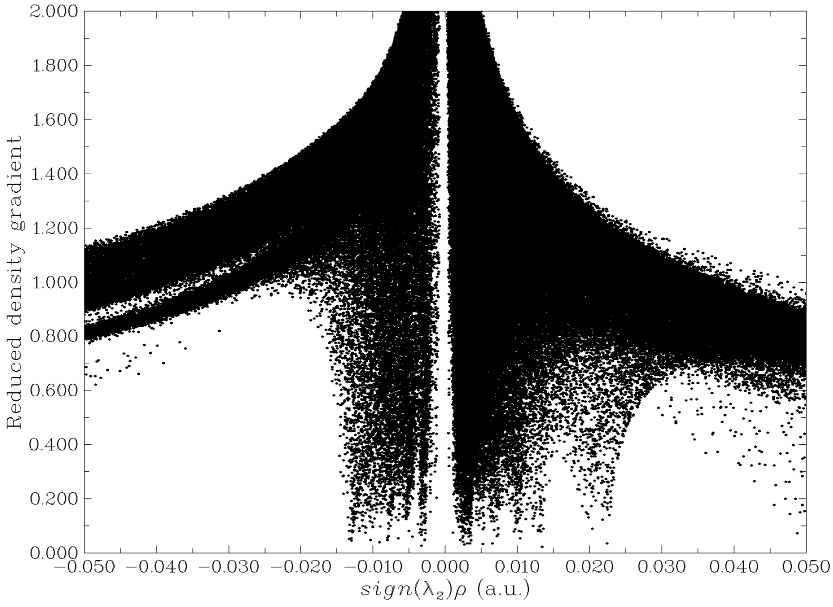

Figure S12. Plots of the reduced density gradient *versus* the electron density multiplied by the sign of the second Hessian eigenvalue for **2PNG**. The data was obtained by evaluating B3LYP/6-31G* density or promolecular density and gradient values on cuboid grids.

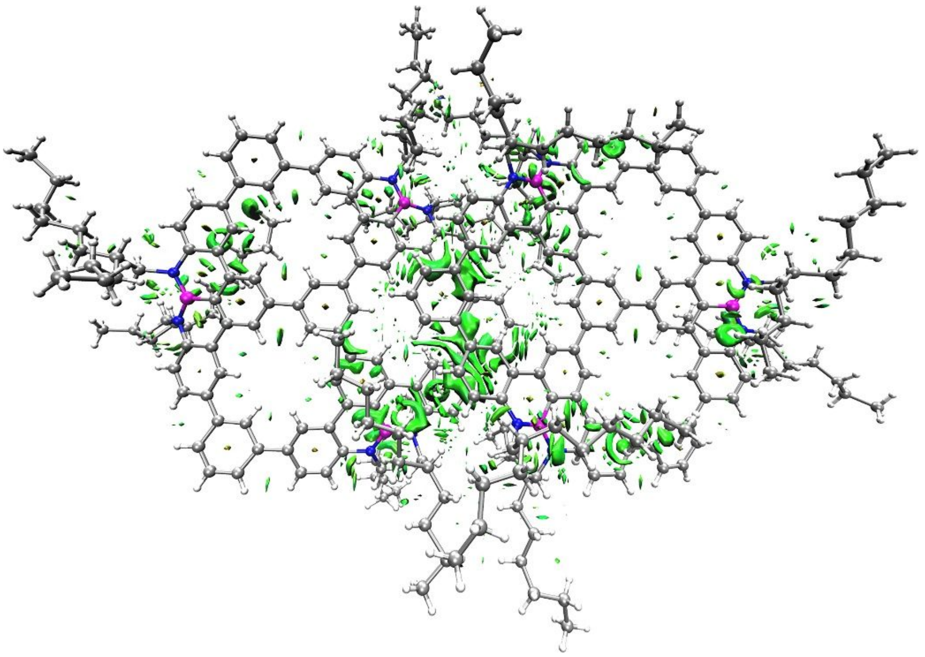

Figure S13. NCI plots of **3PNG**. Green isosurfaces showed weak van der Waals interactions. The blue and red isosurfaces showed regions with attractive and repulsive interactions, respectively. Attractive NCIs were depicted by the green isosurface, and a color code based on sign (λ_2) ρ was used: -0.05 au (blue) <0.0 au (green) <0.05 au (red).

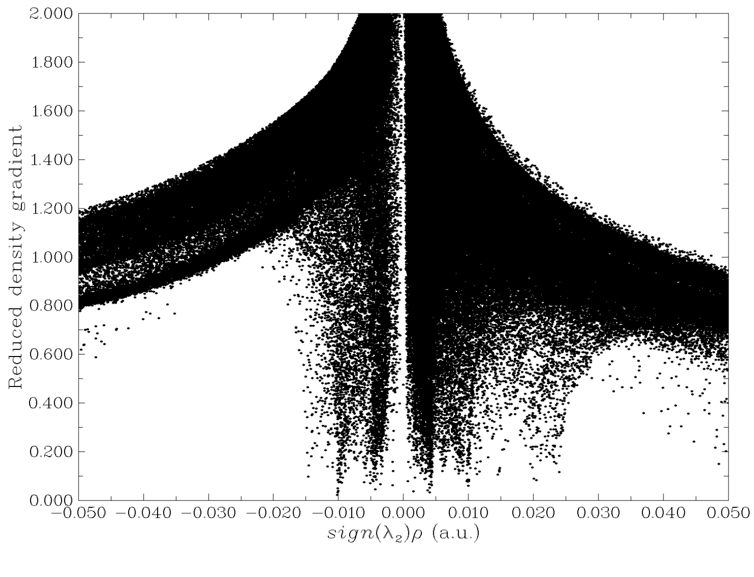

Figure S14. Plots of the reduced density gradient *versus* the electron density multiplied by the sign of the second Hessian eigenvalue for **3PNG**. The data was obtained by evaluating B3LYP/6-31G* density or promolecular density and gradient values on cuboid grids.

4. Self-assembly of PNGs at the solution-solid interface

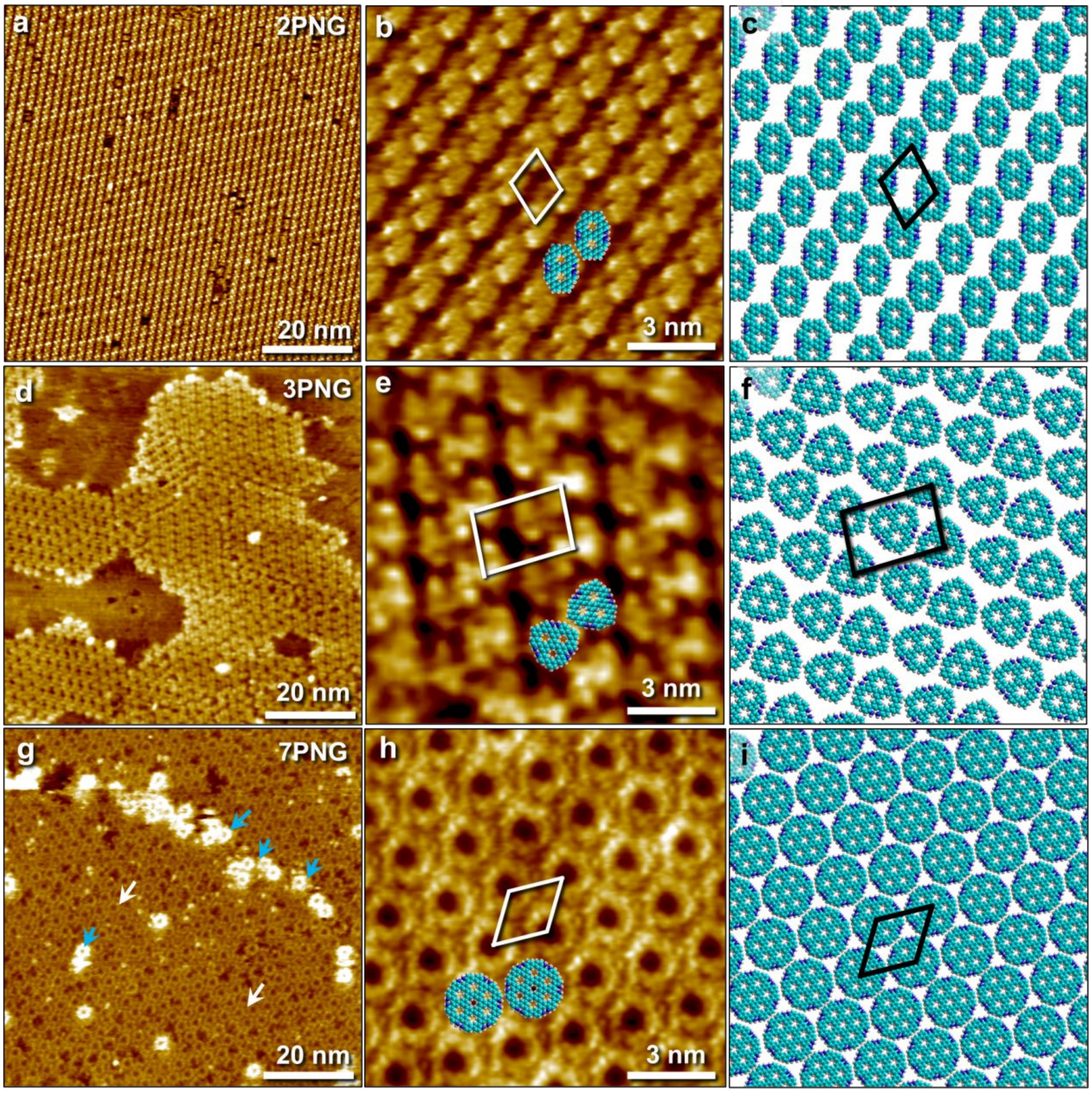

Figure S15. Self-assembly of PNGs at the solution-solid interface characterized using STM. a, **d**, **g**, Large-scale STM images of the different PNGs self-assembled at the 1-phenyloctane/graphite interface. In panel (**g**), white arrows indicate molecules adsorbed in the bottom layer whereas blue arrows highlight molecules in the second layer. **b**, **e**, **h**, Smaller-scale STM images of the self-assembled molecular networks of the PNGs. [**2PNG**] = 132 µM, [**3PNG**] = 261 µM, [**7PNG**] = 160 µM. For imaging parameters, see the experimental section. **c**, **f**, **i**, Tentative molecular models depicting the observed symmetry and arrangement of molecules within the self-assembled networks. These molecular models are based on experimentally obtained lattice parameters which in turn were obtained from calibrated STM data. The peripheral alkyl chains have been omitted for the sake of clarity and, in almost all cases, are only partially adsorbed on the graphite surface.

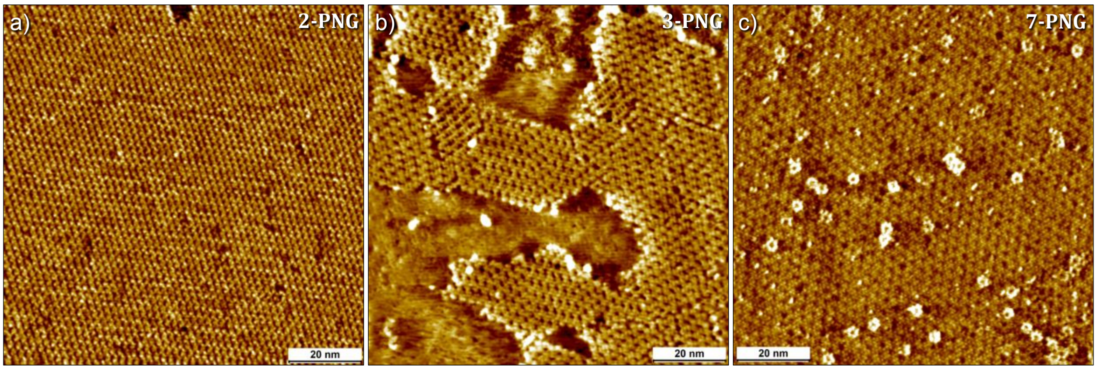

Figure S16. Additional large-scale STM images of the SAMNs formed by PNGs at the 1-phenyloctane/HOPG interface. [**2PNG**] = 132 μM, [**3PNG**] = 261 μM, [**7PNG**] = 160 μM. *Imaging conditions:* (a) V_{bias} = –1.10 V, I_{set} = 0.05 nA. (b) V_{bias} = –1.5 V, I_{set} = 0.10 nA, (c) V_{bias} = –1.40 V, I_{set} = 0.04 nA.

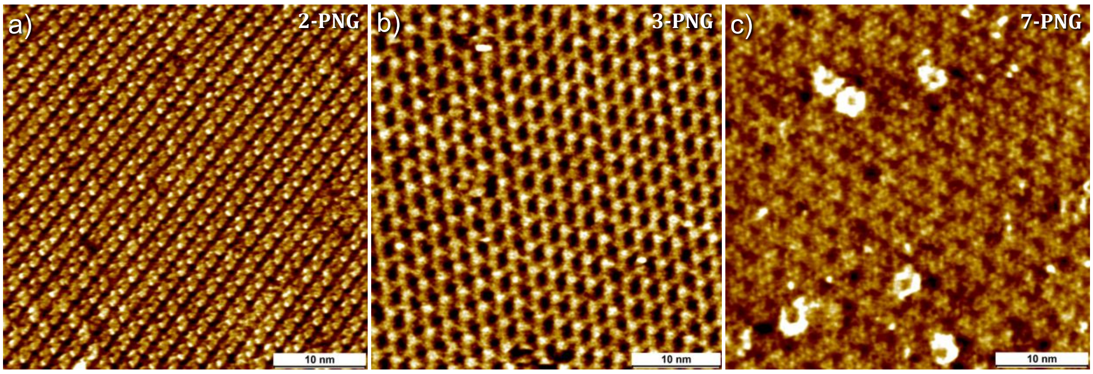

Figure S17. Additional small-scale STM images of the SAMNs formed by PNGs at the 1-phenyloctane/HOPG interface. [**2PNG**] = 132 μM, [**3PNG**] = 261 μM, [**7PNG**] = 160 μM. *Imaging conditions:* (a) V_{bias} = –1.12 V, I_{set} = 0.03 nA. (b) V_{bias} = –0.7 V, I_{set} = 0.06 nA. (c) V_{bias} = –1.40 V, I_{set} = 0.04 nA. These STM images are not calibrated using the graphite lattice.

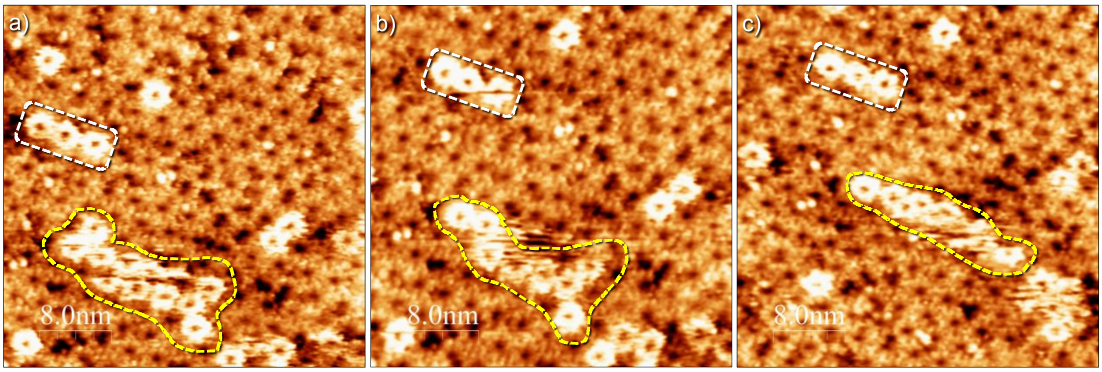

Figure S18. Sequential STM images showing STM tip-induced dynamics of **7PNG** molecules adsorbed in the second layer. The **7PNG** molecules adsorbed in the second layer undergo rearrangement highlighted by the areas marked by yellow dashed lines. The area marked by white dotted lines serves as a reference. *Imaging conditions:* V_{bias} = –1.40 V, I_{set} = 0.04 nA.

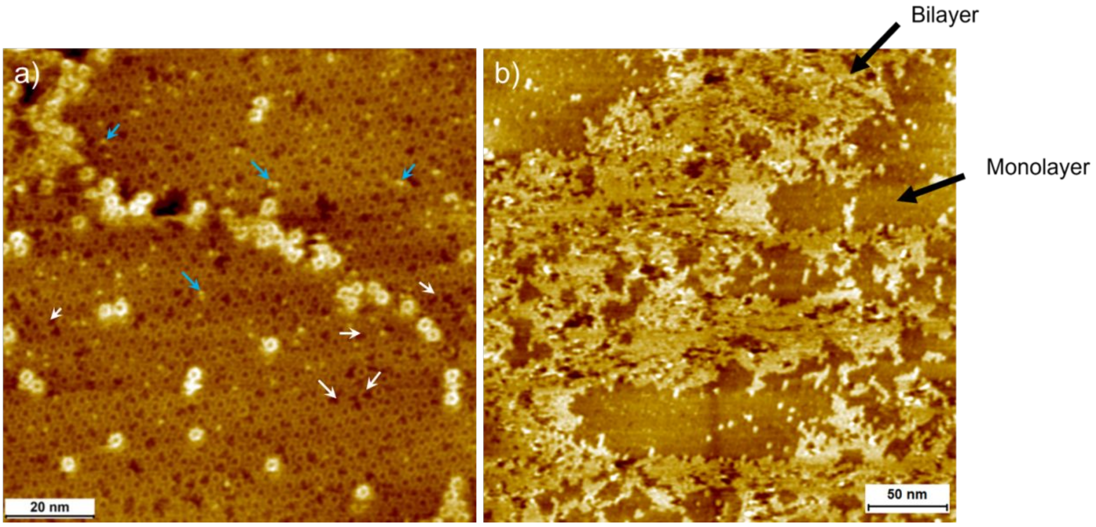

Figure S19. (a) STM image showing **7PNG** molecules which appear as 'open-ring' structures (white arrows). We hypothesize that these are disintegrated fragments of **7PNG** molecules that fall apart under the influence of voltage pulsing used for improving STM resolution. The 'missing' fragments could be the small bright features that were often observed in the STM images of **7PNG** (blue arrows). *Imaging conditions:* V_{bias} = –1.40 V, I_{set} = 0.04 nA. (b) Additional STM image showing **7PNG** molecules tendency to form a bilayer on HOPG surface. *Imaging conditions:* V_{bias} = –1.50 V, I_{set} = 0.10 nA.

Table S2. Unit cell parameters of the SAMNs formed by PNGs at the 1-phenyloctane graphite interface.

	Unit cell parameters			*N*	Plane group
	a (nm)	b (nm)	γ (°)		
**2PNG**	2.2 ± 0.1	1.8 ± 0.1	68 ± 2	1	*p2*
**3PNG**	3.8 ± 0.2	2.5 ± 0.1	84 ± 2	2	*p2*
**7PNG**	2.8 ± 0.2	2.8 ± 0.2	61 ± 2	1	*p6mm*

N = number of molecules per unit cell.

5. Electronic structure calculations

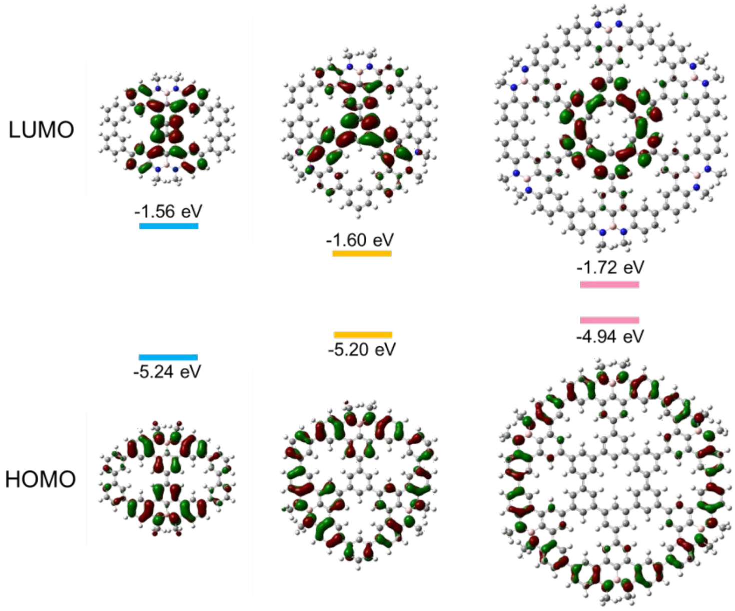

Figure S20. Optimized molecular geometries and frontier molecular orbitals (FMOs) of **2PNG**, **3PNG**, and **7PNG** were calculated at the B3LYP/6-311G(2d,p) level. The alkyl chains are omitted for clarity.

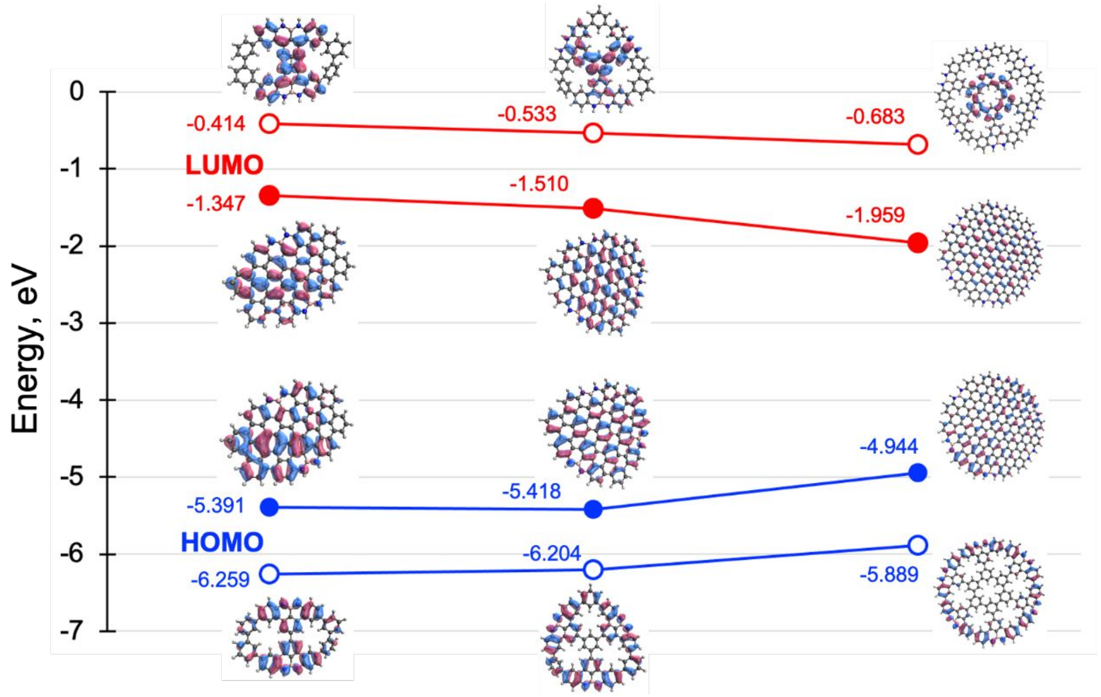

Figure S21. FMOs of PNGs. HOMO (blue) and LUMO (red) energy diagrams for the three studied PNGs (empty circles) and the nanographene analogues with no porous (full circles) computed at the M06-2X/6-31G(d) level.

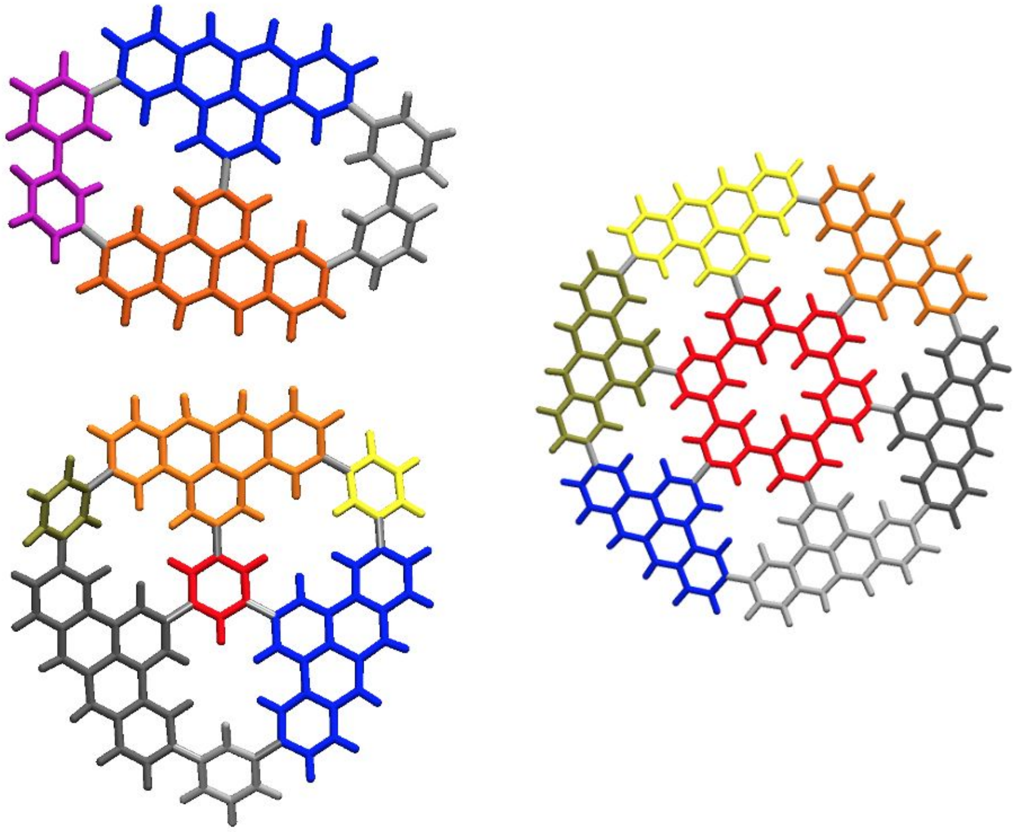

Figure S22. Definition of the molecular fragments employed for the analysis of the electronic character of singlet-singlet excitations. CT contributions correspond to excitations with hole and electron in different fragments.

Table S3. Transition energies (in eV) and charge transfer character (% CT) for the lowest-lying singlet excited states of **2PNG** and **3PNG** and **7PNG**. Computed at the M06-2X/6-31G(d) level.

	**2PNG**		**3PNG**		**7PNG**	
state	ΔE	% CT	ΔE	% CT	ΔE	% CT
S_1	4.10	25.2	4.06	26.9	3.87	35.4
S_2	4.13	13.7	4.10	25.5	3.91	34.1
S_3	4.18	23.4	4.14	23.5	3.95	29.6
S_4	4.27	13.6	4.16	17.2	3.96	28.3
S_5	4.51	34.5	4.16	19.3	3.98	31.8
S_6	4.64	31.2	4.25	15.3	3.98	28.2

Table S4. Triplet state excitation energies (in eV) and spin-orbit coupling constants (SOCC, in cm^{-1}) for the lowest-lying triplet states at the T_1 optimized geometry of **2PNG**. SOCCs computed for the two lowest excited singlet states (indicated in parenthesis). Calculations at the M06-2X/6-31G(d) level.

state	ΔE	SOCC(S_1)	SOCC(S_2)
T_1	3.275	0.004	0.227
T_2	3.304	0.056	1.076
T_3	3.599	0.307	0.001
T_4	3.621	0.416	0.014
T_5	3.705	0.120	0.003
T_6	3.741	0.009	0.046
T_7	3.778	0.792	0.281
T_8	3.835	0.084	0.173
T_9	3.885	0.001	0.020
T_{10}	4.047	0.298	0.004
T_{11}	4.078	0.218	0.082
T_{12}	4.079	0.285	0.059
T_{13}	4.136	0.218	0.003
T_{14}	4.161	0.013	0.156
T_{15}	4.235	0.000	0.030

Table S5. Triplet state excitation energies (in eV) and spin-orbit coupling constants (SOCC, in cm^{-1}) for the lowest-lying triplet states at the T_1 optimized geometry of **3PNG**. SOCCs computed for the three lowest excited singlet states (indicated in parenthesis). Calculations at the M06-2X/6-31G(d) level.

state	ΔE	SOCC(S_1)	SOCC(S_2)	SOCC(S_3)
T_1	3.111	1.063	0.118	0.106
T_2	3.163	0.256	0.087	0.168
T_3	3.165	0.239	0.826	0.139
T_4	3.452	0.345	0.193	0.082
T_5	3.465	0.124	0.346	0.153
T_6	3.476	0.066	0.093	0.450
T_7	3.547	0.020	0.116	0.180
T_8	3.589	0.037	0.313	0.469
T_9	3.596	0.059	0.170	0.321
T_{10}	3.620	0.090	0.201	0.187
T_{11}	3.706	0.061	0.144	0.114
T_{12}	3.787	0.126	0.171	0.263
T_{13}	3.801	0.128	0.019	0.212
T_{14}	3.821	0.150	0.164	0.150
T_{15}	3.853	0.220	0.152	0.181
T_{16}	4.008	0.101	0.156	0.098
T_{17}	4.010	0.105	0.052	0.224
T_{18}	4.026	0.045	0.112	0.053
T_{19}	4.035	0.052	0.110	0.168
T_{20}	4.093	0.064	0.092	0.079
T_{21}	4.102	0.080	0.177	0.078

Table S6. Triplet state excitation energies (in eV) and spin-orbit coupling constants (SOCC, in cm^{-1}) for the lowest-lying triplet states at the T_1 optimized geometry of **7PNG**. SOCCs computed for the three lowest excited singlet states (indicated in parenthesis). Calculations at the M06-2X/6-31G(d) level.

state	ΔE	SOCC(S_1)	SOCC(S_2)	SOCC(S_3)
T_1	3.221	0.000	0.067	0.778
T_2	3.236	0.062	0.000	0.001
T_3	3.237	0.086	0.000	0.001
T_4	3.262	0.000	0.113	0.226
T_5	3.285	0.001	0.915	0.811
T_6	3.297	1.006	0.001	0.003
T_7	3.435	0.000	0.090	0.596
T_8	3.495	0.067	0.000	0.000
T_9	3.506	0.000	0.138	0.160
T_{10}	3.534	0.046	0.000	0.002
T_{11}	3.538	0.027	0.000	0.000
T_{12}	3.540	0.000	0.105	0.625
T_{13}	3.546	0.041	0.000	0.002
T_{14}	3.554	0.000	0.170	0.806
T_{15}	3.562	0.165	0.000	0.003
T_{16}	3.576	0.082	0.000	0.006
T_{17}	3.584	0.000	0.126	0.530
T_{18}	3.589	0.000	0.152	0.660
T_{19}	3.614	0.220	0.000	0.004
T_{20}	3.667	0.224	0.001	0.008
T_{21}	3.671	0.001	0.097	0.310
T_{22}	3.679	0.000	0.208	0.080
T_{23}	3.759	0.000	0.011	0.477
T_{24}	3.817	0.057	0.000	0.003
T_{25}	3.851	0.000	0.116	0.677
T_{26}	3.852	0.039	0.000	0.005
T_{27}	3.889	0.152	0.000	-0.002
T_{28}	3.893	0.001	0.040	-0.215
T_{29}	3.900	0.095	0.001	-0.016
T_{30}	3.922	0.000	0.301	-0.403

6. Photophysical properties

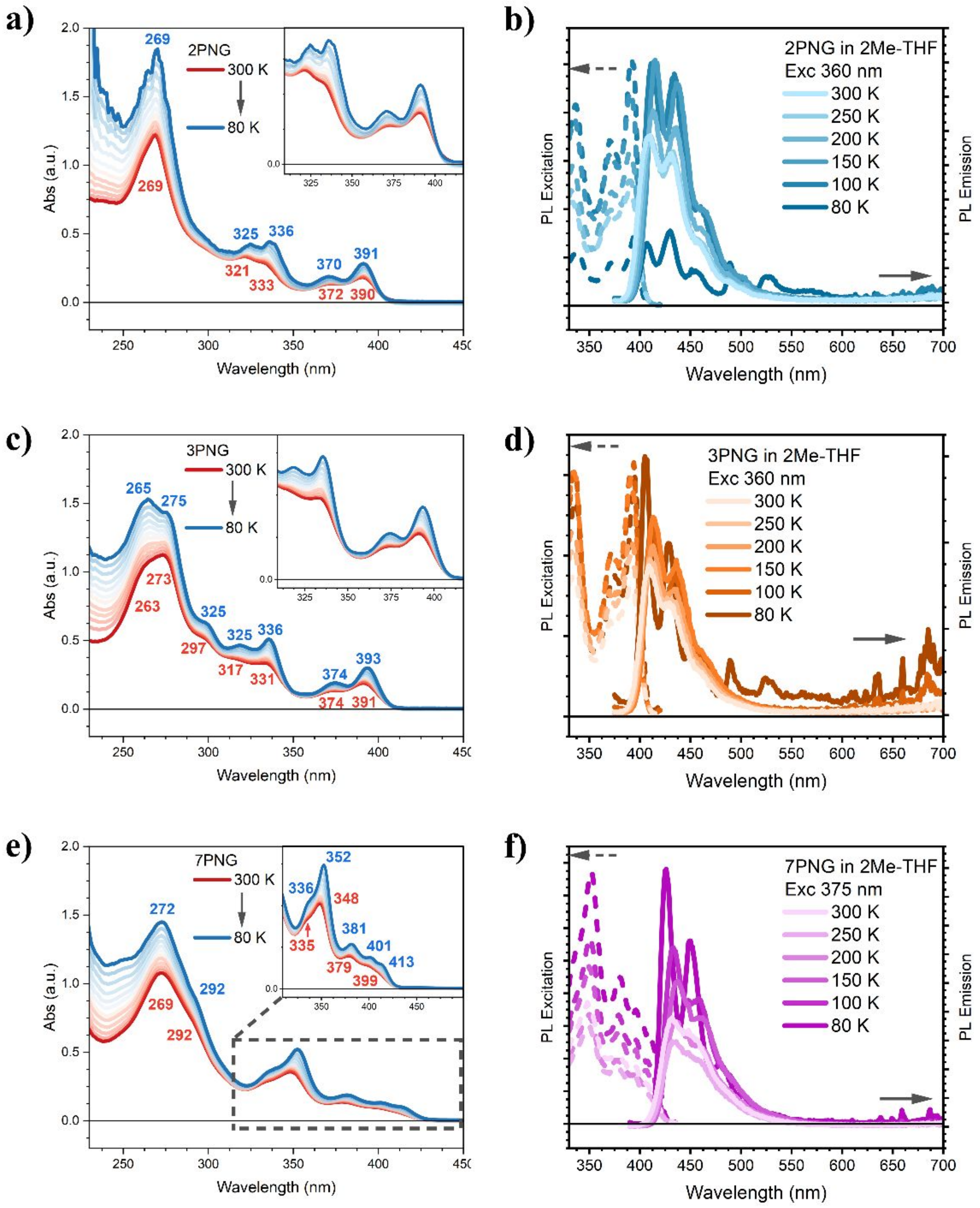

Figure S23. Absorption spectra obtained in a range of temperatures from 300 to 80 K for **a) 2PNG**, **c) 3PNG** and **e) 7PNG**. Excitation (dashed lines) and emission spectrum (solid lines) obtained in a range of temperatures from 300 to 80 K for **b) 2PNG**, **d) 3PNG** and **f) 7PNG.**

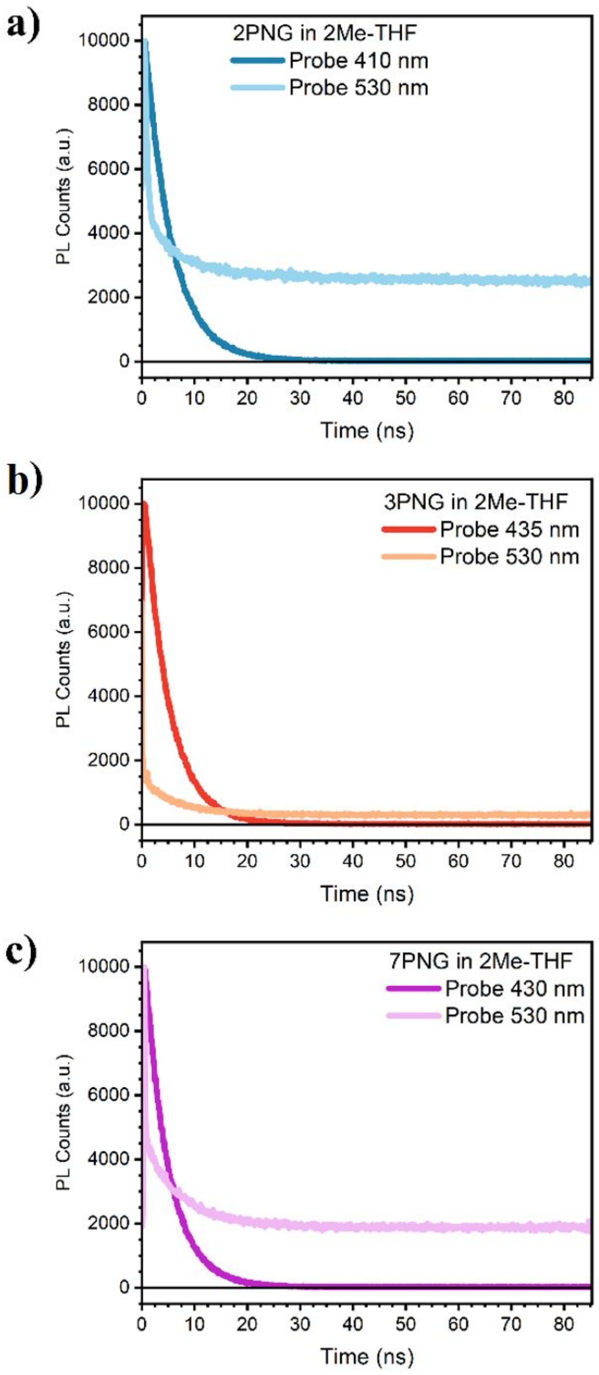

Figure S24. Emission decays obtained at 80 K at 410, 435 and 430 nm (darker lines) for **a) 2PNG, b) 3PNG** and **c) 7PNG** at 530 nm (lighter lines).

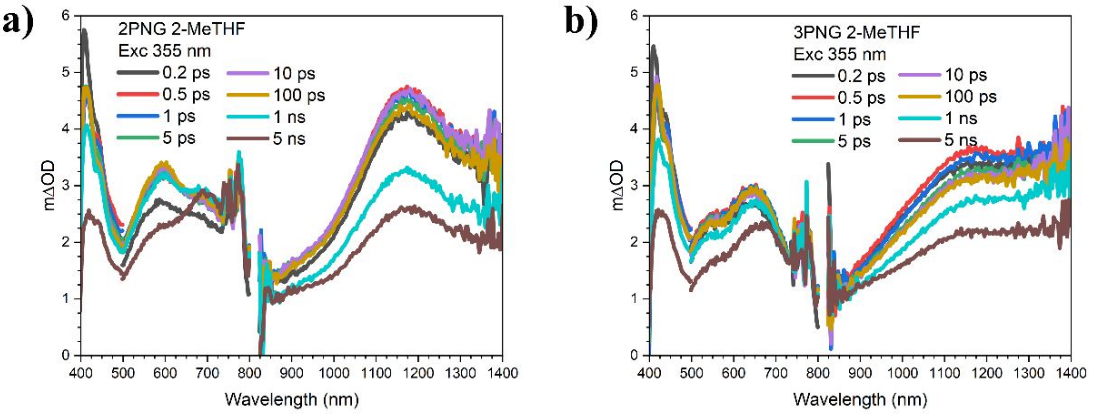

Figure S25. Fs-TA spectra at several delays, from 0.2 ps to 5 ns of **a) 2PNG** and **b) 3PNG**. The data was obtained upon excitation at 355 nm at 0.25 mW.

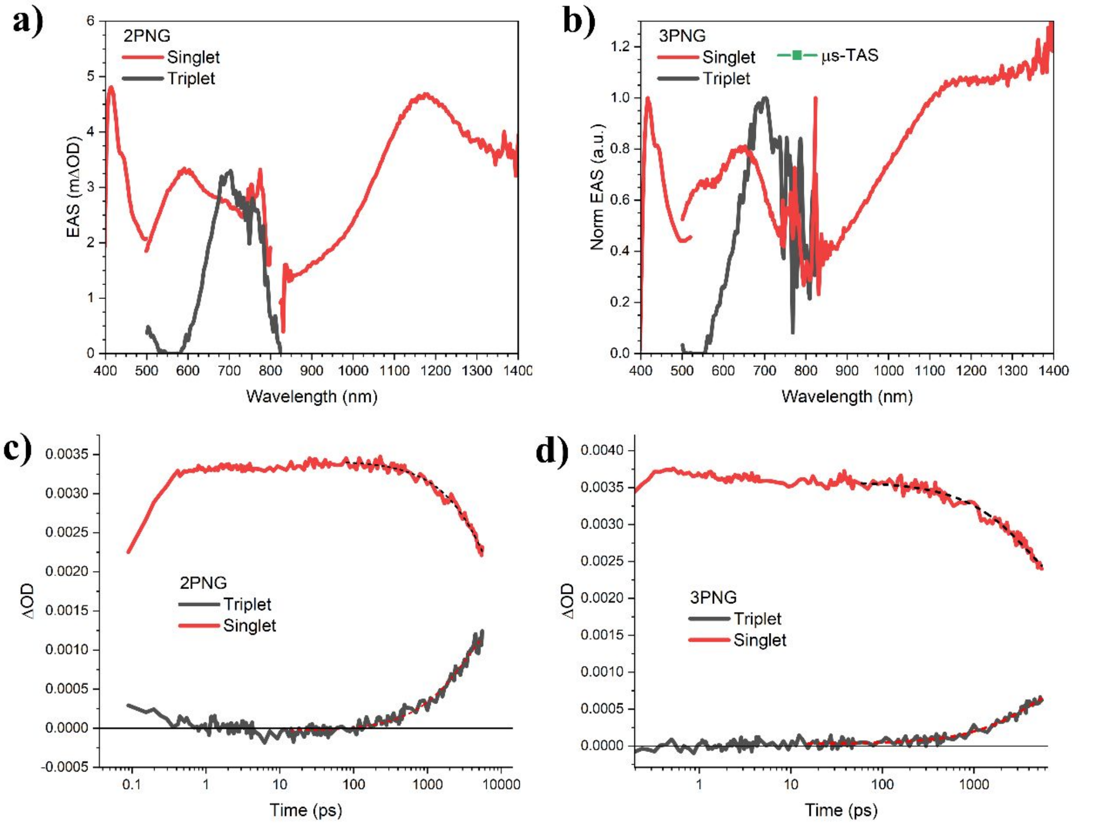

Figure S26. Results from the Global Analysis of the fs-TAS. The results are divided in the spectra for the species obtained for **a) 2PNG** and **b) 3PNG** and the decays associated with species of **c) 2PNG** and **d) 3PNG**.

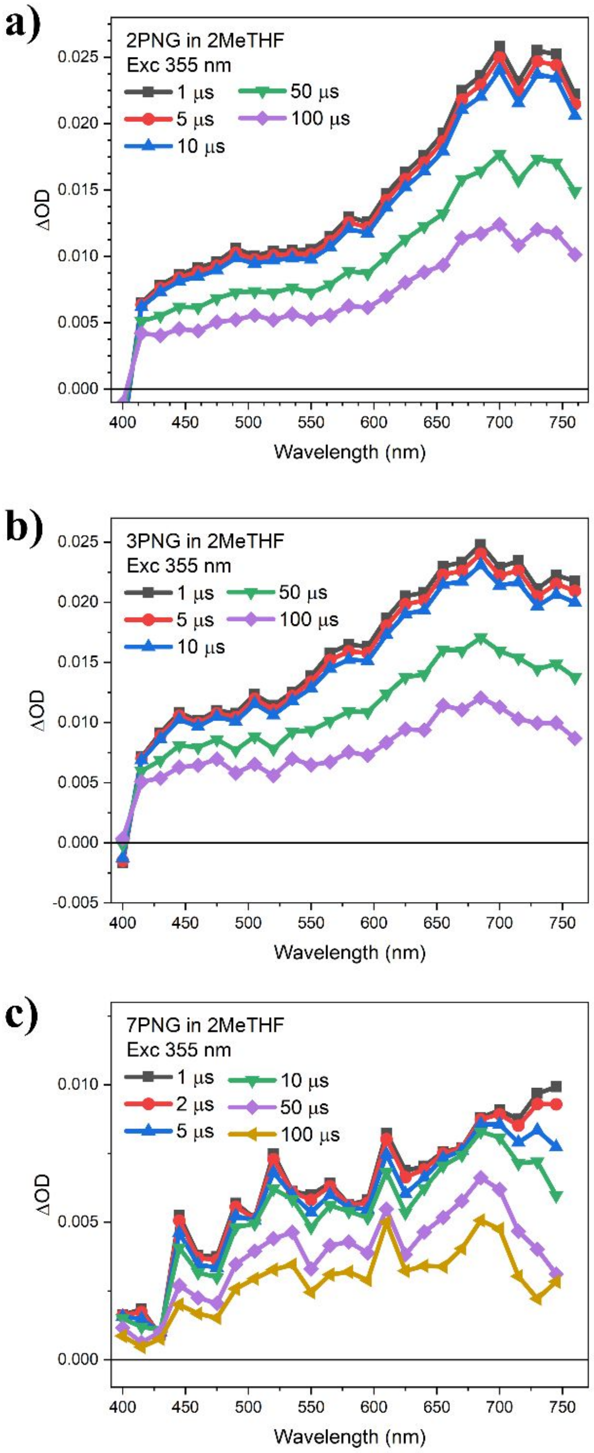

Figure S27. μs-TAS spectra obtained at several time delays of 2-MeTHF solutions of **a) 2PNG**, **b) 3PNG**, and **c) 7PNG** upon excitation at 355 nm with a 15 mJ.

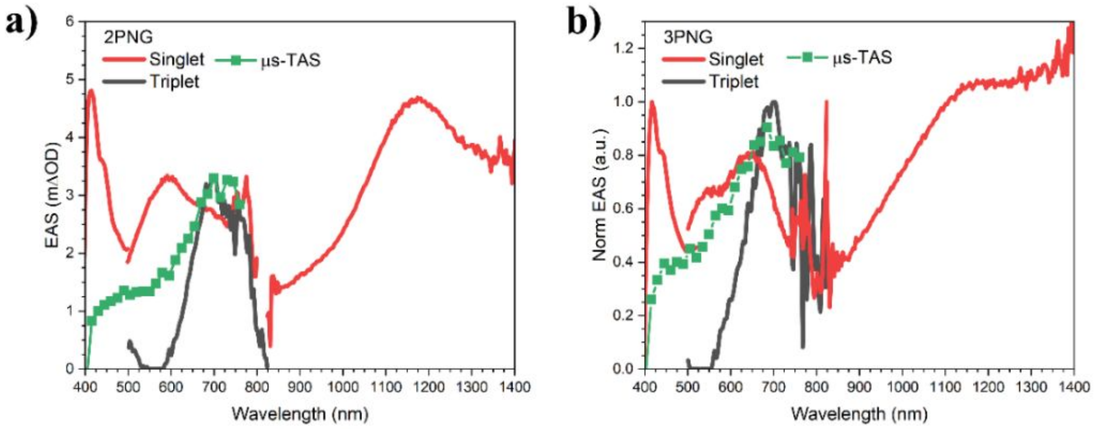

Figure S28. Comparison of the μs-TA spectrum and the species obtained in the Global analysis of the fs-TAS data for **a) 2PNG** and **b) 3PNG**.

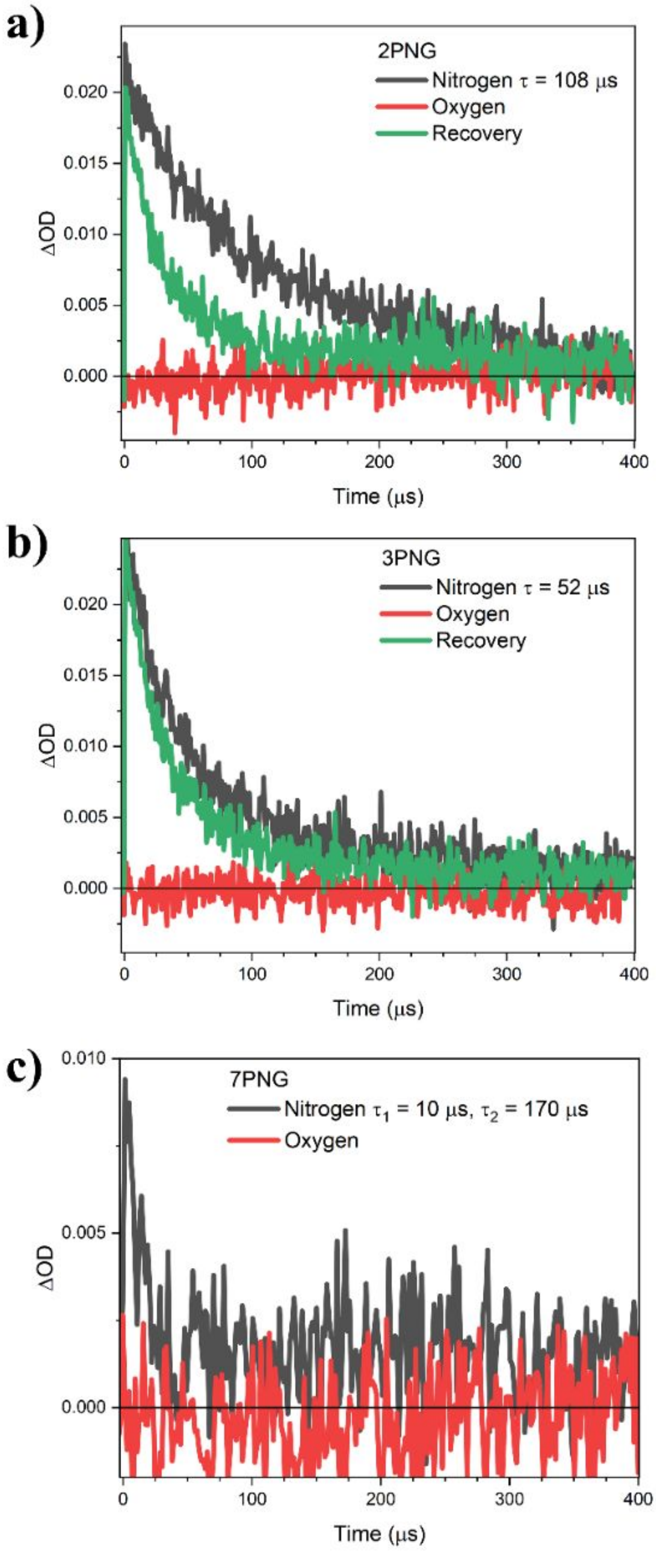

Figure S29. Oxygen dependence of the μs-TAS signal for **a) 2PNG, b) 3PNG,** and **c) 7PNG** probed at 650, 650, and at 700 nm, respectively upon 355 nm excitation.

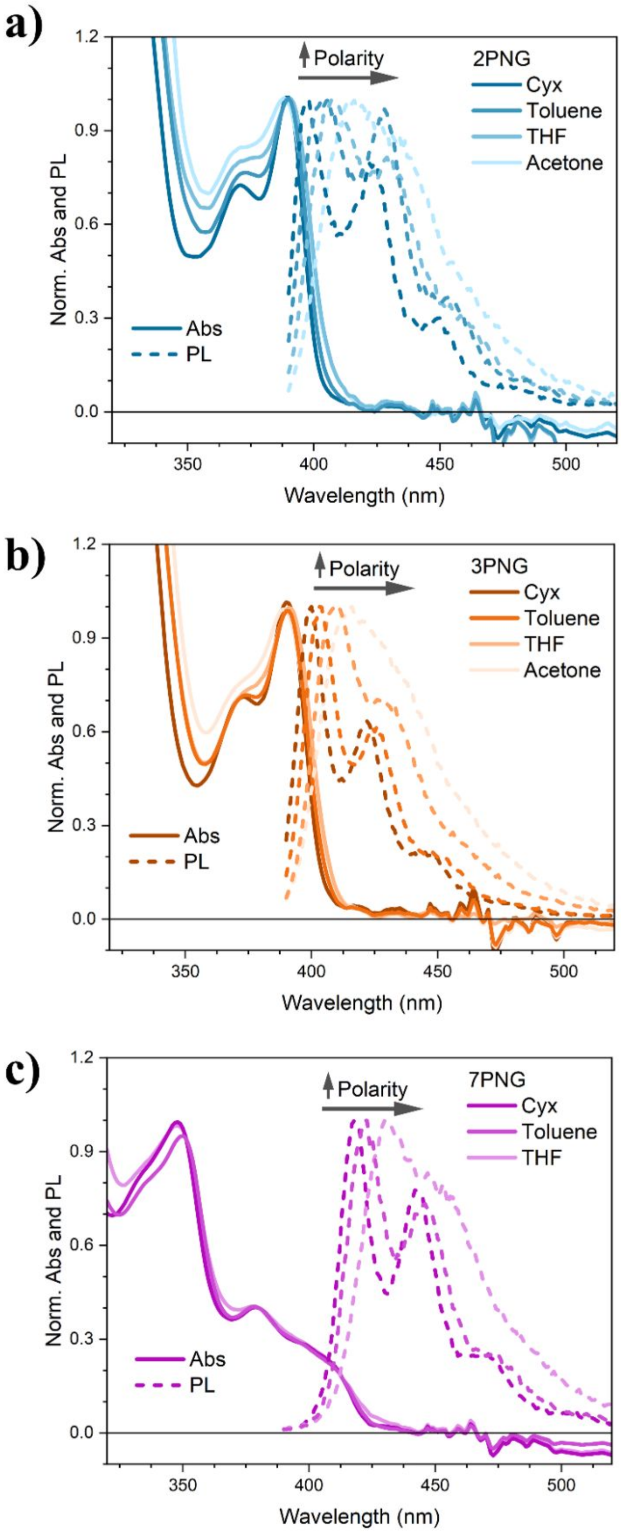

Figure S30. Absorption and emission spectra of **a) 2PNG, b) 3PNG,** and **c) 7PNG** in solutions using solvents with different polarity (Cyx=cyclohexane, toluene, THF, and acetone). An arrow has been added to help the reader to see the trend in solvent polarity.

7. NMR spectra

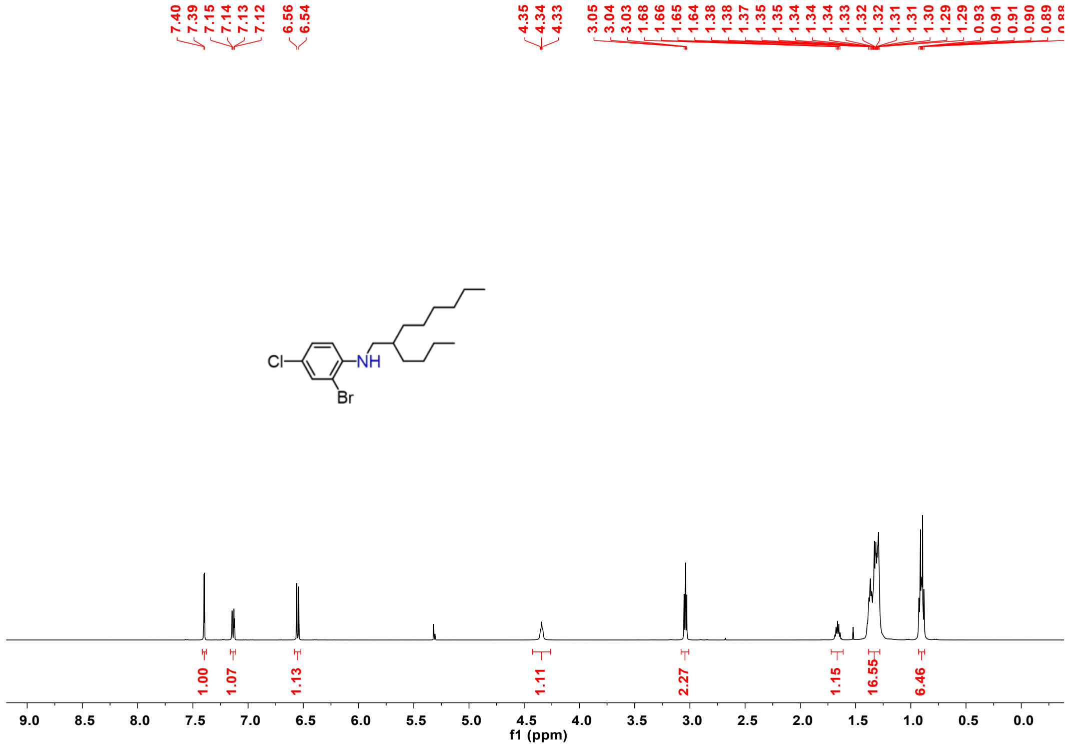

Figure S31. ^{1}H NMR spectrum of **2b** in CD_2Cl_2 (500 MHz, 298 K).

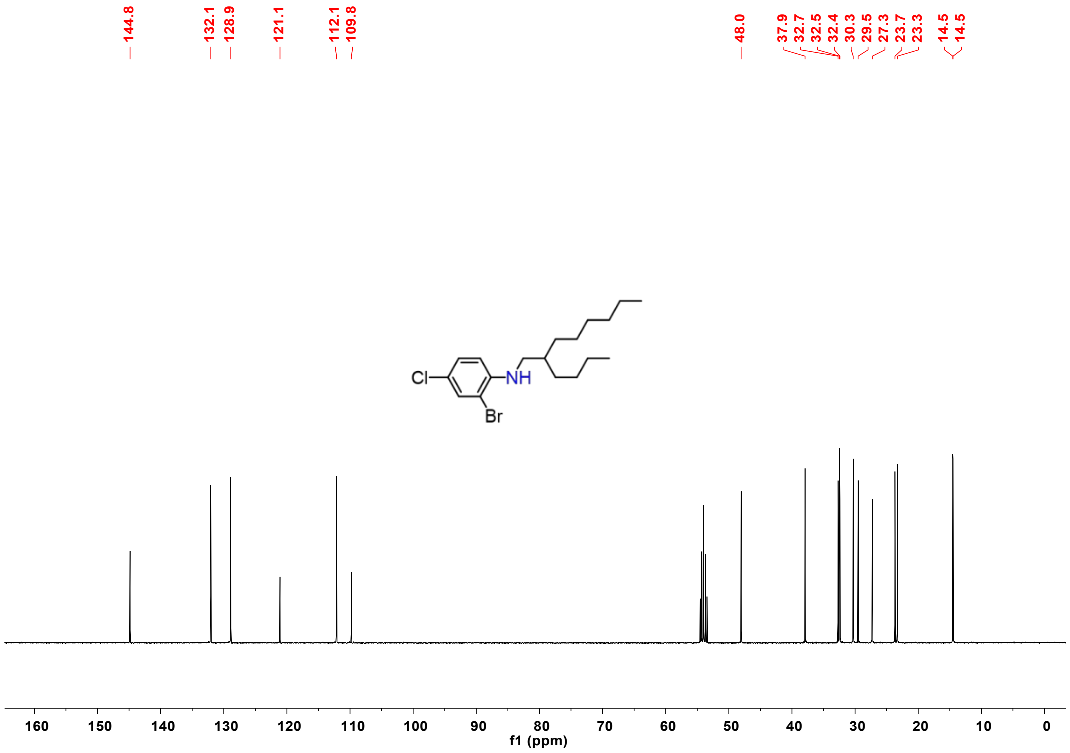

Figure S32. ^{13}C NMR spectrum of **2b** in CD_2Cl_2 (101 MHz, 298 K).

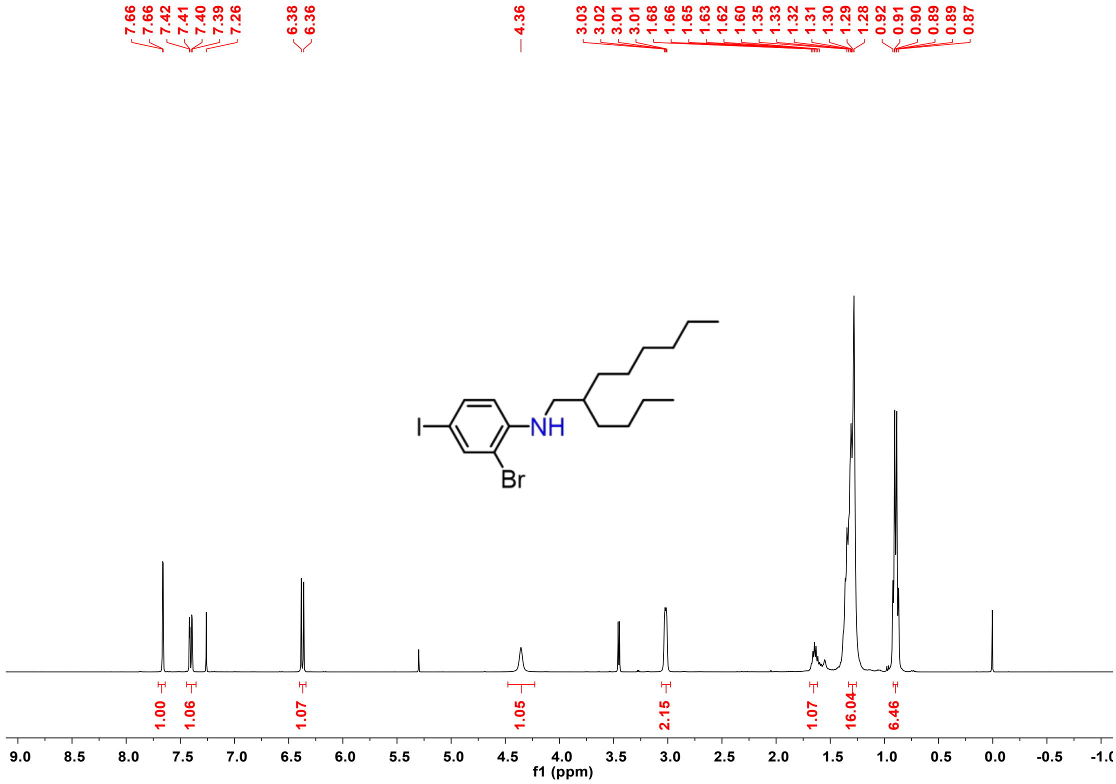

Figure S33. ^{1}H NMR spectrum of **2c** in $CDCl_3$ (400 MHz, 298 K).

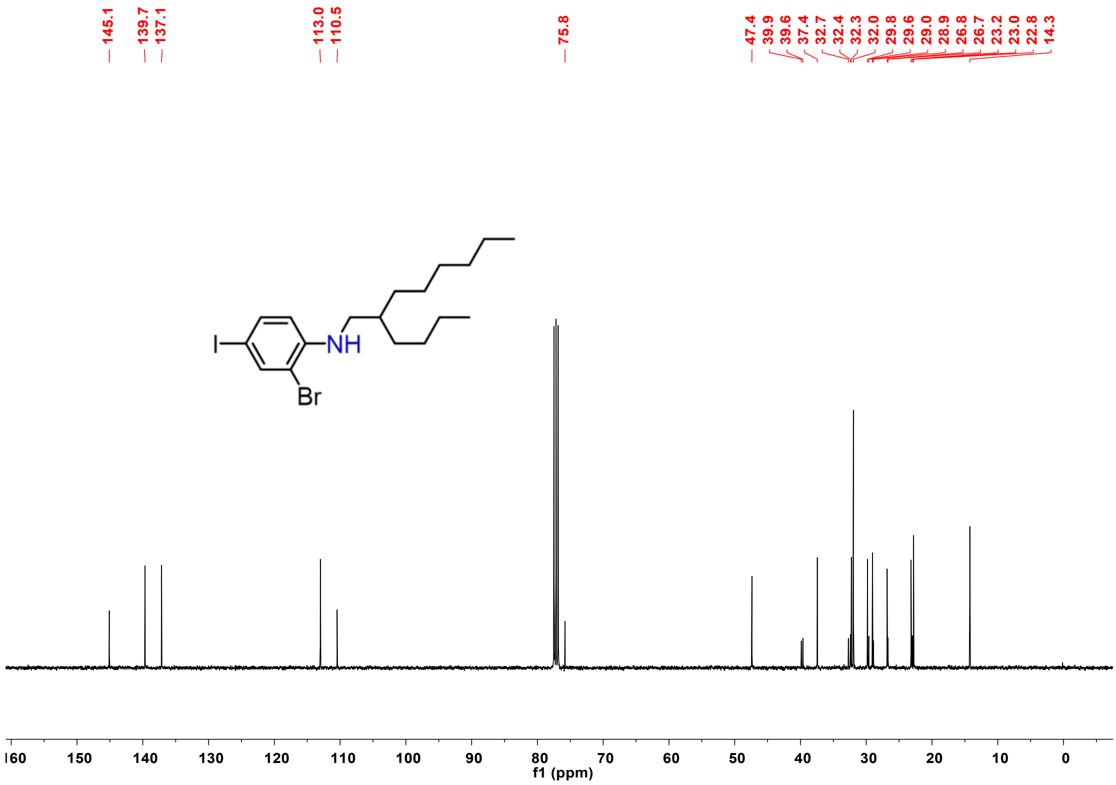

Figure S34. ^{13}C NMR spectrum of **2c** in $CDCl_3$ (101 MHz, 298 K).

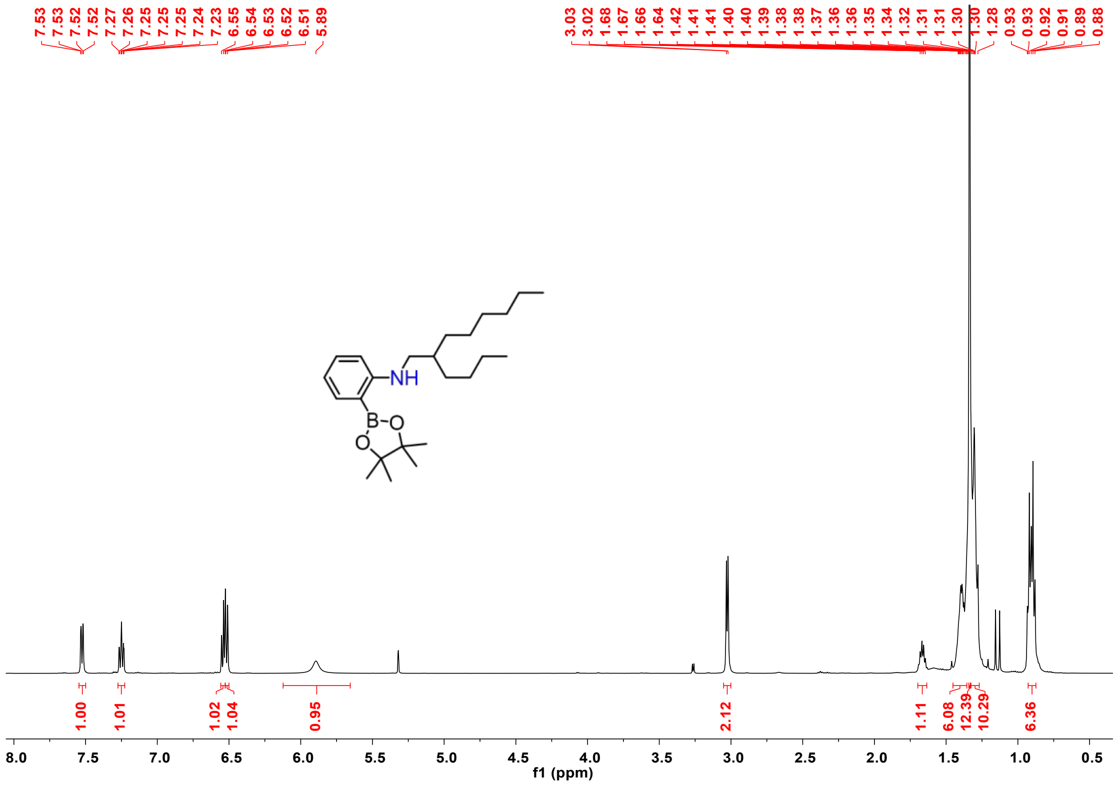

Figure S35. ^{1}H NMR spectrum of **3a** in CD_2Cl_2 (500 MHz, 298 K).

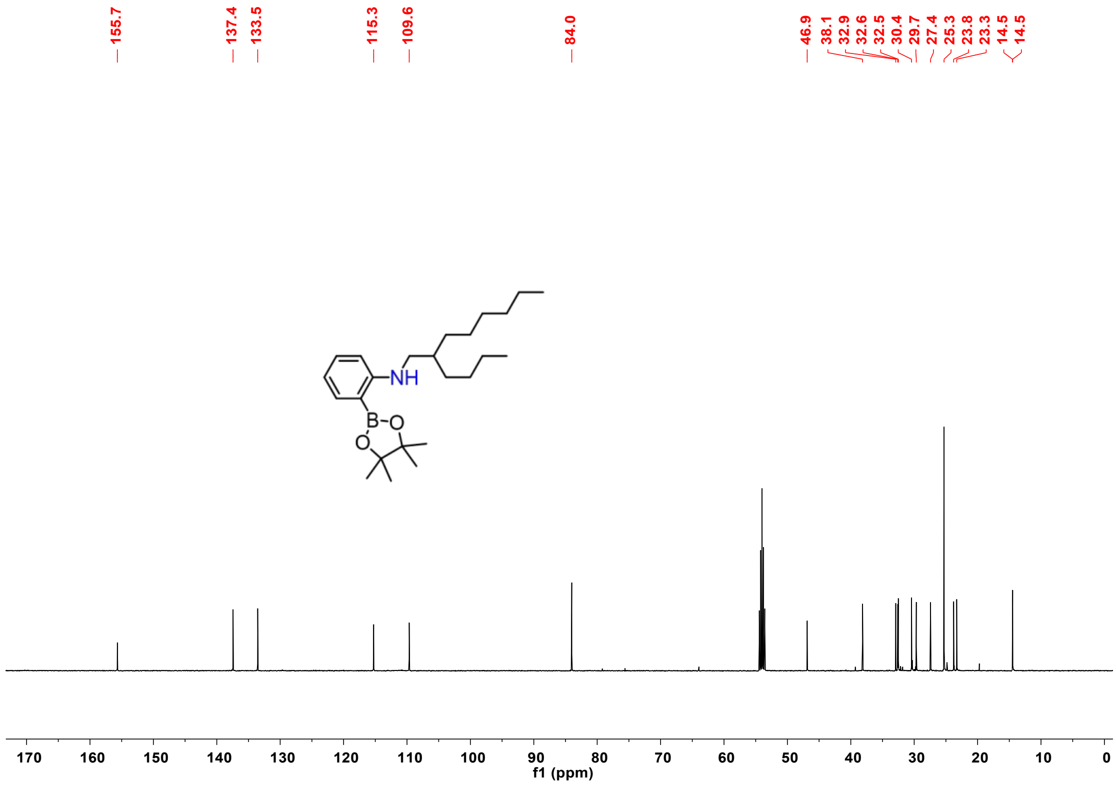

Figure S36. ^{13}C NMR spectrum of **3a** in CD_2Cl_2 (126 MHz, 298 K).

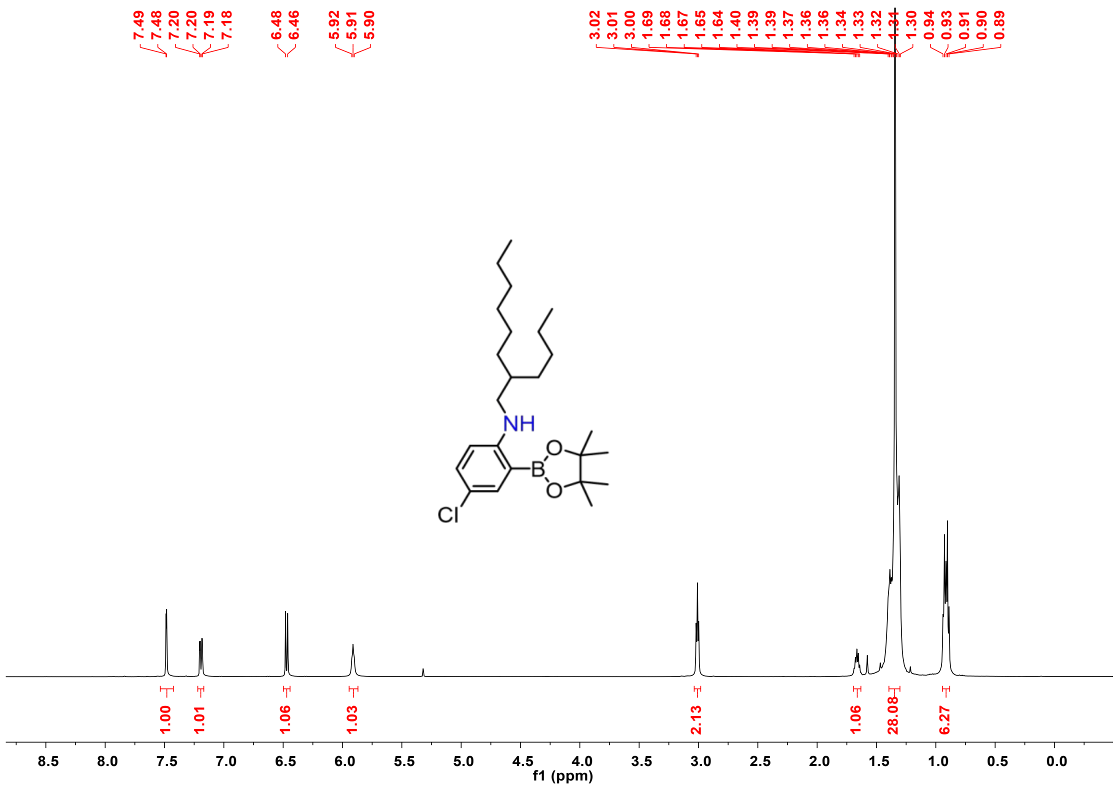

Figure S37. ^{1}H NMR spectrum of **3b** in CD_2Cl_2 (500 MHz, 298 K).

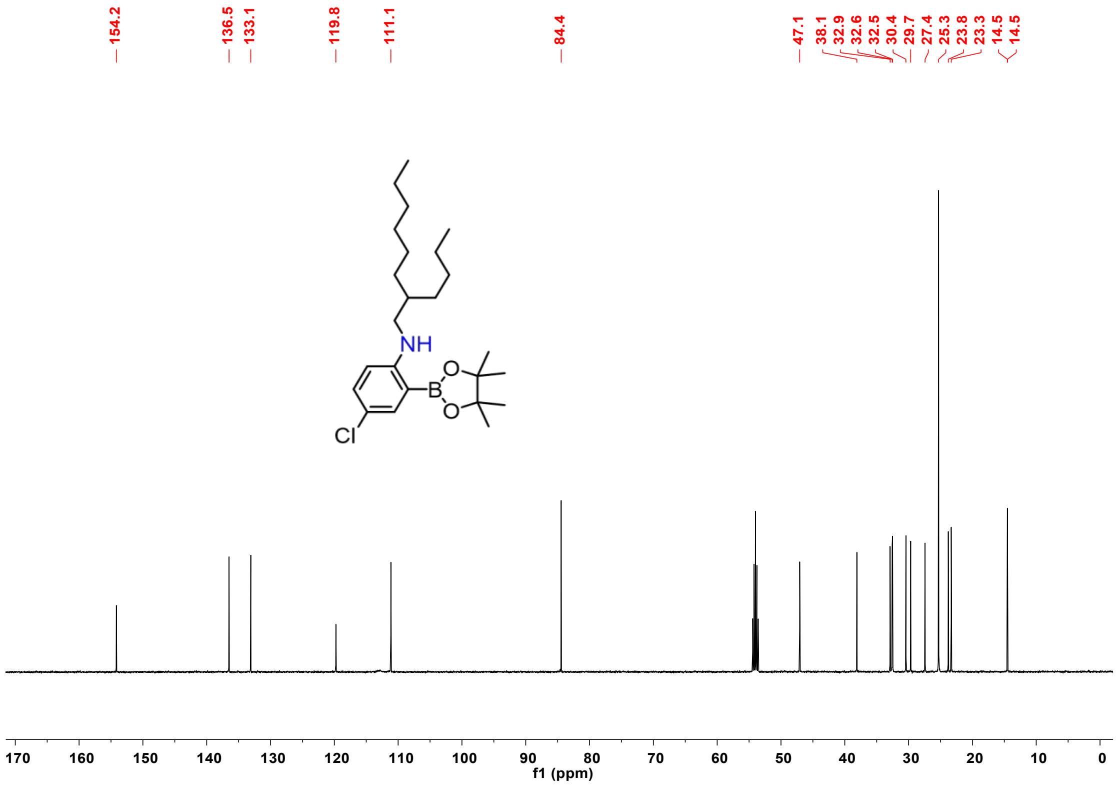

Figure S38. ^{13}C NMR spectrum of **3b** in CD_2Cl_2 (126 MHz, 298 K).

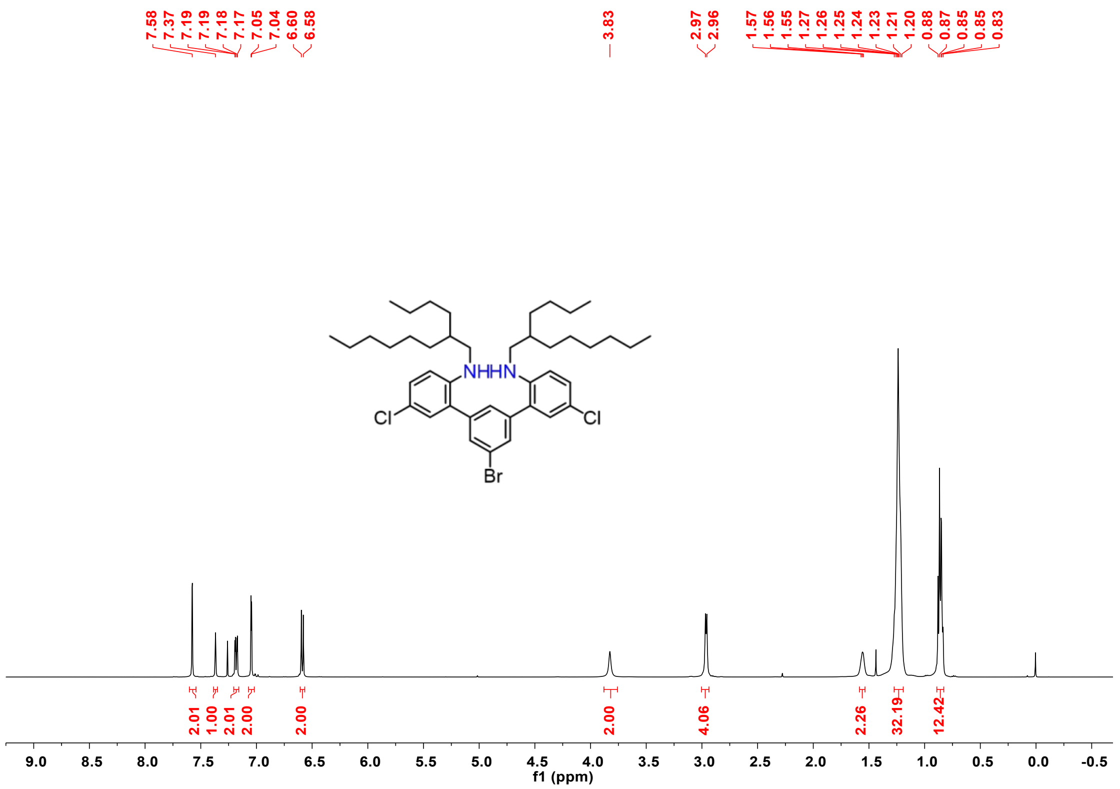

Figure S39. ^{1}H NMR spectrum of **4** in $CDCl_3$ (500 MHz, 298 K).

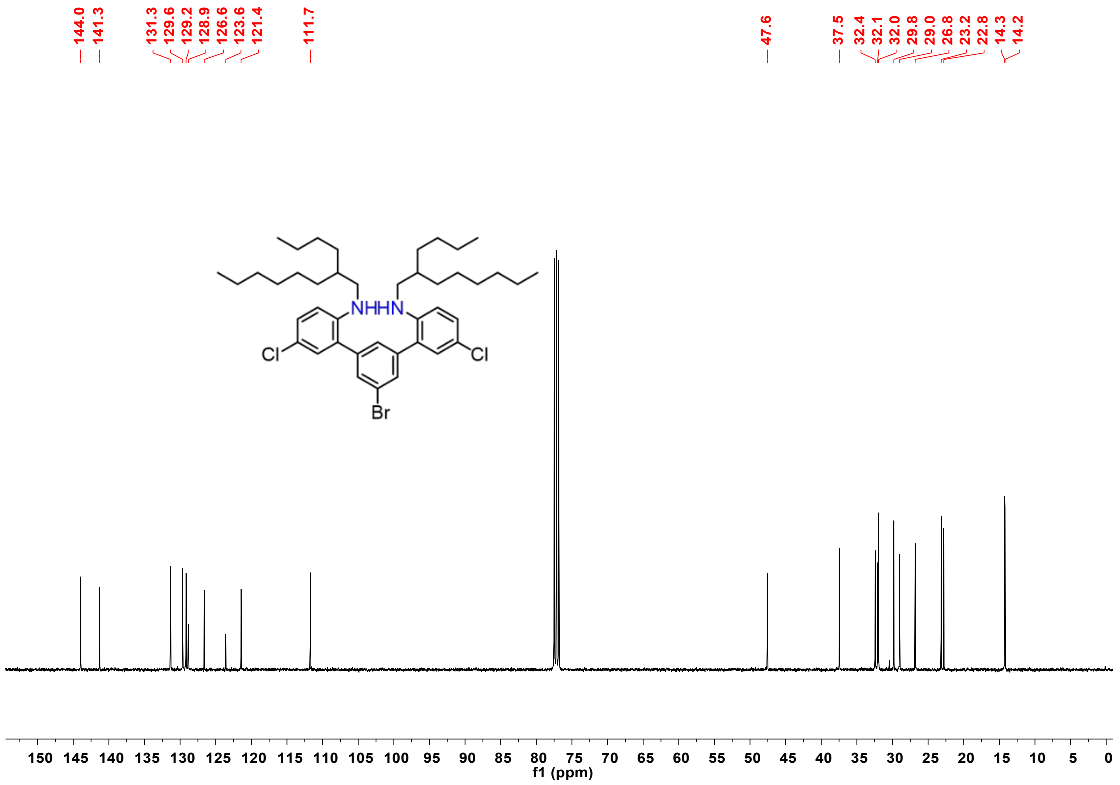

Figure S40. ^{13}C NMR spectrum of **4** in $CDCl_3$ (101 MHz, 298 K).

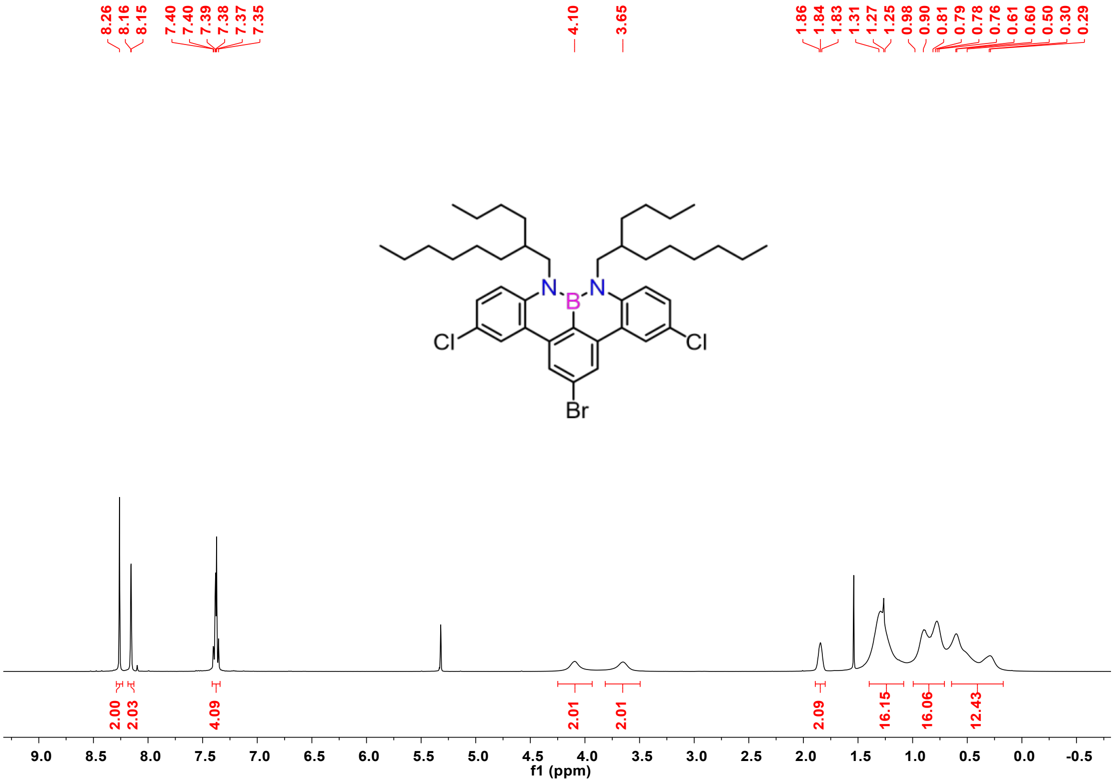

Figure S41. ^{1}H NMR spectrum of **5** in CD_2Cl_2 (500 MHz, 298 K).

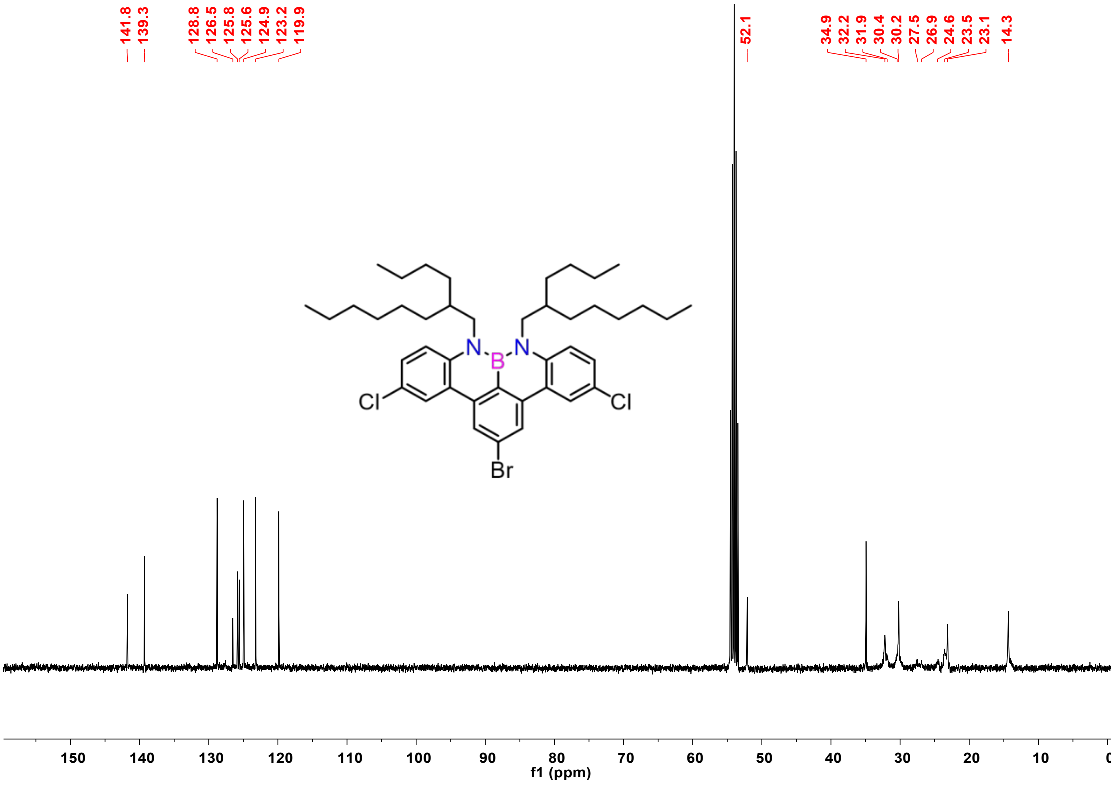

Figure S42. ^{13}C NMR spectrum of **5** in CD_2Cl_2 (101 MHz, 298 K).

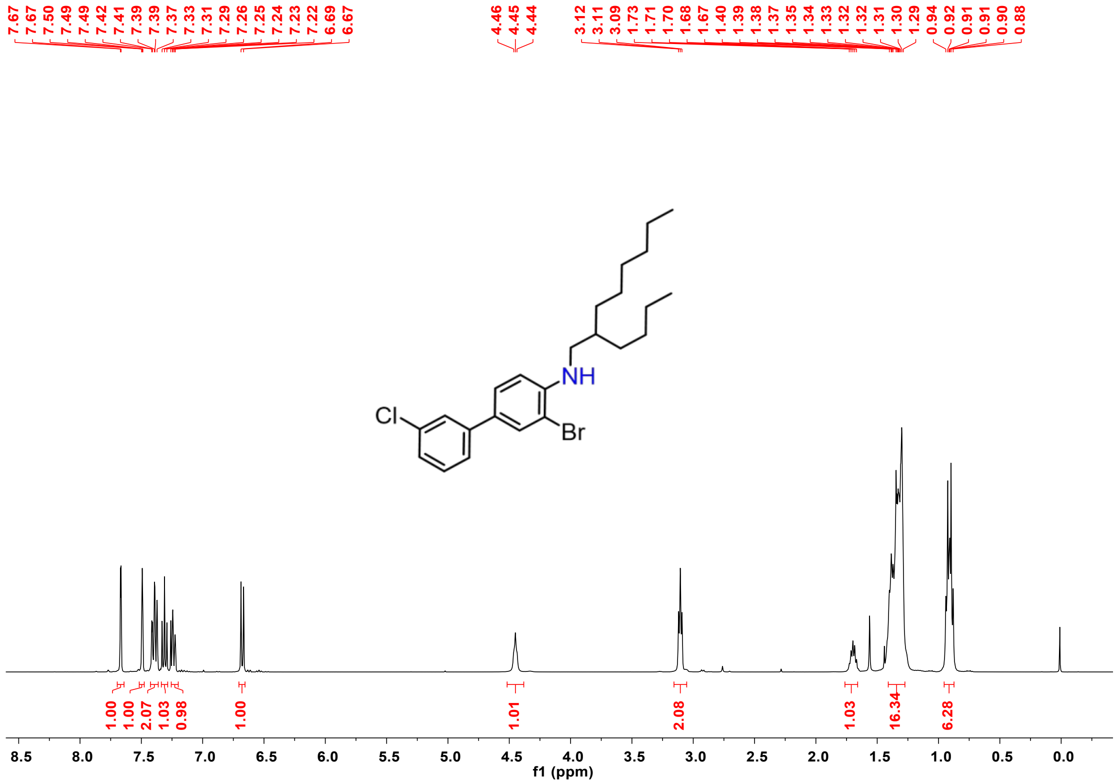

Figure S43. ^{1}H NMR spectrum of **7** in $CDCl_3$ (400 MHz, 298 K).

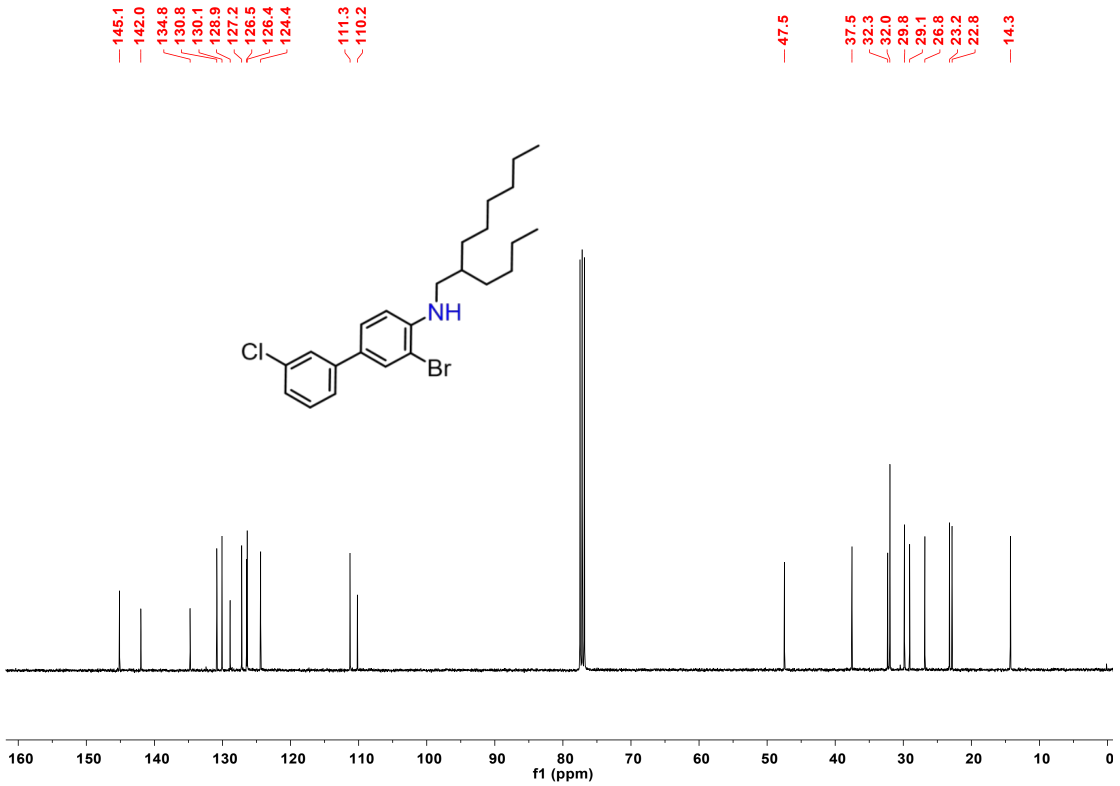

Figure S44. ^{13}C NMR spectrum of **7** in $CDCl_3$ (101 MHz, 298 K).

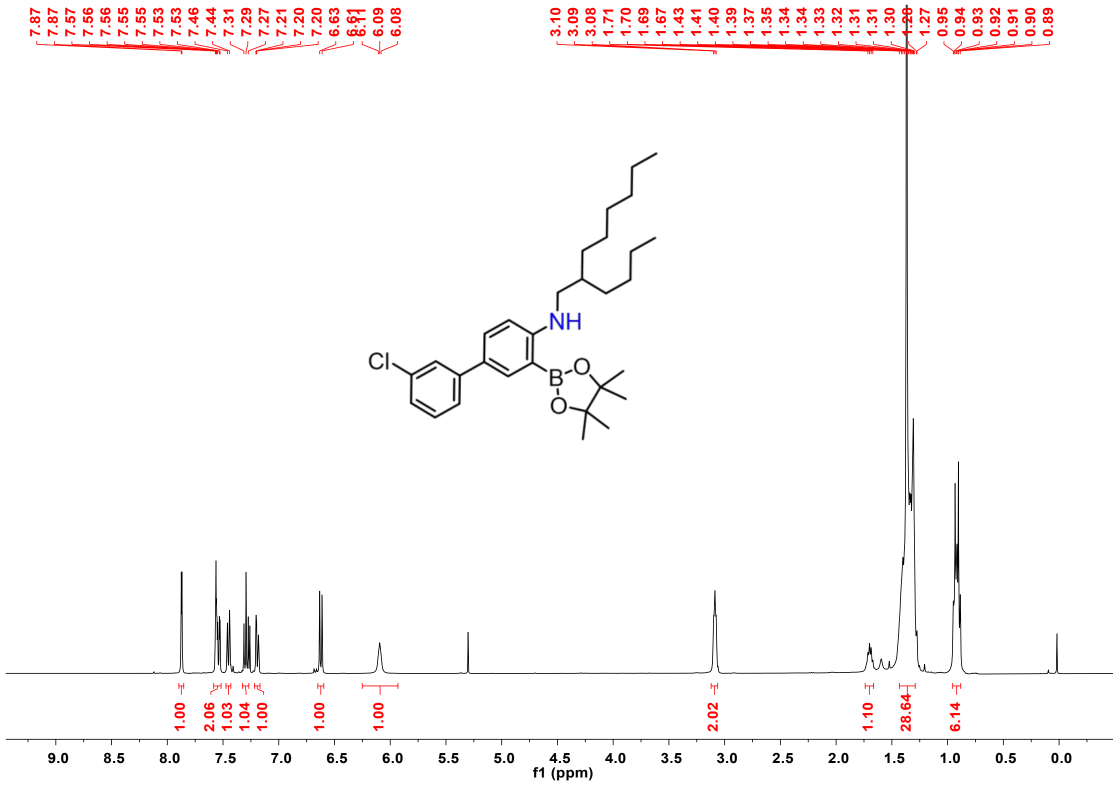

Figure S45. ^{1}H NMR spectrum of **8** in $CDCl_3$ (400 MHz, 298 K).

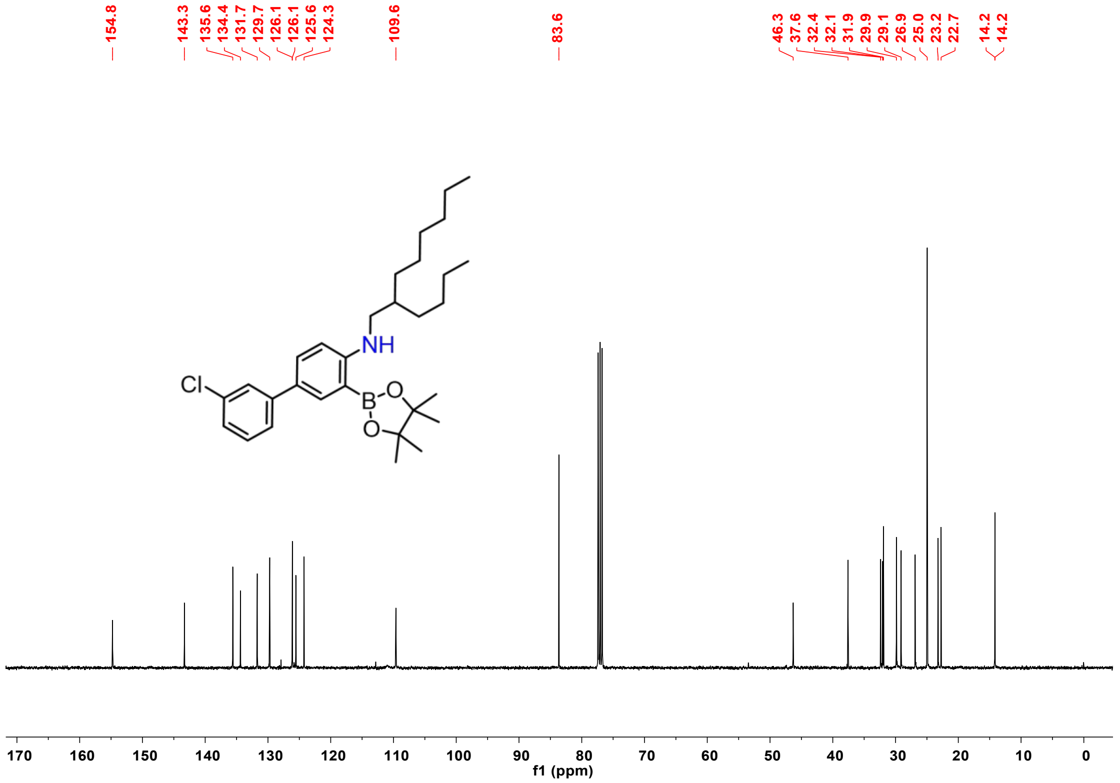

Figure S46. ^{13}C NMR spectrum of **8** in $CDCl_3$ (101 MHz, 298 K).

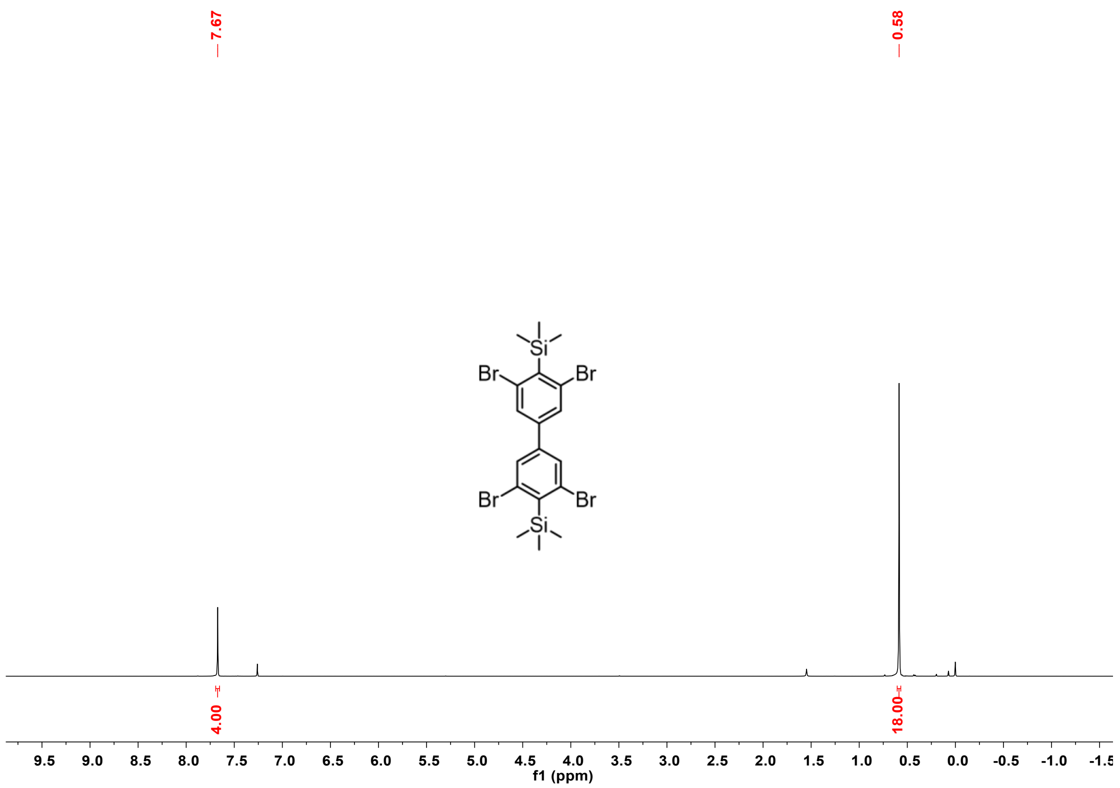

Figure S47. ^{1}H NMR spectrum of **10** in $CDCl_3$ (400 MHz, 298 K).

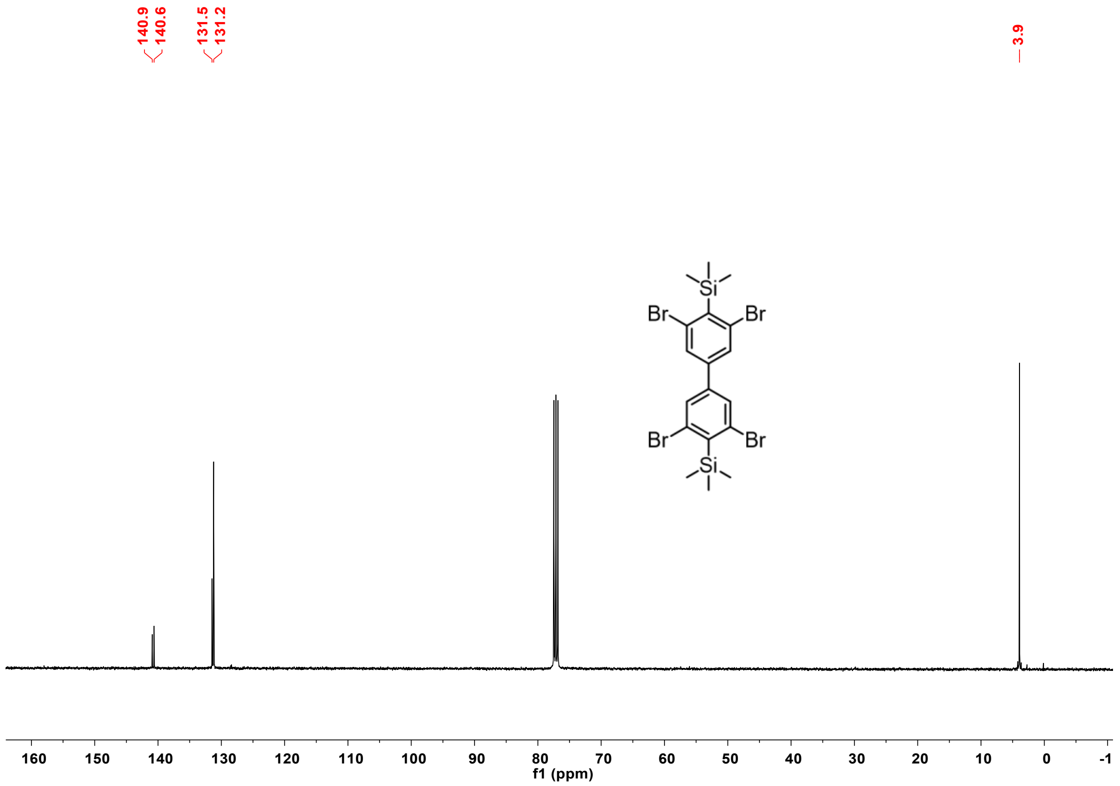

Figure S48. ^{13}C NMR spectrum of **10** in $CDCl_3$ (101 MHz, 298 K).

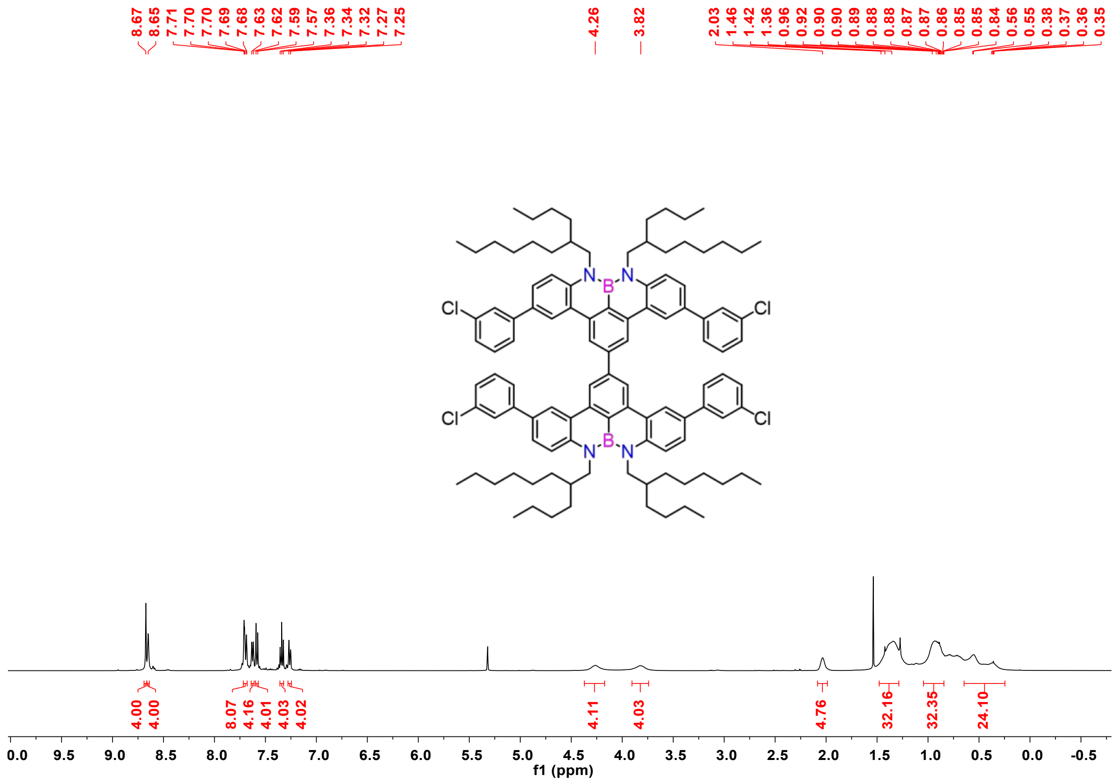

Figure S49. ^{1}H NMR spectrum of **12** in CD_2Cl_2 (500 MHz, 298 K).

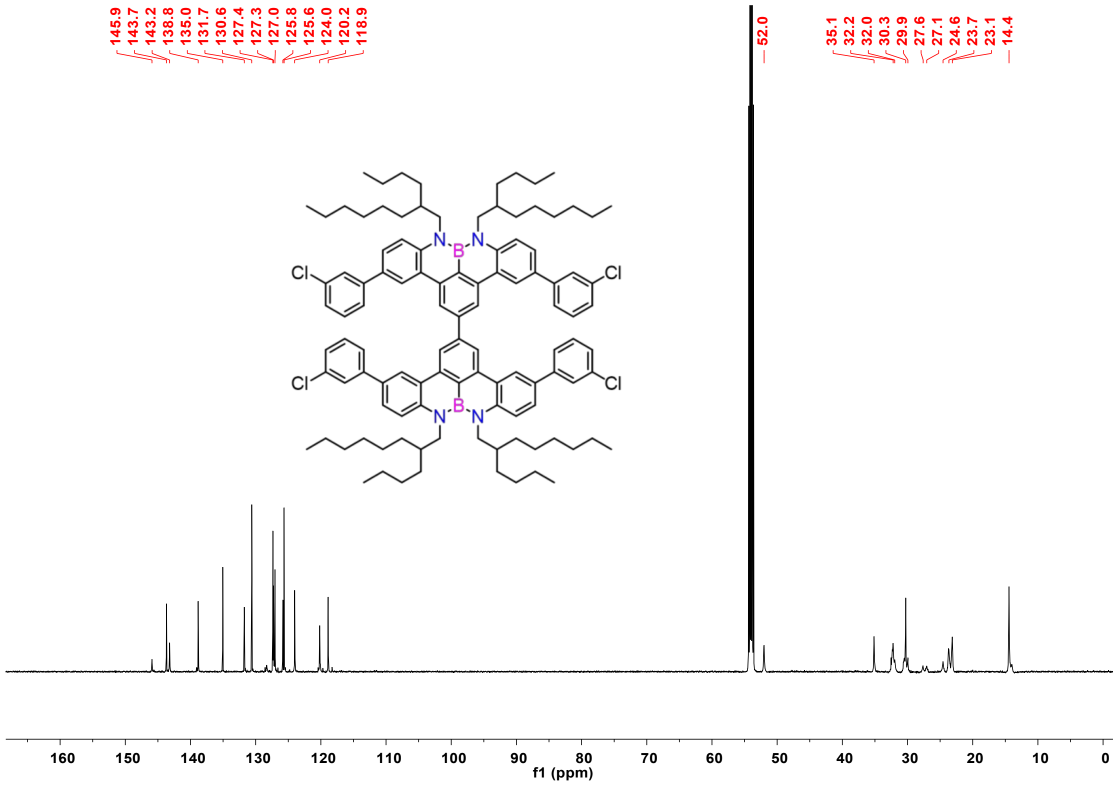

Figure S50. ^{13}C NMR spectrum of **12** in CD_2Cl_2 (151 MHz, 298 K).

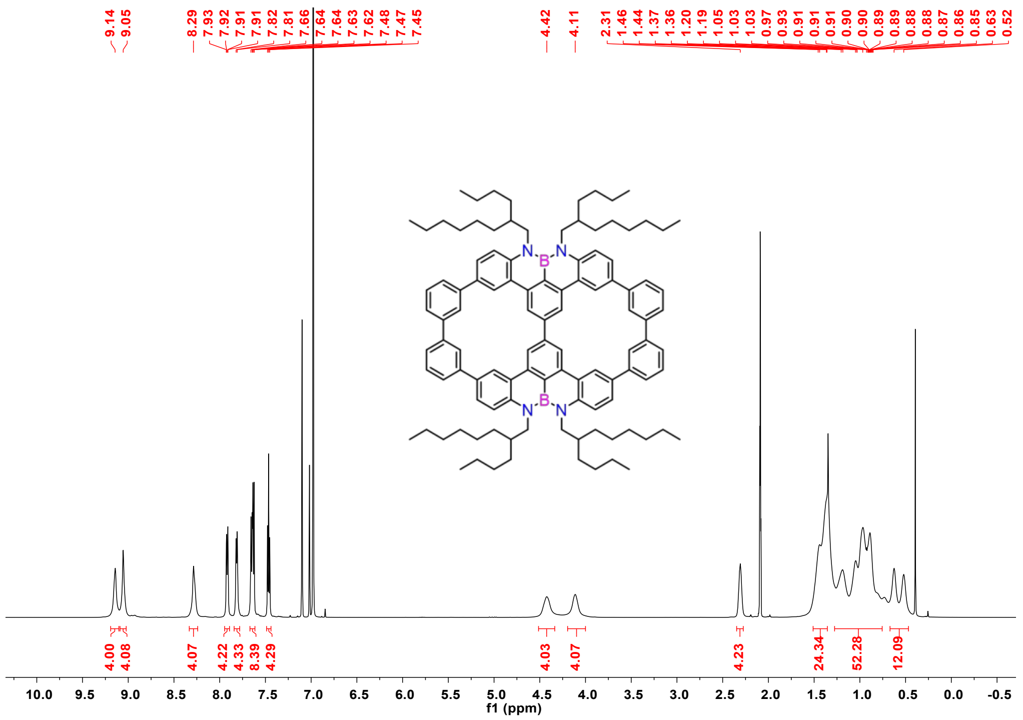

Figure S51. ^{1}H NMR spectrum of **2PNG** in Toluene-d_8 (600 MHz, 298 K).

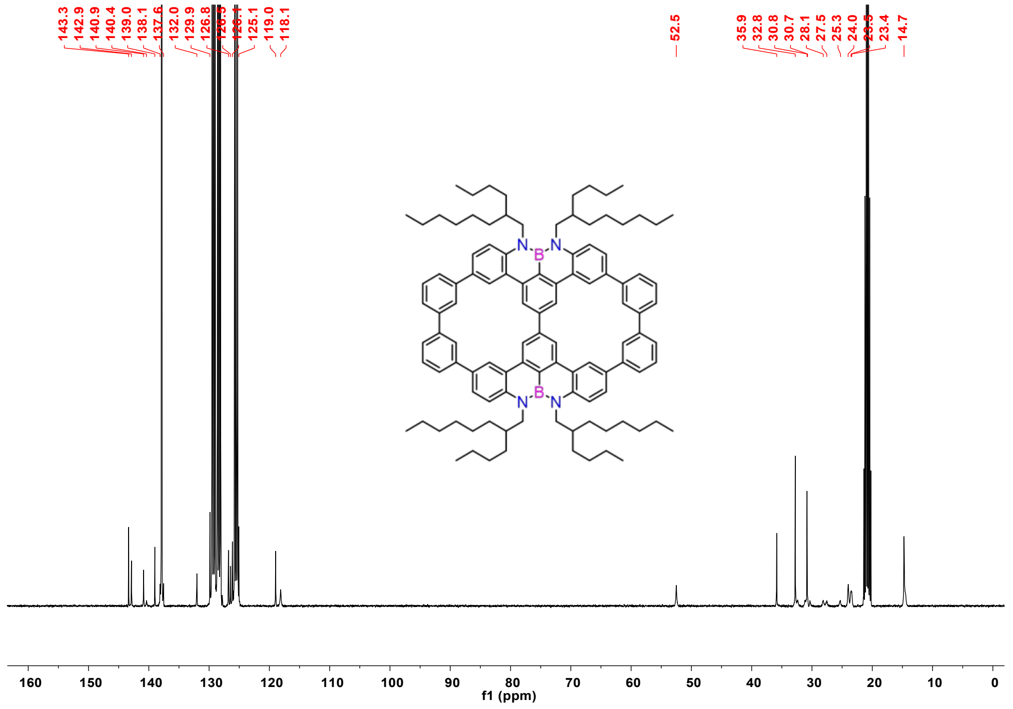

Figure S52. ^{13}C NMR spectrum of **2PNG** in Toluene-d_8 (101 MHz, 298 K).

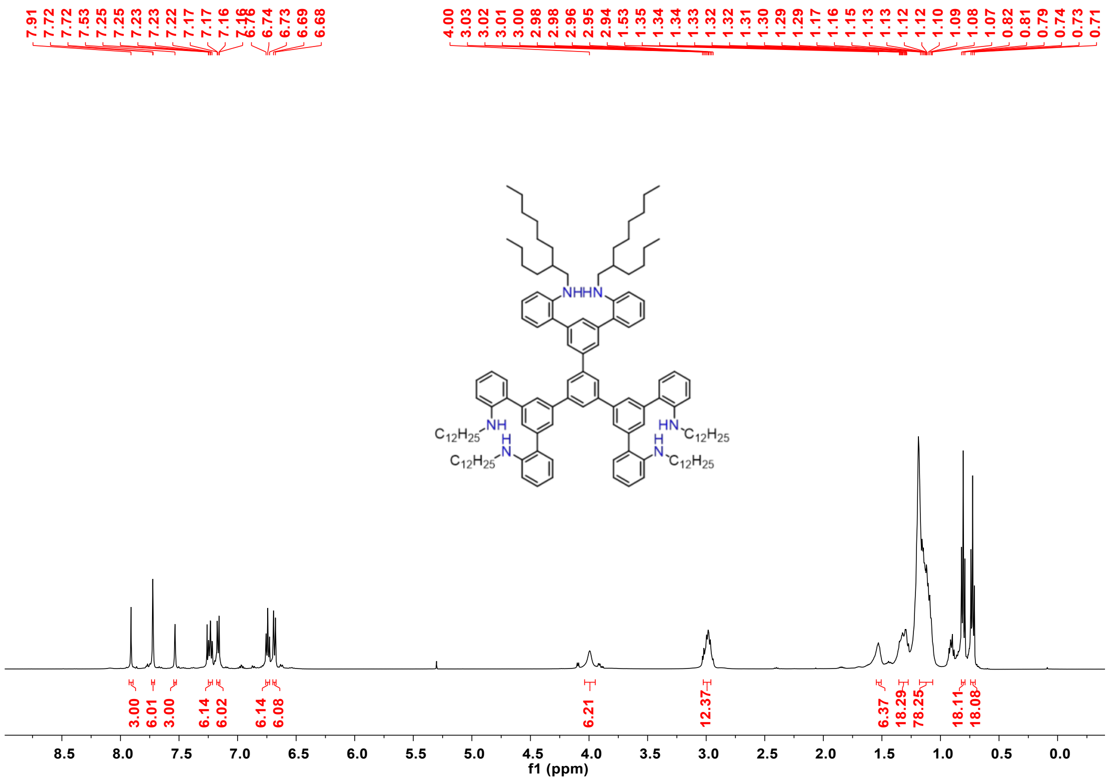

Figure S53. ^{1}H NMR spectrum of **14** in $CDCl_3$ (500 MHz, 298 K).

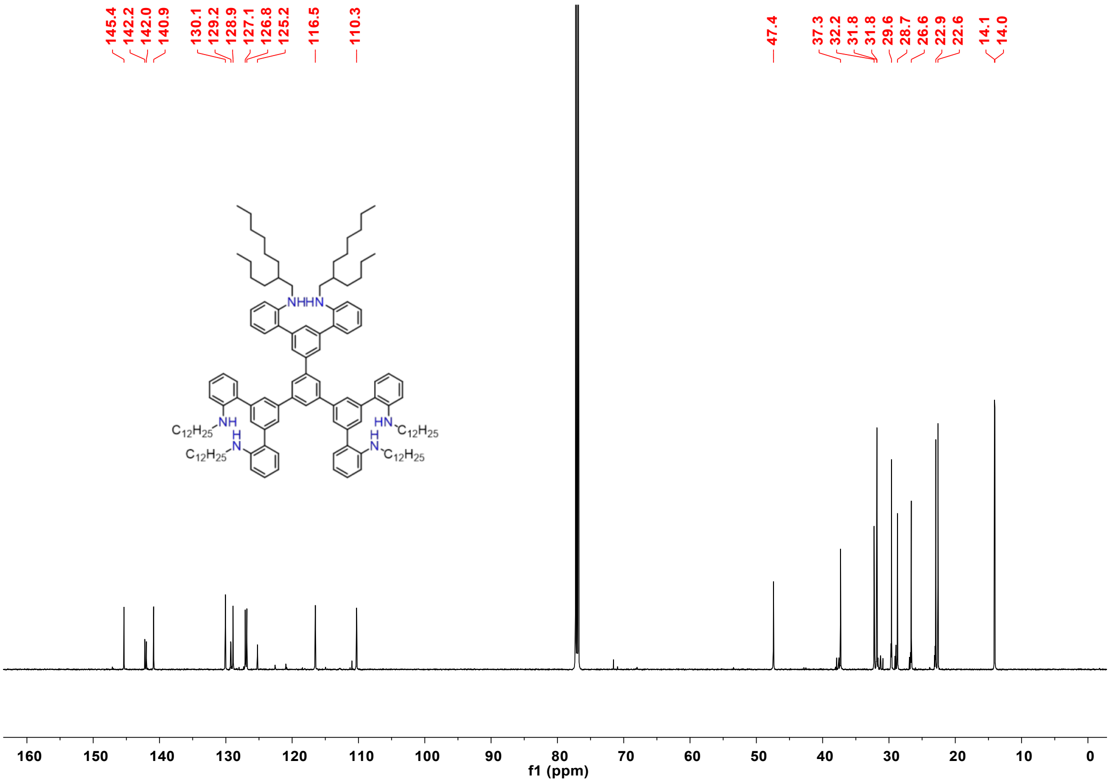

Figure S54. ^{13}C NMR spectrum of **14** in $CDCl_3$ (151 MHz, 298 K).

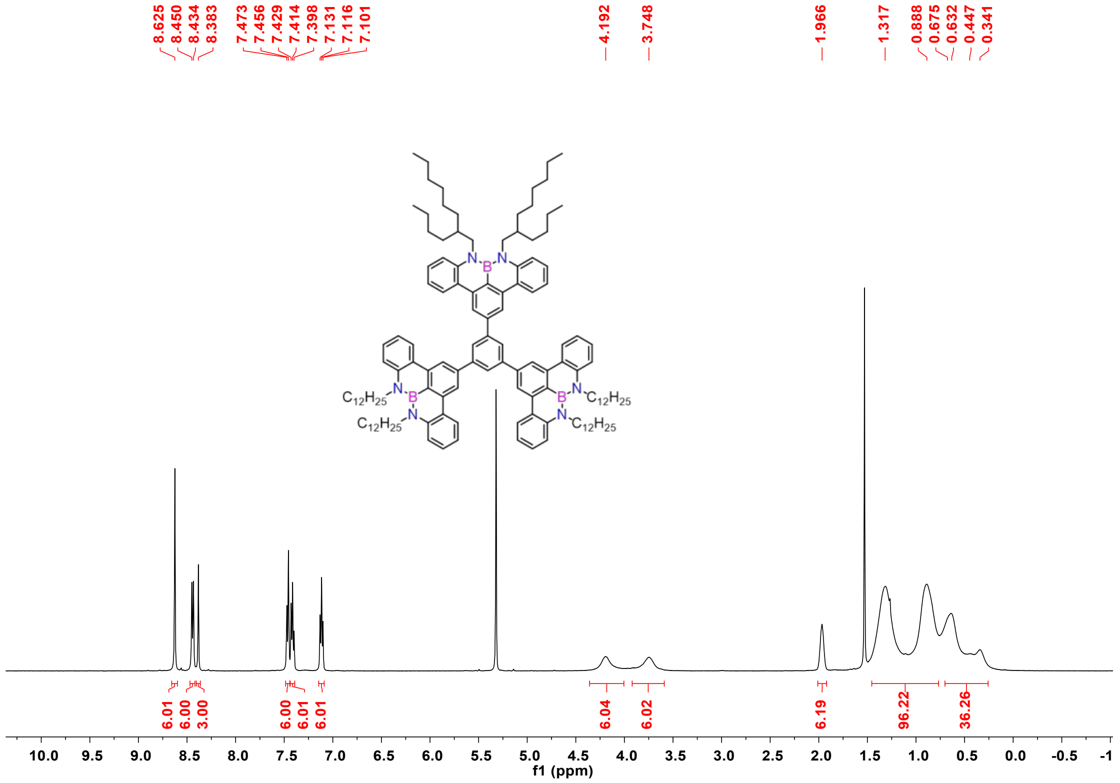

Figure S55. ^{1}H NMR spectrum of **15** in CD_2Cl_2 (500 MHz, 298 K).

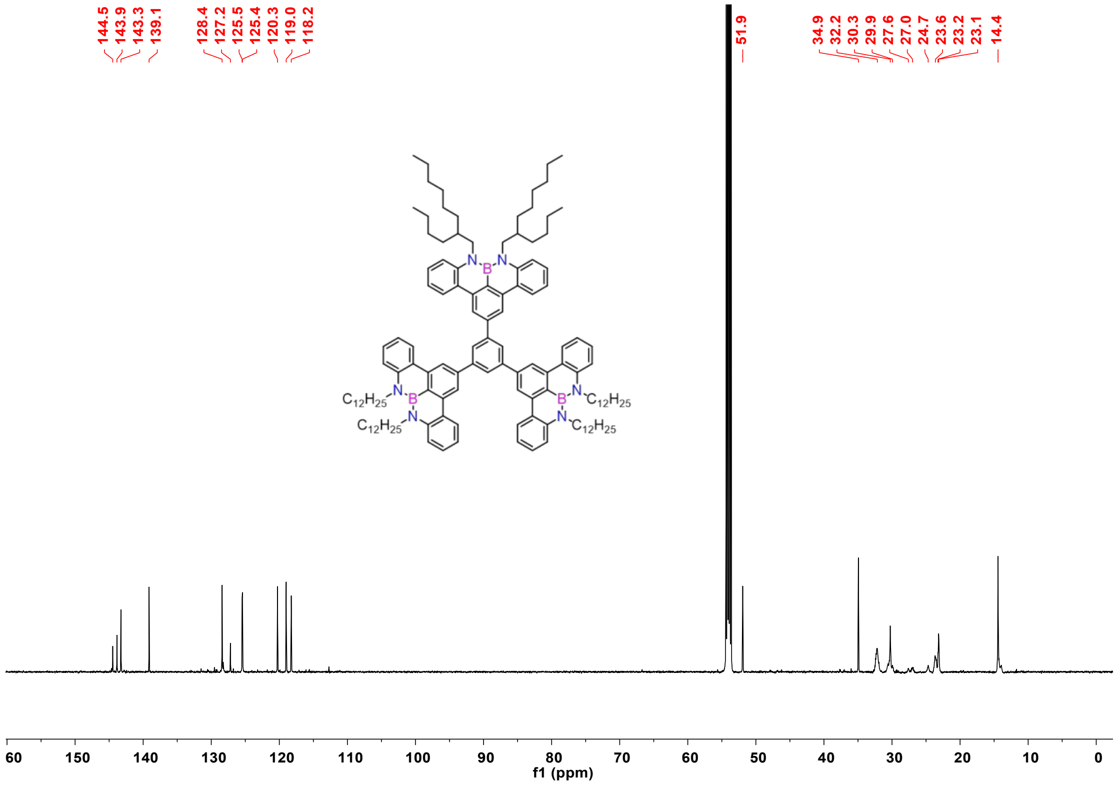

Figure S56. ^{13}C NMR spectrum of **15** in CD_2Cl_2 (151 MHz, 298 K).

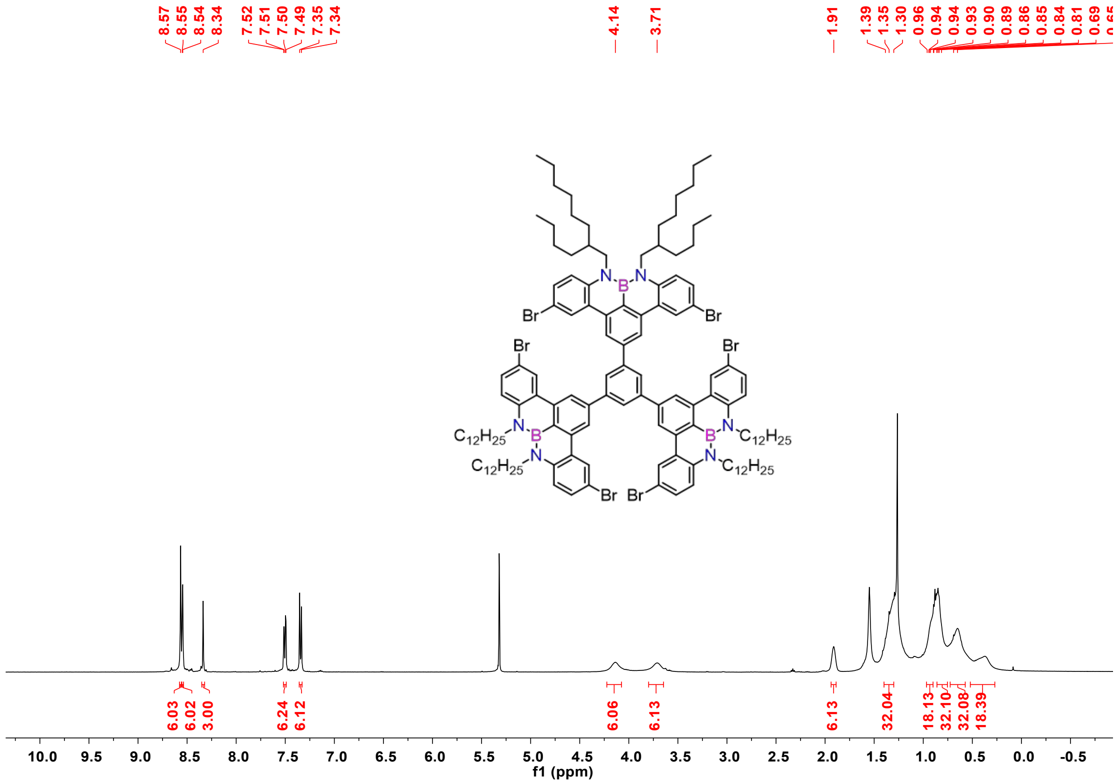

Figure S57. ^{1}H NMR spectrum of **16** in CD_2Cl_2 (500 MHz, 298 K).

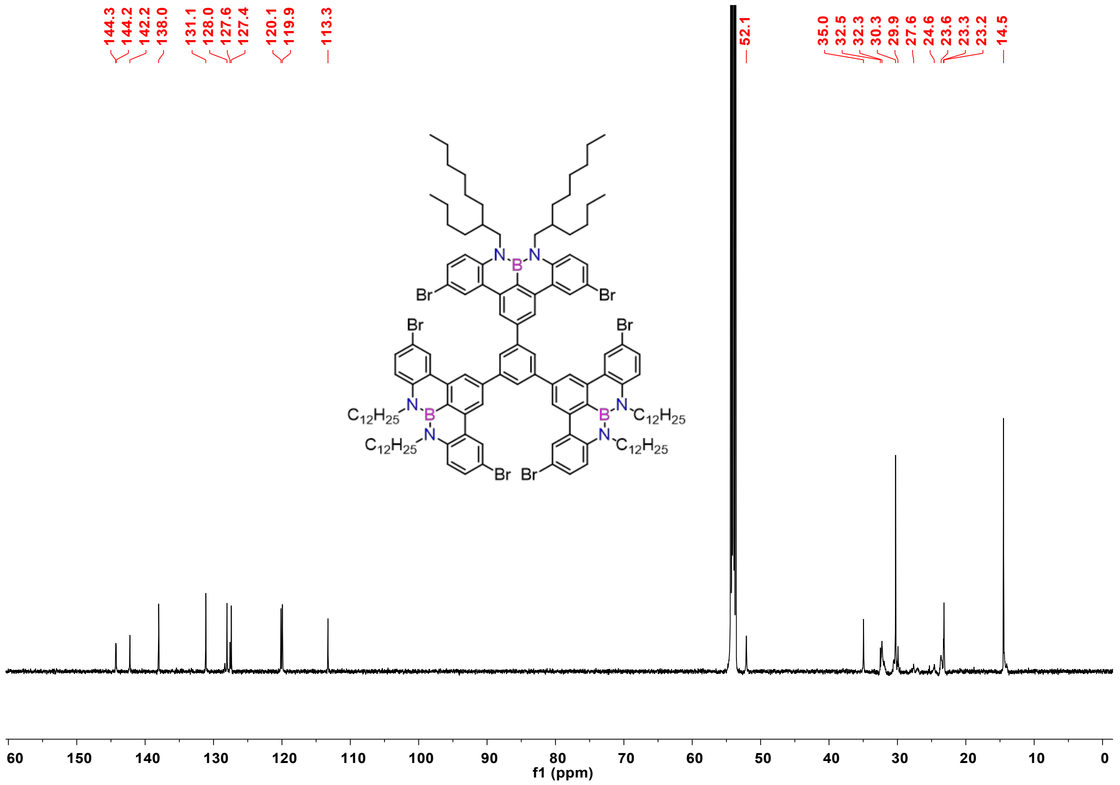

Figure S58. ^{13}C NMR spectrum of **16** in CD_2Cl_2 (151 MHz, 298 K).

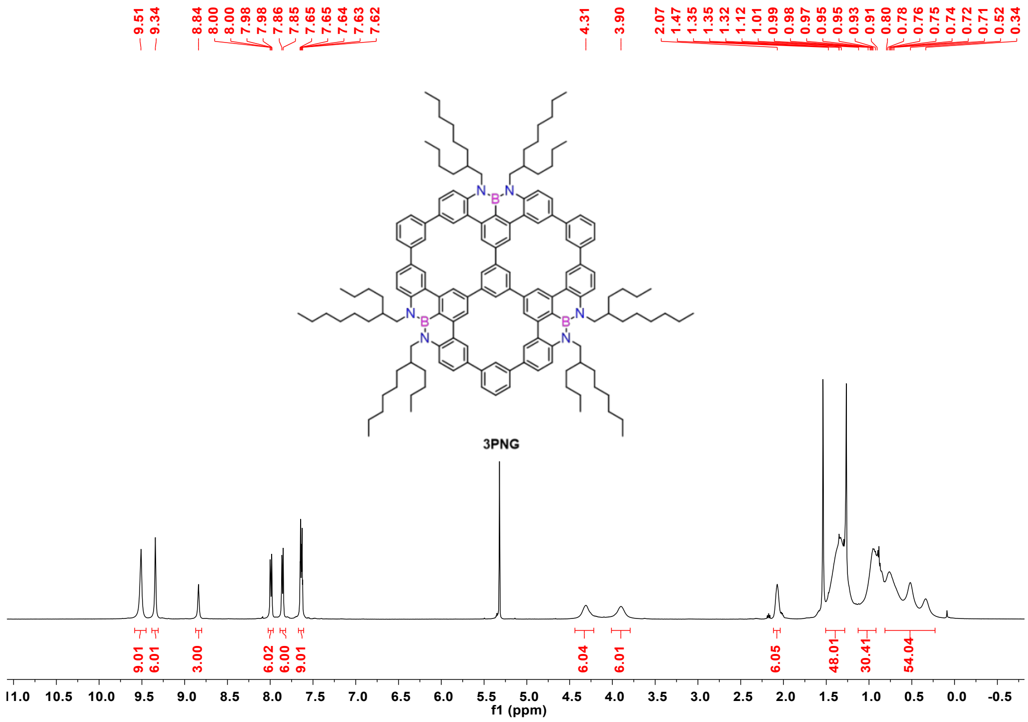

Figure S59. ^{1}H NMR spectrum of **3PNG** in CD_2Cl_2 (500 MHz, 298 K).

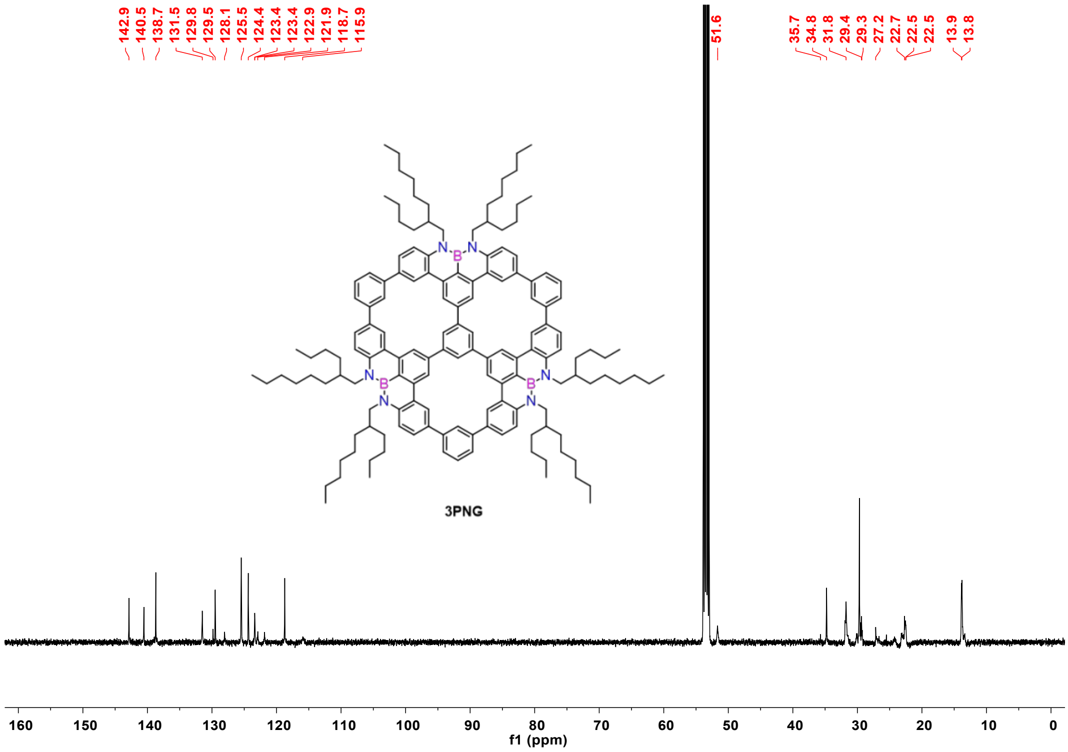

Figure S60. ^{13}C NMR spectrum of **3PNG** in CD_2Cl_2 (126 MHz, 298 K).

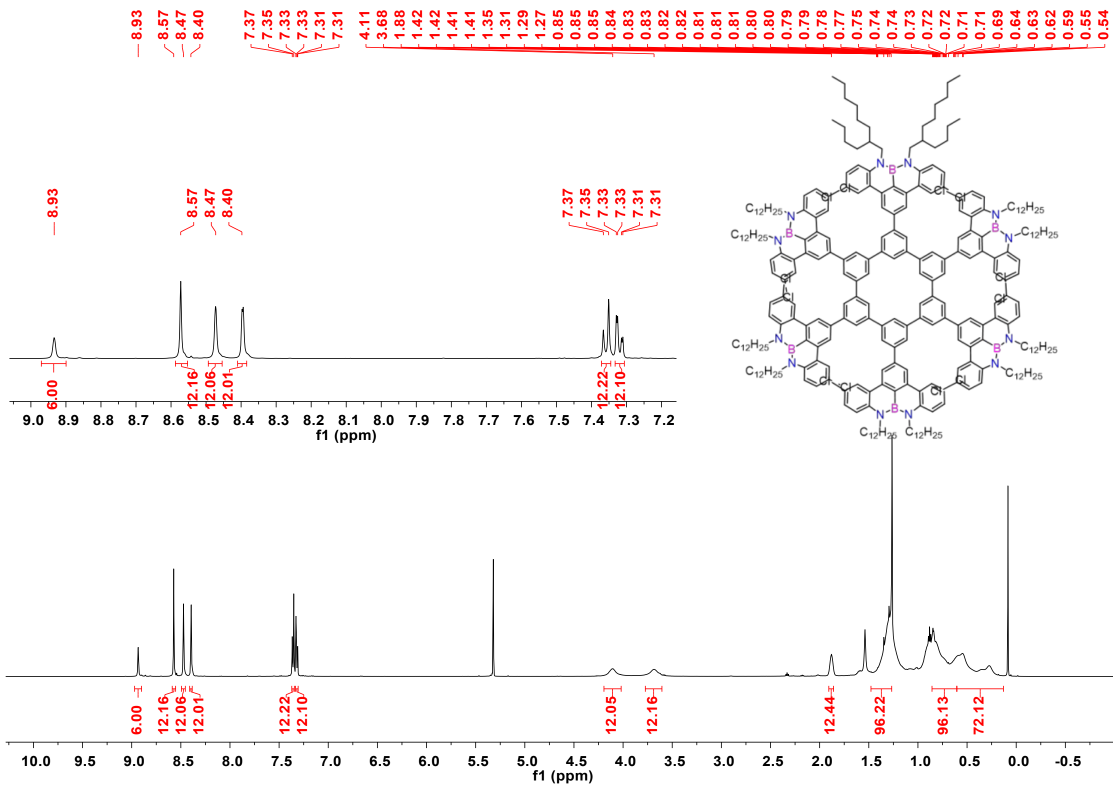

Figure S61. ^{1}H NMR spectrum of **18** in CD_2Cl_2 (600 MHz, 298 K).

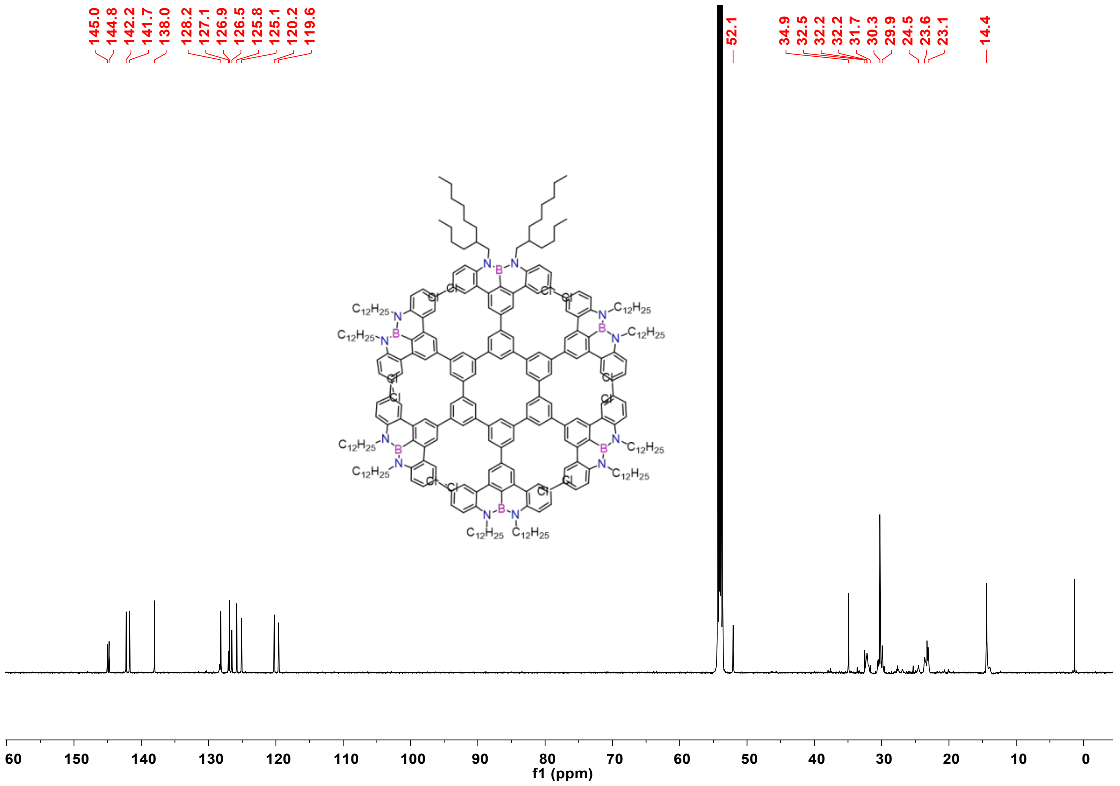

Figure S62. ^{13}C NMR spectrum of **18** in CD_2Cl_2 (151 MHz, 298 K).

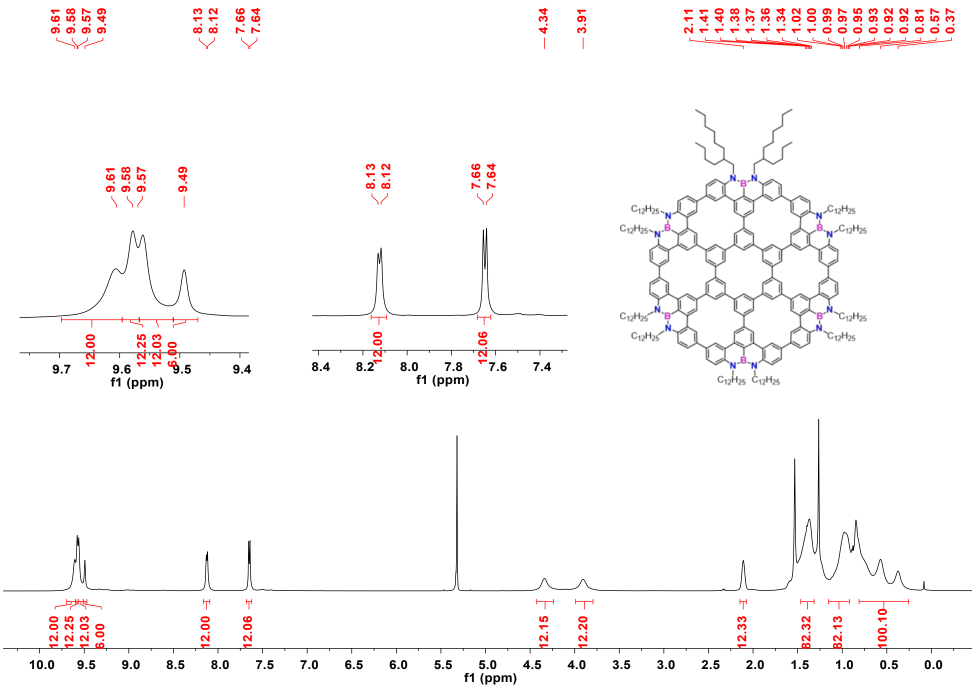

Figure S63. ^{1}H NMR spectrum of **7PNG** in CD_2Cl_2 (600 MHz, 298 K).

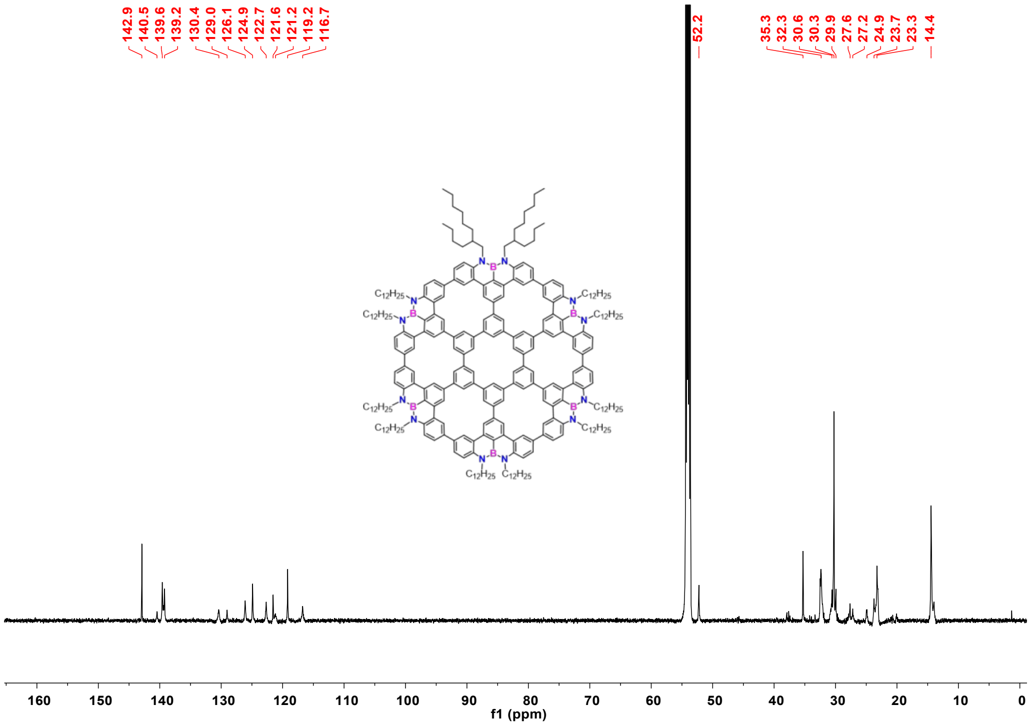

Figure S64. ^{13}C NMR spectrum of **7PNG** in CD_2Cl_2 (151 MHz, 298 K).

8. High-resolution mass spectrometry

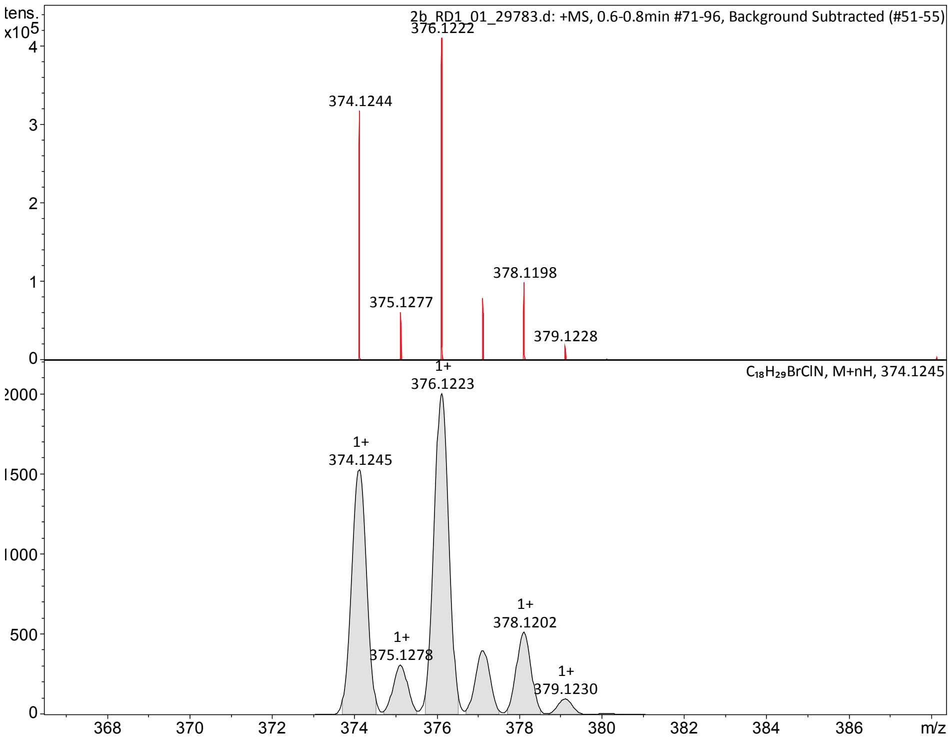

Figure S65. High resolution ESI mass spectrum of compound **2b**.

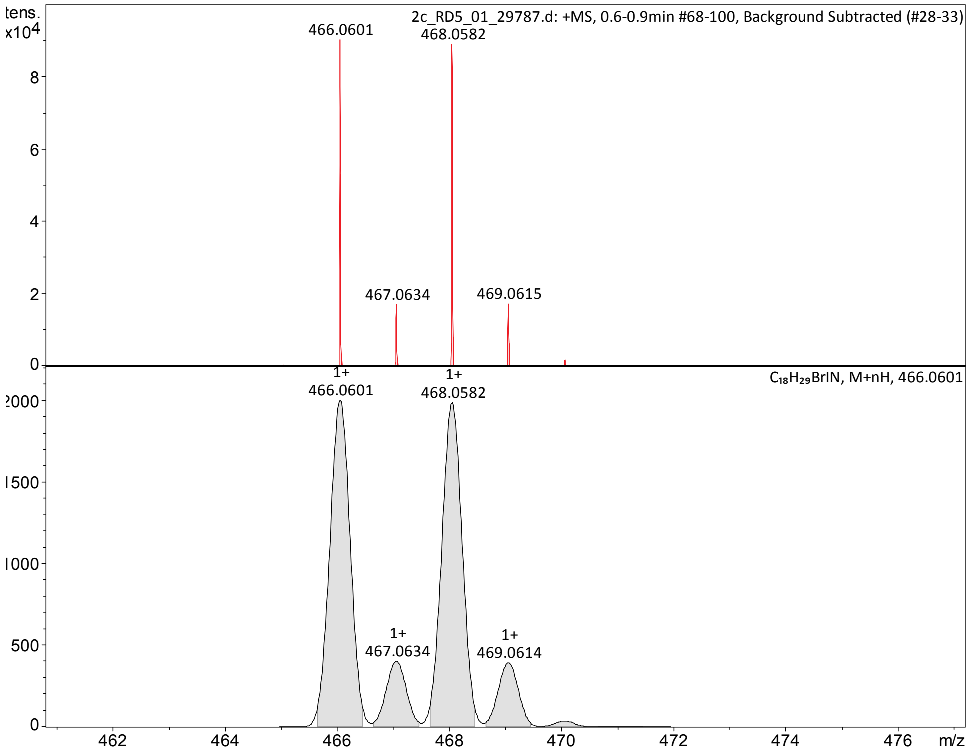

Figure S66. High-resolution ESI mass spectrum of compound **2c**.

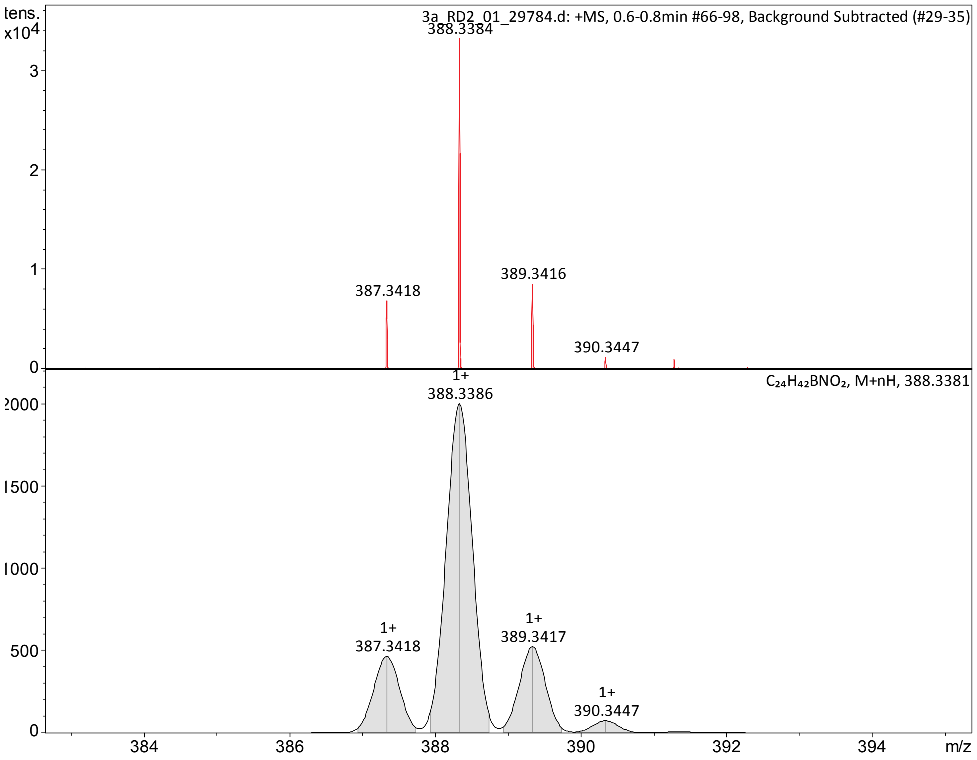

Figure S67. High resolution ESI mass spectrum of compound **3a**.

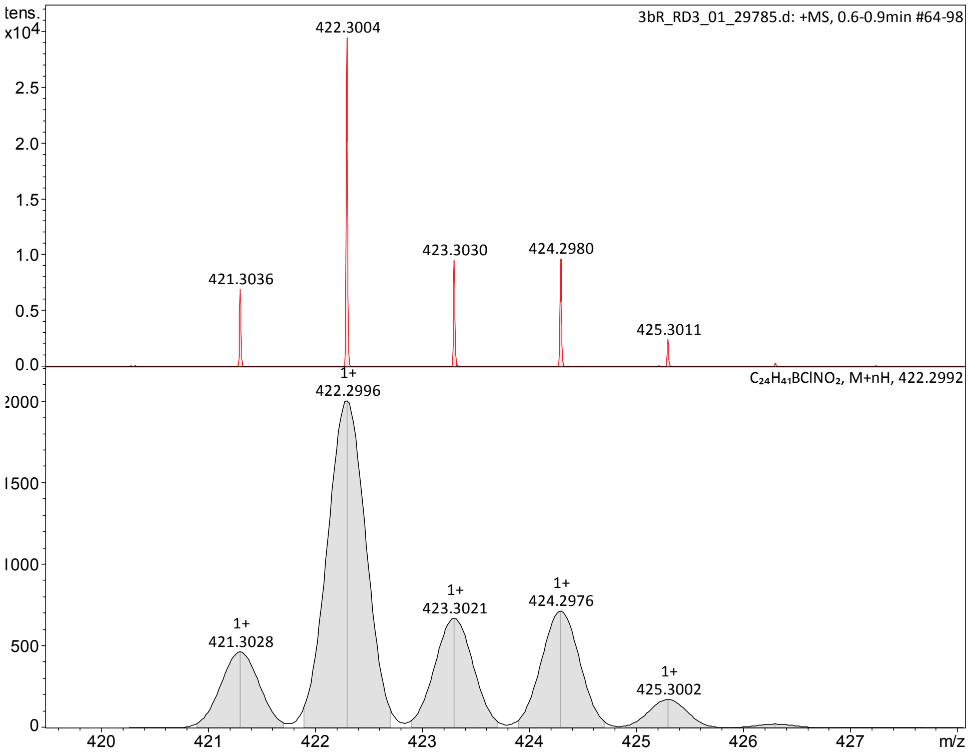

Figure S68. High resolution ESI mass spectrum of compound **3b**.

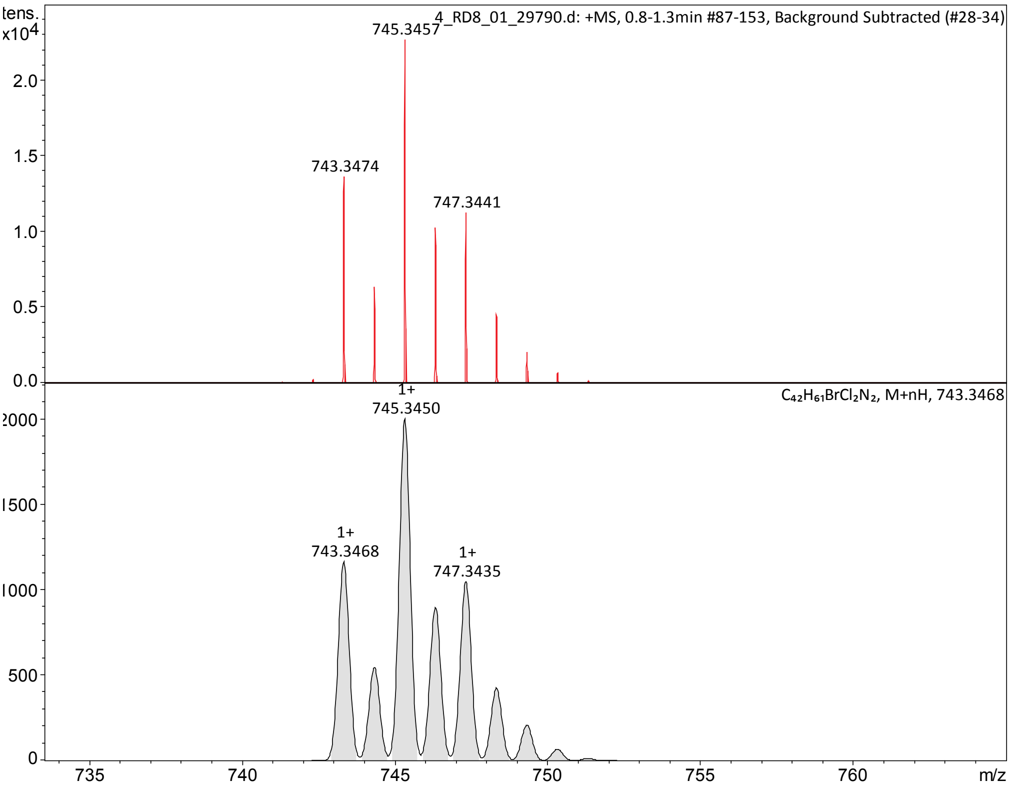

Figure S69. High resolution ESI mass spectrum of compound **4**.

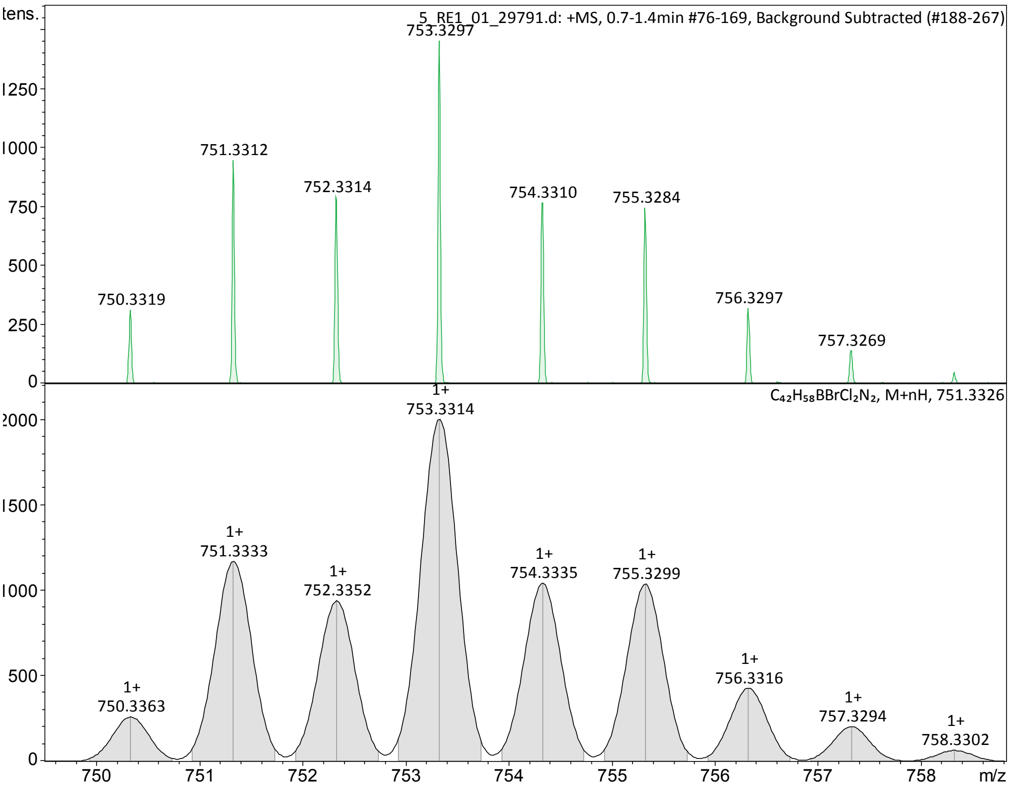

Figure S70. High resolution ESI mass spectrum of compound **5**.

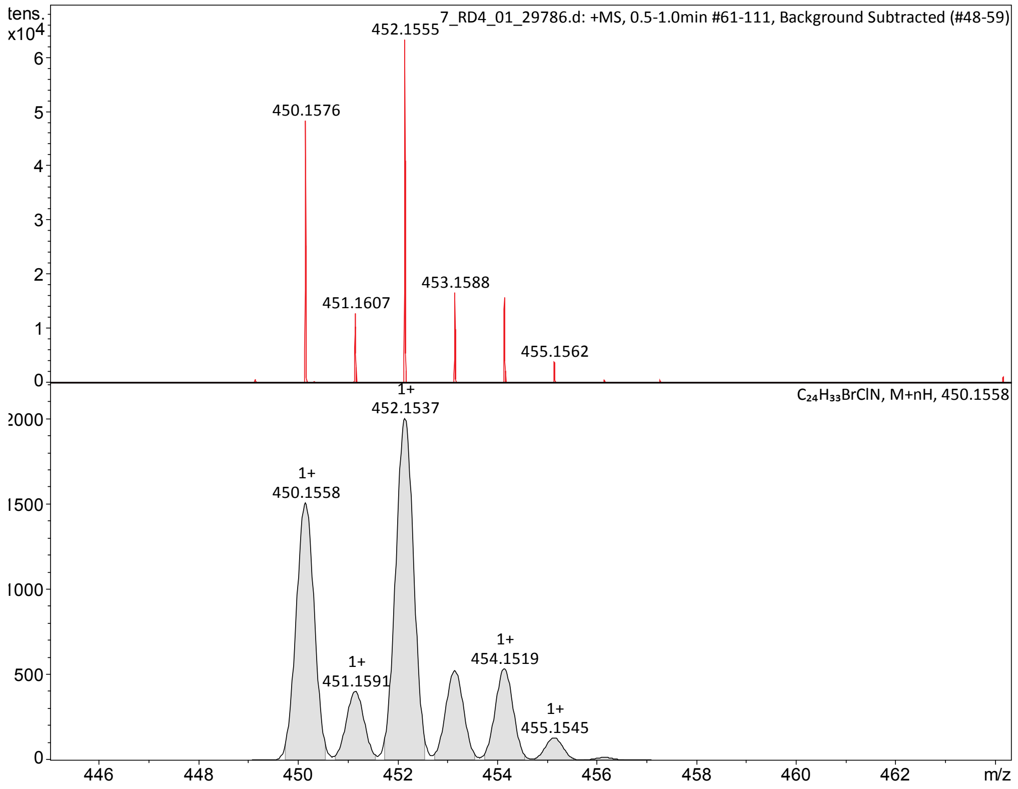

Figure S71. High resolution ESI mass spectrum of compound **7**.

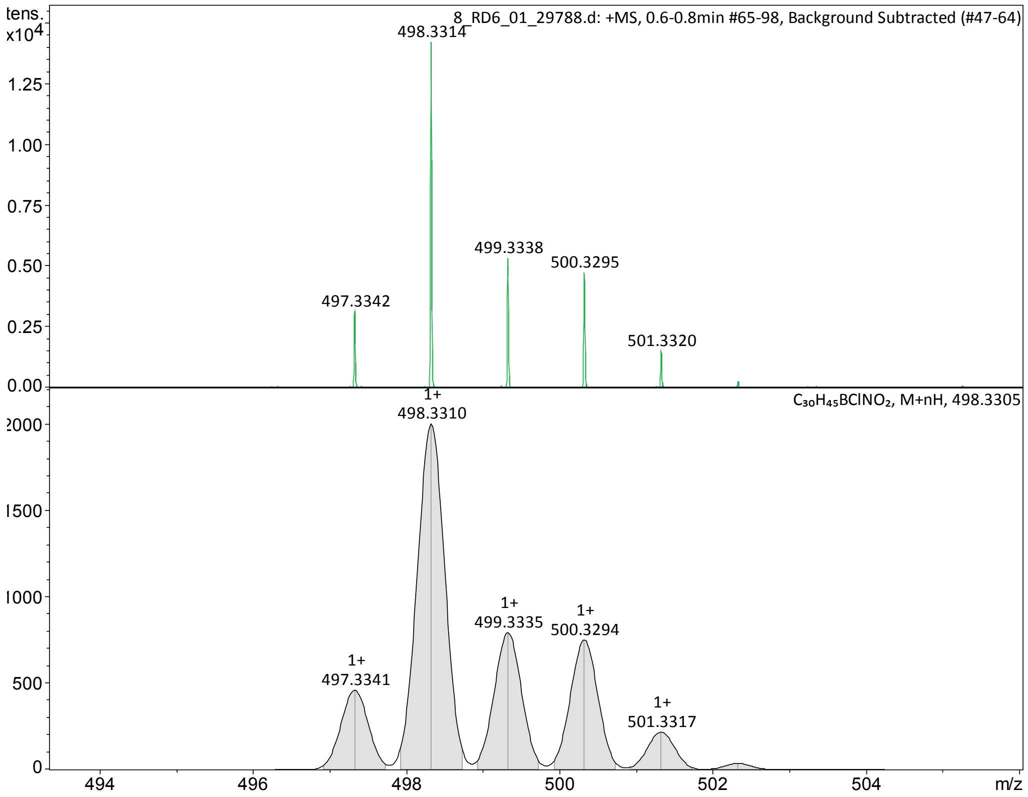

Figure S72. High resolution ESI mass spectrum of compound **8**.

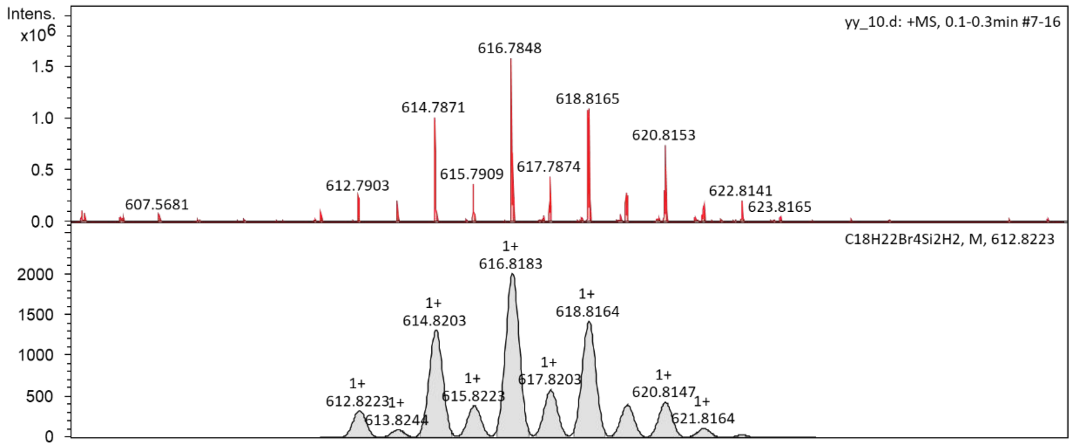

Figure S73. APCI mass spectrum of compound **10**.

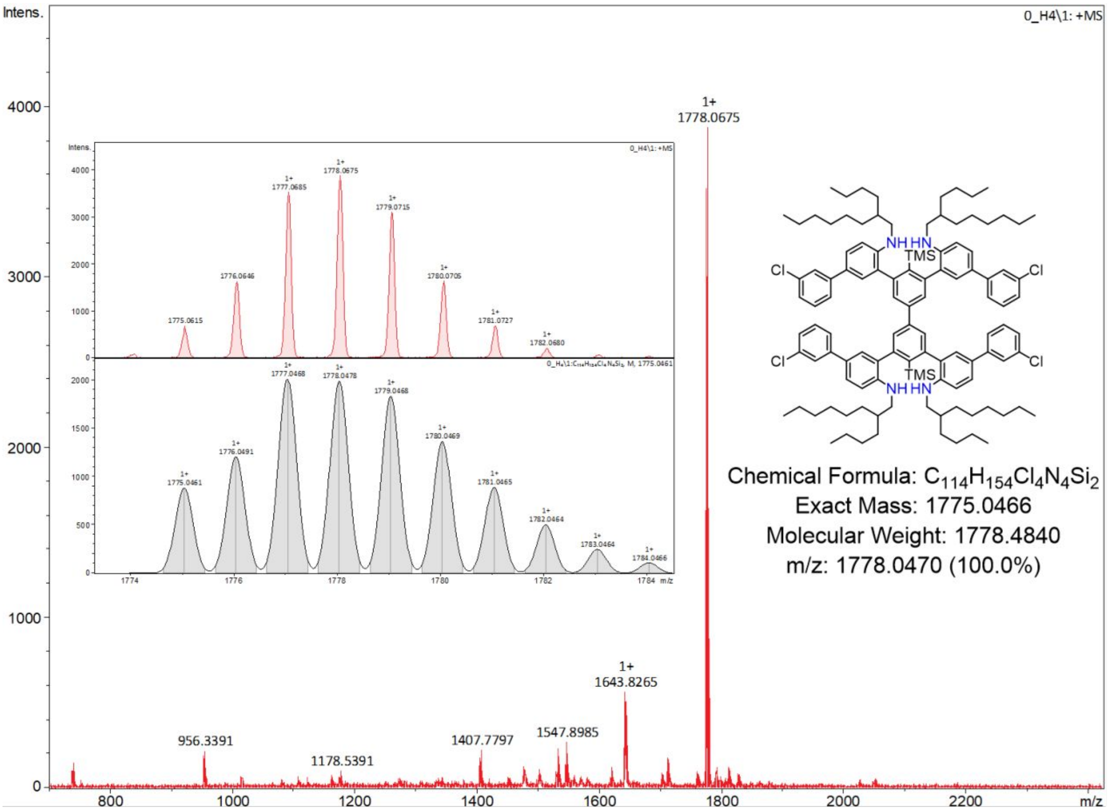

Figure S74. High resolution Maldi-tof-ms of compound **11**.

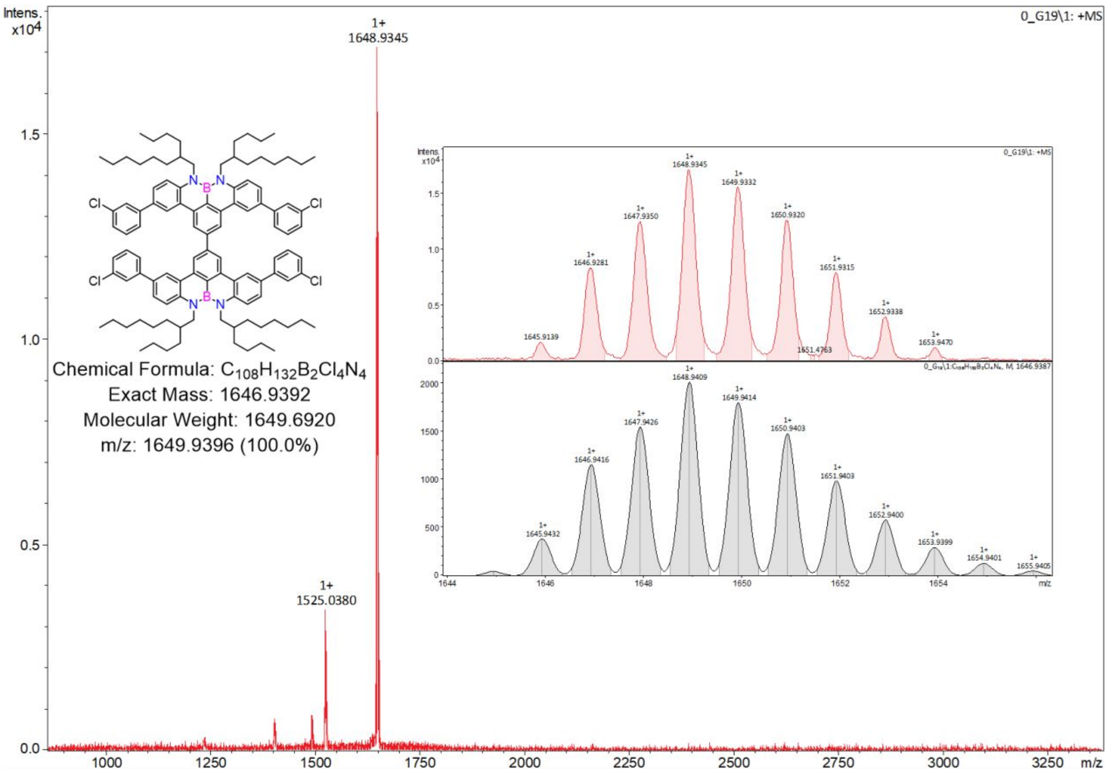

Figure S75. High resolution Maldi-tof-ms of compound **12**.

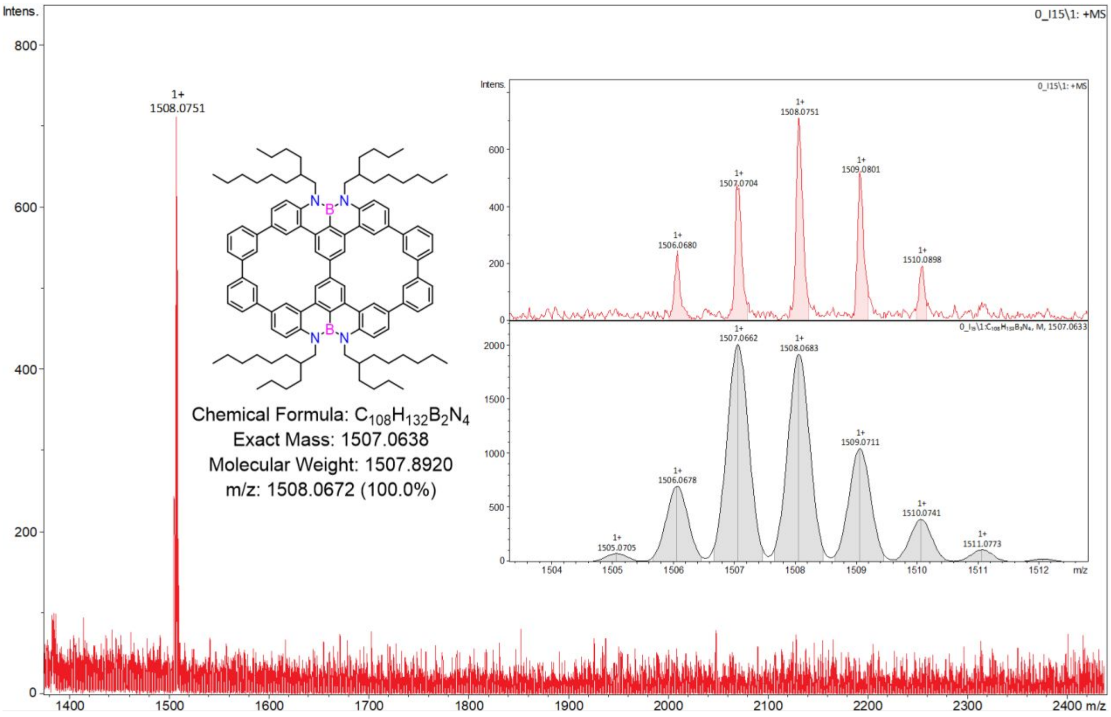

Figure S76. High resolution Maldi-tof-ms of compound **2PNG**.

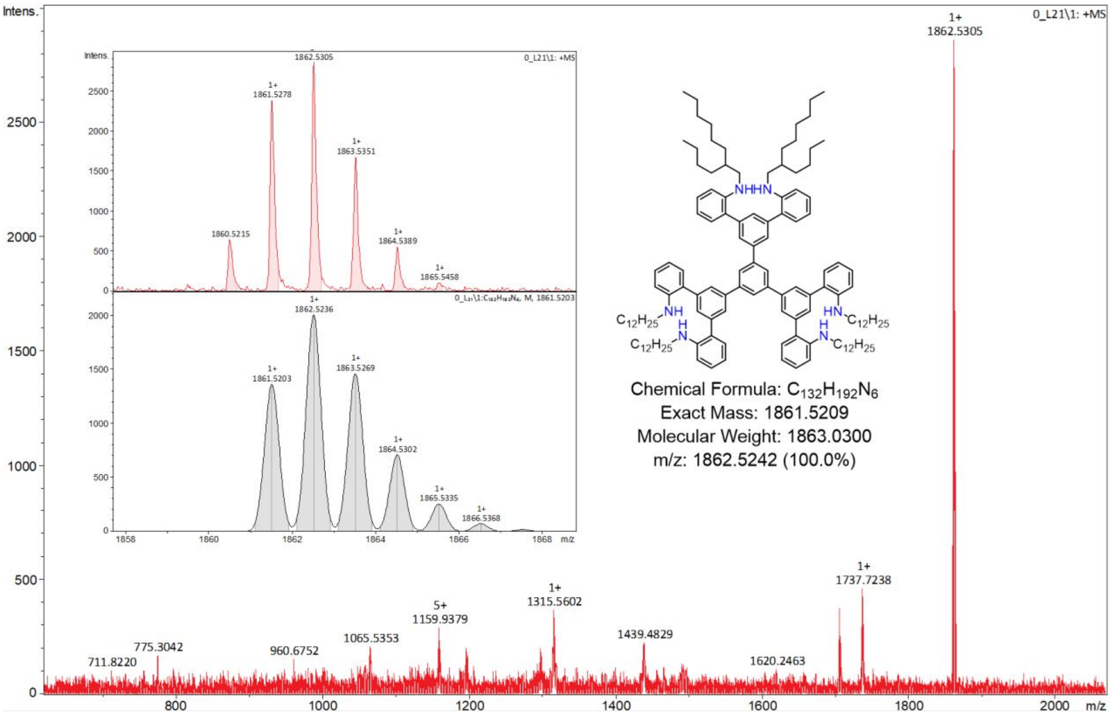

Figure S77. High resolution Maldi-tof-ms of compound **14**.

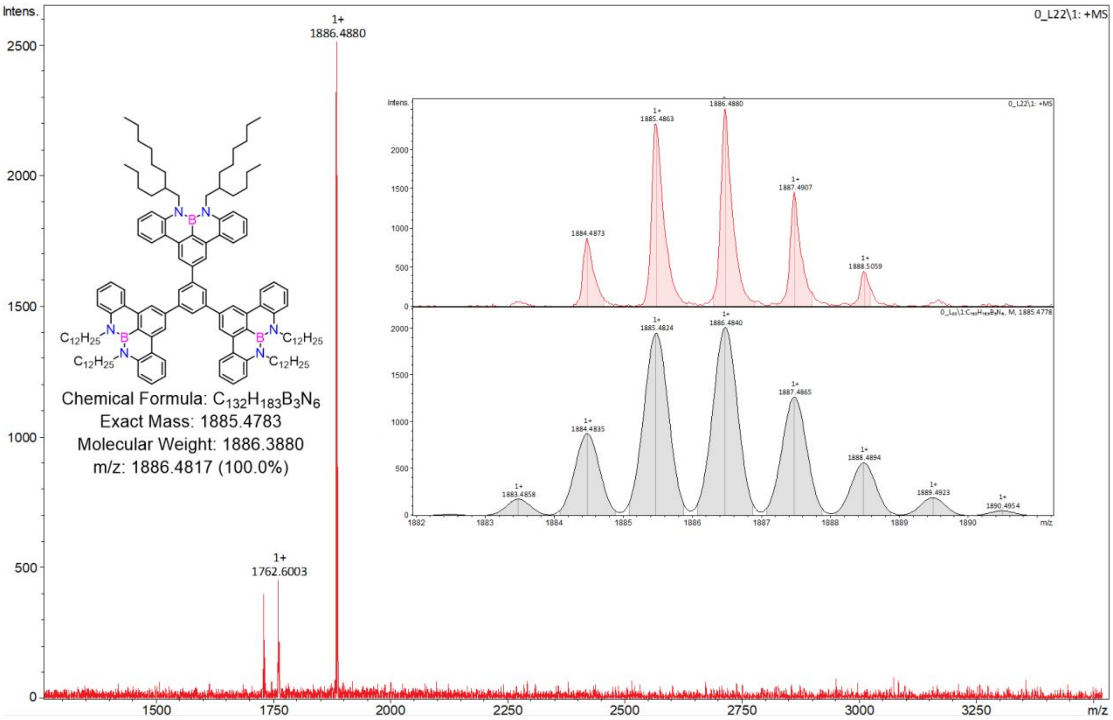

Figure S78. High resolution Maldi-tof-ms of compound **15**.

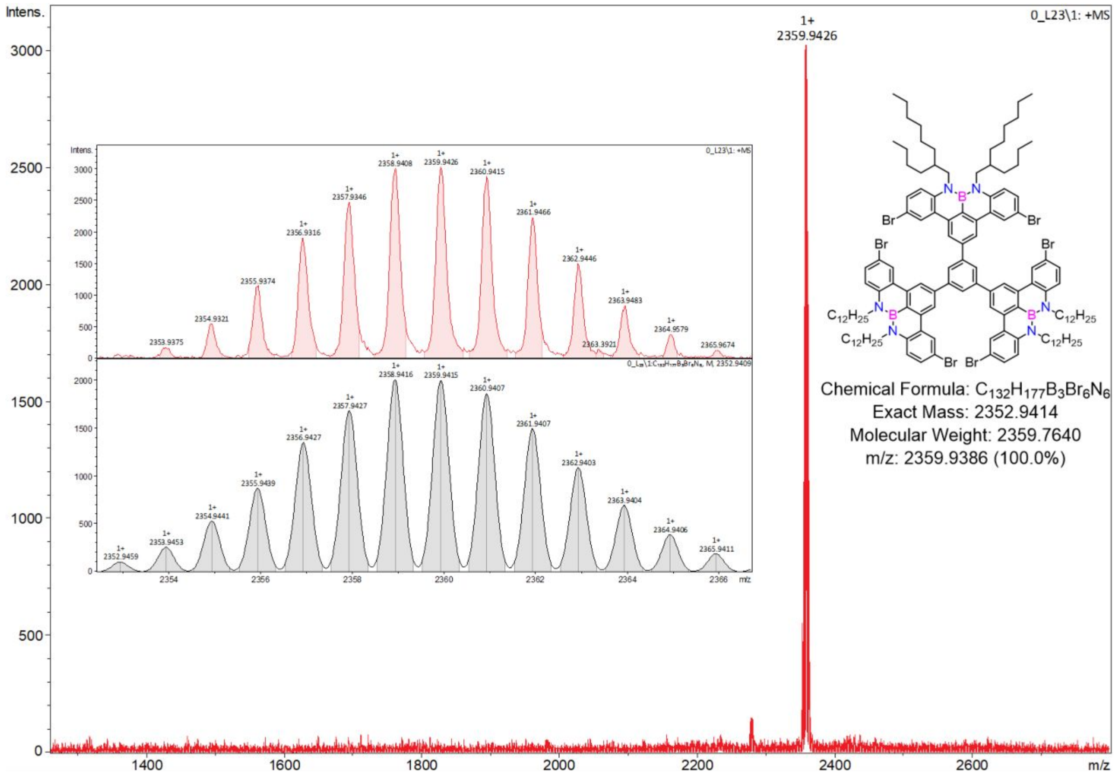

Figure S79. High resolution Maldi-tof-ms of compound **16**.

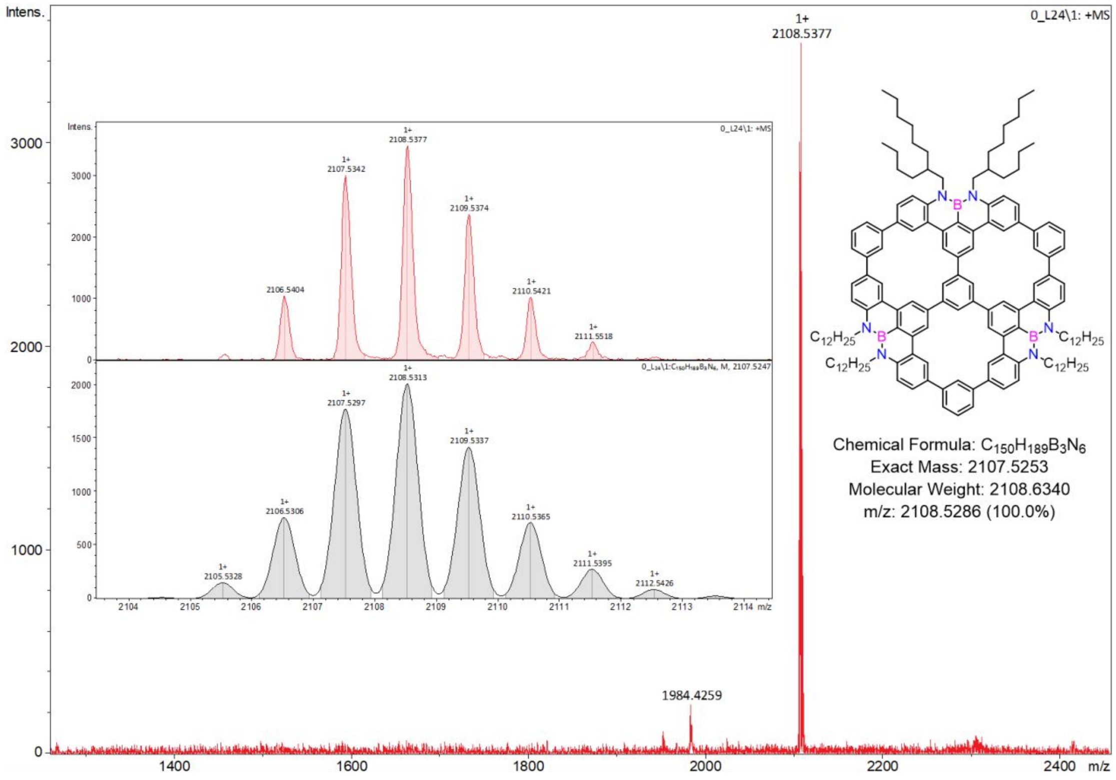

Figure S80. High resolution Maldi-tof-ms of **3PNG**.

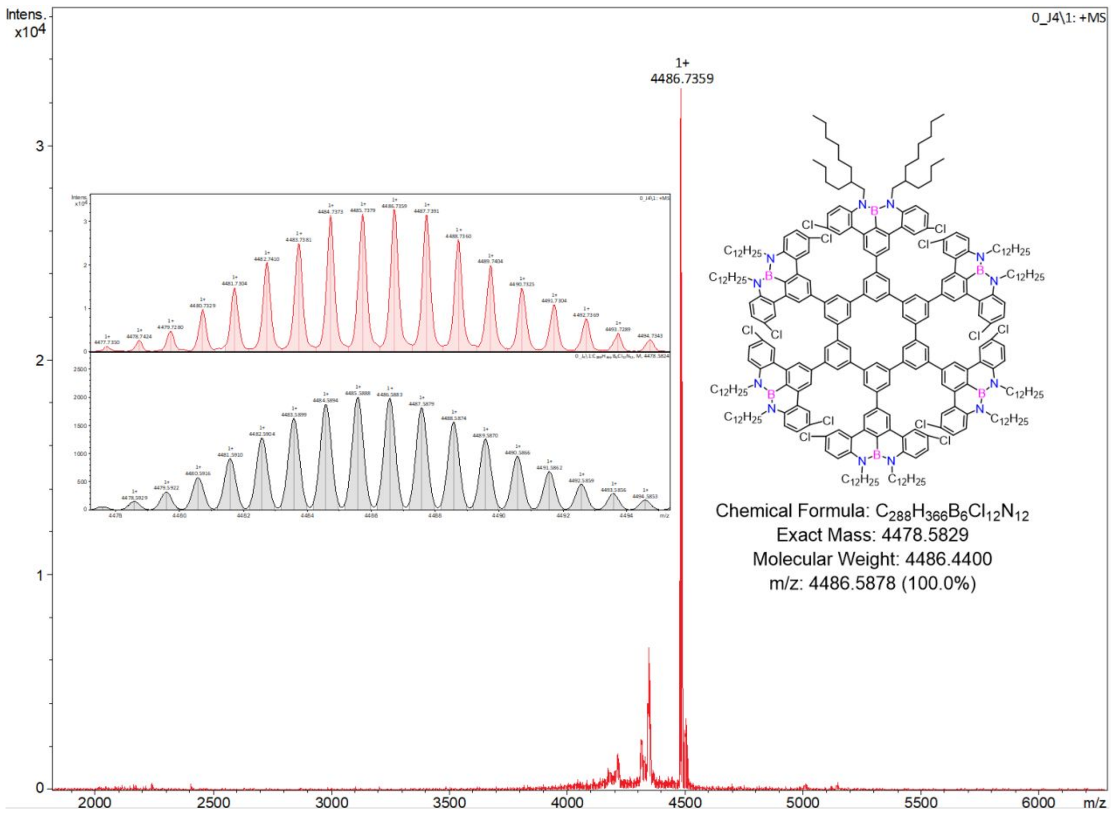

Figure S81. High resolution Maldi-tof-ms of compound **18**.

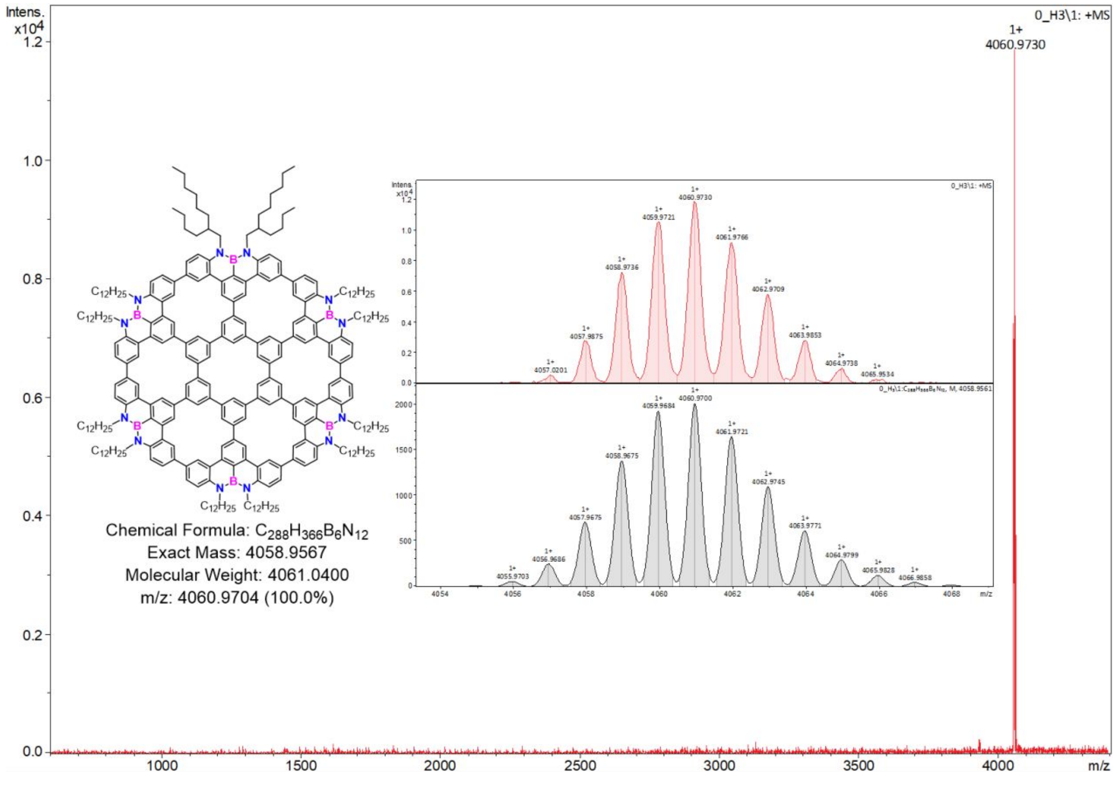

Figure S82. High resolution Maldi-tof-ms of **7PNG**.

9. References

(1) Su, S. J.; Tanaka, D.; Li, Y. J.; Sasabe, H.; Takeda, T.; Kido, J., Novel four-pyridylbenzene-armed biphenyls as electron-transport materials for phosphorescent OLEDs. *Org. Lett.* **2008,** *10*, 941-944.

(2) Yin, Y.; Zhang, Y.; Zhou, X.; Gui, B.; Cai, G.; Sun, J.; Wang, C., Single-Crystal Three-Dimensional Covalent Organic Framework Constructed from 6-Connected Triangular Prism Node. *J. Am. Chem. Soc.* **2023,** *145*, 22329-22334.

(3) Ikemoto, K.; Yoshii, A.; Izumi, T.; Taka, H.; Kita, H.; Xue, J. Y.; Kobayashi, R.; Sato, S.; Isobe, H., Modular Synthesis of Aromatic Hydrocarbon Macrocycles for Simplified, Single-Layer Organic Light-Emitting Devices. *J. Org. Chem.* **2016,** *81*, 662-666.

(4) Chen, Z. F.; Wannere, C. S.; Corminboeuf, C.; Puchta, R.; Schleyer, P. V., Nucleus-independent chemical shifts (NICS) as an aromaticity criterion. *Chem. Rev.* **2005,** *105*, 3842-3888.

(5) Schleyer, P. V. R.; Maerker, C.; Dransfeld, A.; Jiao, H.; van Eikema Hommes, N. J. R., Nucleus-Independent Chemical Shifts: A Simple and Efficient Aromaticity Probe. *J. Am. Chem. Soc.* **1996,** *118*, 6317-6318.

(6) Wolinski, K.; Hinton, J. F.; Pulay, P., Efficient Implementation of the Gauge-Independent Atomic Orbital Method for Nmr Chemical-Shift Calculations. *J. Am. Chem. Soc.* **1990,** *112*, 8251-8260.

(7) Zhao, Y.; Truhlar, D. G., The M06 suite of density functionals for main group thermochemistry, thermochemical kinetics, noncovalent interactions, excited states, and transition elements: two new functionals and systematic testing of four M06-class functionals and 12 other functionals. *Theor. Chem. Acc.* **2008,** *120*, 215-241.

(8) Ou, Q.; Subotnik, J. E., Electronic Relaxation in Benzaldehyde Evaluated via TD-DFT and Localized Diabatization: Intersystem Crossings, Conical Intersections, and Phosphorescence. *J. Phys. Chem. C* **2013,** *117*, 19839-19849.

(9) Epifanovsky, E.; Gilbert, A. T. B.; Feng, X. T.; Lee, J.; Mao, Y. Z.; Mardirossian, N.; Pokhilko, P.; White, A. F.; Coons, M. P.; Dempwolff, A. L.; Gan, Z. T.; Hait, D.; Horn, P. R.; Jacobson, L. D.; Kaliman, I.; Kussmann, J.; Lange, A. W.; Lao, K. U.; Levine, D. S.; Liu, J.; McKenzie, S. C.; Morrison, A. F.; Nanda, K. D.; Plasser, F.; Rehn, D. R.; Vidal, M. L.; You, Z. Q.; Zhu, Y.; Alam, B.; Albrecht, B. J.; Aldossary, A.; Alguire, E.; Andersen, J. H.; Athavale, V.; Barton, D.; Begam, K.; Behn, A.; Bellonzi, N.; Bernard, Y. A.; Berquist, E. J.; Burton, H. G. A.; Carreras, A.; Carter-Fenk, K.; Chakraborty, R.; Chien, A. D.; Closser, K. D.; Cofer-Shabica, V.; Dasgupta, S.; de Wergifosse, M.; Deng, J.; Diedenhofen, M.; Do, H.; Ehlert, S.;

Fang, P. T.; Fatehi, S.; Feng, Q. G.; Friedhoff, T.; Gayvert, J.; Ge, Q. H.; Gidofalvi, G.; Goldey, M.; Gomes, J.; González-Espinoza, C. E.; Gulania, S.; Gunina, A. O.; Hanson-Heine, M. W. D.; Harbach, P. H. P.; Hauser, A.; Herbst, M. F.; Vera, M. H.; Hodecker, M.; Holden, Z. C.; Houck, S.; Huang, X. K.; Hui, K.; Huynh, B. C.; Ivanov, M.; Jasz, A.; Ji, H.; Jiang, H. J.; Kaduk, B.; Kähler, S.; Khistyaev, K.; Kim, J.; Kis, G.; Klunzinger, P.; Koczor-Benda, Z.; Koh, J. H.; Kosenkov, D.; Koulias, L.; Kowalczyk, T.; Krauter, C. M.; Kue, K.; Kunitsa, A.; Kus, T.; Ladjánszki, I.; Landau, A.; Lawler, K. V.; Lefrancois, D.; Lehtola, S.; Li, R. R.; Li, Y. P.; Liang, J. S.; Liebenthal, M.; Lin, H. H.; Lin, Y. S.; Liu, F. L.; Liu, K. Y.; Loipersberger, M.; Luenser, A.; Manjanath, A.; Manohar, P.; Mansoor, E.; Manzer, S. F.; Mao, S. P.; Marenich, A. V.; Markovich, T.; Mason, S.; Maurer, S. A.; McLaughlin, P. F.; Menger, M. F. S. J.; Mewes, J. M.; Mewes, S. A.; Morgante, P.; Mullinax, J. W.; Oosterbaan, K. J.; Paran, G.; Paul, A. C.; Paul, S. K.; Pavosevic, F.; Pei, Z.; Prager, S.; Proynov, E. I.; Rak, A.; Ramos-Cordoba, E.; Rana, B.; Rask, A. E.; Rettig, A.; Richard, R. M.; Rob, F.; Rossomme, E.; Scheele, T.; Scheurer, M.; Schneider, M.; Sergueev, N.; Sharada, S. M.; Skomorowski, W.; Small, D. W.; Stein, C. J.; Su, Y. C.; Sundstrom, E. J.; Tao, Z.; Thirman, J.; Tornai, G. J.; Tsuchimochi, T.; Tubman, N. M.; Veccham, S. P.; Vydrov, O.; Wenzel, J.; Witte, J.; Yamada, A.; Yao, K.; Yeganeh, S.; Yost, S. R.; Zech, A.; Zhang, I. Y.; Zhang, X.; Zhang, Y.; Zuev, D.; Aspuru-Guzik, A.; Bell, A. T.; Besley, N. A.; Bravaya, K. B.; Brooks, B. R.; Casanova, D.; Chai, J. D.; Coriani, S.; Cramer, C. J.; Cserey, G.; DePrince, A. E.; DiStasio, R. A.; Dreuw, A.; Dunietz, B. D.; Furlani, T. R.; Goddard, W. A.; Hammes-Schiffer, S.; Head-Gordon, T.; Hehre, W. J.; Hsu, C. P.; Jagau, T. C.; Jung, Y. S.; Klamt, A.; Kong, J.; Lambrecht, D. S.; Liang, W. Z.; Mayhall, N. J.; McCurdy, C. W.; Neaton, J. B.; Ochsenfeld, C.; Parkhill, J. A.; Peverati, R.; Rassolov, V. A.; Shao, Y. H.; Slipchenko, L. V.; Stauch, T.; Steele, R. P.; Subotnik, J. E.; Thom, A. J. W.; Tkatchenko, A.; Truhlar, D. G.; Van Voorhis, T.; Wesolowski, T. A.; Whaley, K. B.; Woodcock, H. L.; Zimmerman, P. M.; Faraji, S.; Gill, P. M. W.; Head-Gordon, M.; Herbert, J. M.; Krylov, A. I., Software for the frontiers of quantum chemistry: An overview of developments in the Q-Chem 5 package. *J. Chem. Phys.* **2021,** *155*, 8.

(10) Plasser, F., TheoDORE: A toolbox for a detailed and automated analysis of electronic excited state computations. *J. Chem. Phys.* **2020,** *152*, 084108.
